# Enhancing structural diversity through chemical engineering of *Ambrosia tenuifolia* extract for novel anti-glioblastoma compounds

**DOI:** 10.1038/s41598-024-63639-y

**Published:** 2024-06-20

**Authors:** Tonino G. Adessi, Paula M. Wagner, Fabricio R. Bisogno, Viviana E. Nicotra, Mario E. Guido, Manuela E. García

**Affiliations:** 1 Facultad de Ciencias Químicas, Universidad Nacional de Córdoba (UNC), Edificio de Ciencias Químicas 2, Haya de la Torre y Medina Allende, Ciudad Universitaria, CP X5000HUA Córdoba, Argentina; 2 Instituto Multidisciplinario de Biología Vegetal (IMBIV), Consejo Nacional de Investigaciones Científicas y Técnicas (CONICET), Córdoba, Argentina; 3 Departamento de Química Biológica Ranwel Caputto, Centro de Investigaciones en Química Biológica de Córdoba (CIQUIBIC-CONICET), Córdoba, Argentina; 4 Instituto de Investigaciones en Físico-Química de Córdoba (INFIQC-CONICET), Córdoba, Argentina

**Keywords:** CNS cancer, Secondary metabolism, Medicinal chemistry, Organic chemistry

## Abstract

Natural products are an unsurpassed source of leading structures in drug discovery. The biosynthetic machinery of the producing organism offers an important source for modifying complex natural products, leading to analogs that are unattainable by chemical semisynthesis or total synthesis. In this report, through the combination of natural products chemistry and diversity-oriented synthesis, a diversity-enhanced extracts approach is proposed using chemical reactions that remodel molecular scaffolds directly on extracts of natural resources. This method was applied to subextract enriched in sesquiterpene lactones from *Ambrosia tenuifolia* (Fam. Asteraceae) using acid media conditions (*p*-toluenesulfonic acid) to change molecular skeletons. The chemically modified extract was then fractionated by a bioguided approach to obtain the pure compounds responsible for the anti-glioblastoma (GBM) activity in T98G cell cultures. Indeed, with the best candidate, chronobiological experiments were performed to evaluate temporal susceptibility to the treatment on GBM cell cultures to define the best time to apply the therapy. Finally, bioinformatics tools were used to supply qualitative and quantitative information on the physicochemical properties, chemical space, and structural similarity of the compound library obtained. As a result, natural products derivatives containing new molecular skeletons were obtained, with possible applications as chemotherapeutic agents against human GBM T98G cell cultures.

## Introduction

Access to libraries of molecules capable of representing and populating the biologically relevant chemical space is a critical step in the drug discovery process. In this sense, due to a long process of evolution, natural products (NPs) are recognized as biologically validated starting points for the development of libraries of bioactive molecules^1,2^. NPs possess a unique chemical diversity that complements synthetic collections. They exhibit greater steric complexity and a wider variety of ring systems compared to synthetic and combinatorial chemistry compounds. Less than one-fifth of the ring systems found in nature are represented in current commercial drugs, which tend to include a greater number of nitrogen, sulfur, and halogen heteroatoms. Therefore, the complementarity of natural and synthetic machinery is truly invaluable. Chemists have successfully generated semisynthetic analogs that possess more activity, selectivity, or less toxicity than the original natural product^3,4^.

**Figure 1:**
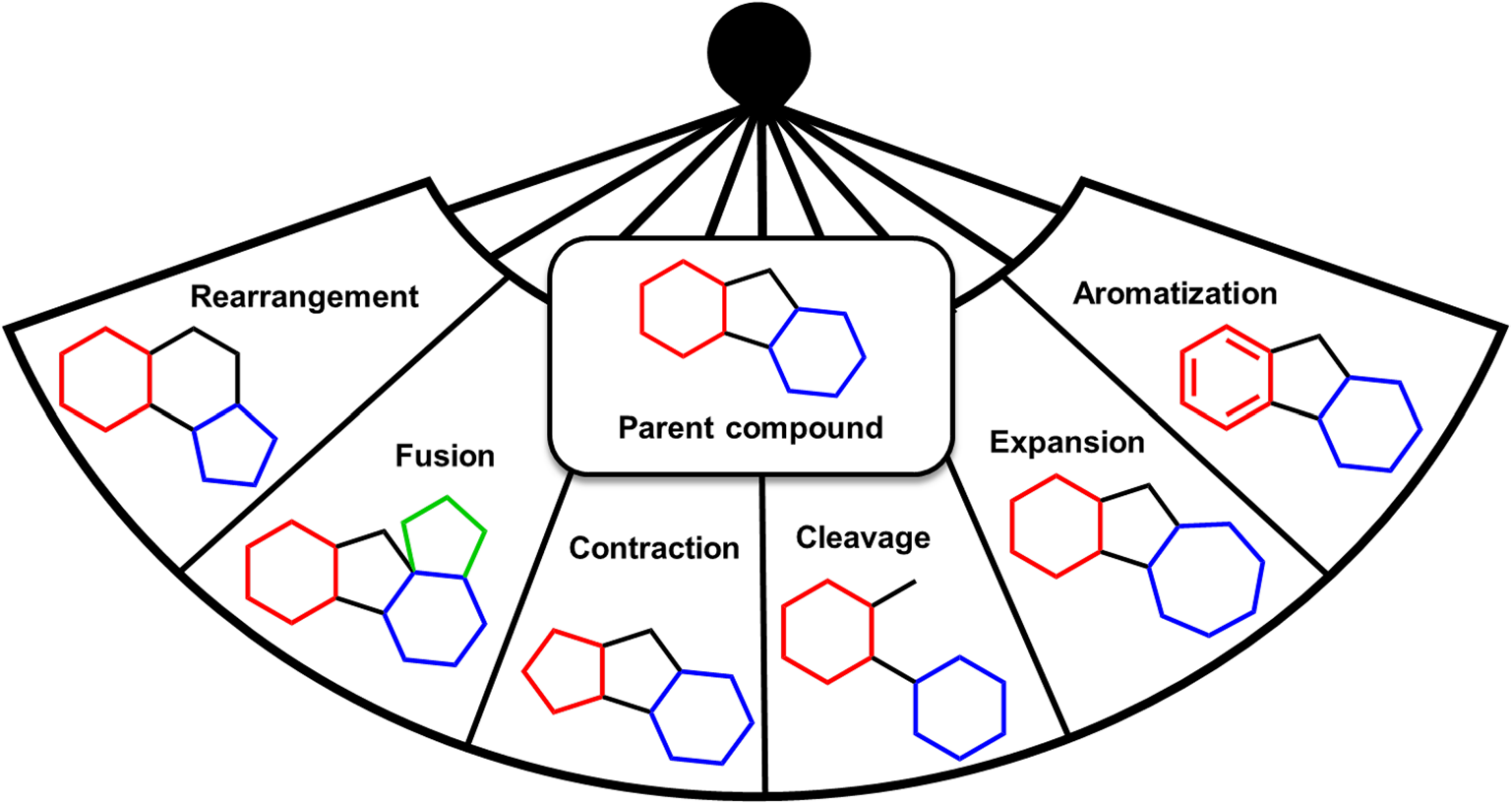
Complexity to diversity strategy: most common ring distortion reactions.

Diversity oriented synthesis (DOS) strategies are efficient methodologies for constructing diverse compounds from simple precursors^5^. In this sense, to take advantage of the distinctive characteristics of NPs by harnessing the power of organic synthesis, a new DOS approach has recently emerged for the rapid creation of complex and diverse small molecules, which constitutes a great challenge around synthetic, medicinal, and biological chemistry. In this Complexity to Diversity paradigm^6,7^. NPs undergo a series of ring distortion reactions to generate new structures that are complex and diverse from the original natural product and from each other. This process plays a fundamental role in exploring the biologically relevant chemical space. These ring distortions (Fig. 1) include ring expansions, contractions, fusions, cleavages, and rearrangements.

Applying this methodology to the direct chemical modification of an extract can yield mixtures containing fragments or elements conducive to bioactivity, thus modifying the functional groups in original NPs or their molecular skeletons. To date, there are not many reports of this latter methodology, so diversity-enhanced natural extracts represent a very challenging approach. Thus, nature's biosynthetic machinery can be strengthened in a single step by applying reactions that form new carbon–carbon bonds and modify molecular scaffolds^8–11^. Therefore, compounds like natural products with a wide chemical diversity are obtained in a single step through a simple reaction, *divergently*.

At this point it is important to mention that the chemical complexity of the extracts (unmodified or modified) often warrants the use of a bioguided fractionation model capable of leading to bioactive compounds in a rational way, using a measure of biological activity of interest. In this sense, bioguided assays based on extracts or fractions of interest can provide information that serves as a guide to determine how to intensify fractionation and purification *convergently*, also allowing the discovery of bioactive compounds not previously described. These metabolites are generally found in smaller amounts and are less likely to be isolated in a traditional phytochemical study. It remains to point out that in this type of approach, valuable synergies, additive effects, and antagonisms are often observed (which are often undetected using other approaches). This makes it a useful approach for exploring the most biologically relevant fractions in a few steps and more deeply ^12–14^.

Currently, the biggest challenge when it comes to developing effective therapeutic agents is the identification of new chemotypes for drugs, especially in the case of antimicrobials and antitumoral drugs. Among the natural compounds, terpenoids display a wide range of biological activities, an exceptional group of them, is constituted by the sesquiterpene lactones (SLs)^15^. These represent one of the most relevant groups of metabolites isolated from the Fam. Asteraceae. These compounds commonly have an α-methylene-γ-lactone group in their structure, which is considered essential for their activity^16,17^. This chemical functionality is often responsible for biological effects, and in some cases, cytotoxic activity is attributed to this feature. To date, significant advances have been made in the chemical modifications of SLs, allowing the successfully modulation of their therapeutic properties^18^. It is noteworthy that they have a long history as anticancer agents^19^, showing significant cytotoxic activity and selectivity against different tumor cell lines (some SLs and their semi-synthetic derivatives are currently being tested in clinical trials)^20,21.^

In this sense, one of our goals was to evaluate if these SLs have an anti-glioblastoma activity in a glioma cell culture model. Gliomas originated from different glial cell types are solid tumors of the Central Nervous System (CNS) that share histological features with astrocytes, oligodendrocytes, and ependymal cells^22,23^. The World Health Organization classified CNS tumors into four groups^24^ with increasing malignancy according to morphological and histochemical features. In particular, glioblastoma (GBM) is a highly invasive and vascularized tumor classified as grade 4 adult-type diffuse glioma^25^. The incidence of this tumor is 3.19 cases per 100.000 population and the mean age is 64 years at diagnosis^26^. Even when the standard treatment named Stupp protocol (surgery and radiochemotherapy) is applied, GBM are resistant tumors to conventional treatment with poor prognosis^27^. The average survival is 12–15 months, with 6.8% reaching 5 years after diagnosis^28^. Therefore, innovative strategies are needed to increase the quality of life and survival of patients. In this context, chronotherapy proposed a differential temporal administration of drugs according to the timing imposed by the intrinsic circadian clock to improve drug efficiency and minimize side effects of the treatment^29^. Remarkably, the circadian clock present in cells of the whole organism, including tumor cells, temporally controls behavior and physiology while the disruption of circadian rhythms as a consequence of modern life, continuous artificial illumination, jet lag, shift work and others, may severely affect cell metabolism causing metabolic disorders and a higher cancer risk^30–34^.

Recently, CNS tumors exhibited a differential response to bortezomib (proteasome inhibitor) in both an in vivo murine model and glioma cell cultures (T98G and A530 cells) according to the daytime that were severely affected after the circadian clock disruption^35,36^. Indeed, its effect on cell viability was highly potentiated when administered with SR9009 (an agonist for the nuclear receptor REV-ERB)^37^.

In addition, temozolomide (TMZ) administration in human and murine GBM cells in culture was dependent on clock gene expression^38^. Natural or modified plant extract or pure compound administration could be a potential strategy to treat GBM under a chronobiological base. NPs are commonly known for their diverse molecular and cellular mechanisms at various sites of tumorigenesis. They are currently considered ideal for evaluation in GBM, either alone or in combination with chemotherapeutic agents^39,40^.

Considering the background of the antiproliferative activity of different sesquiterpene lactones, the main metabolites of the plant species *Ambrosia tenuifolia* Spreng. (Asteraceae), the derivatization of a plant extract was performed by a simple chemical reaction leading to obtain diversity and structural complexity. Bioguided fractionation of the modified extract was performed, analyzing the anti-glioblastoma activity and time-related susceptibility on GBM cell cultures.

## Results and discussion

### Extract derivatization and bioguided fractionation

**Figure 2:**
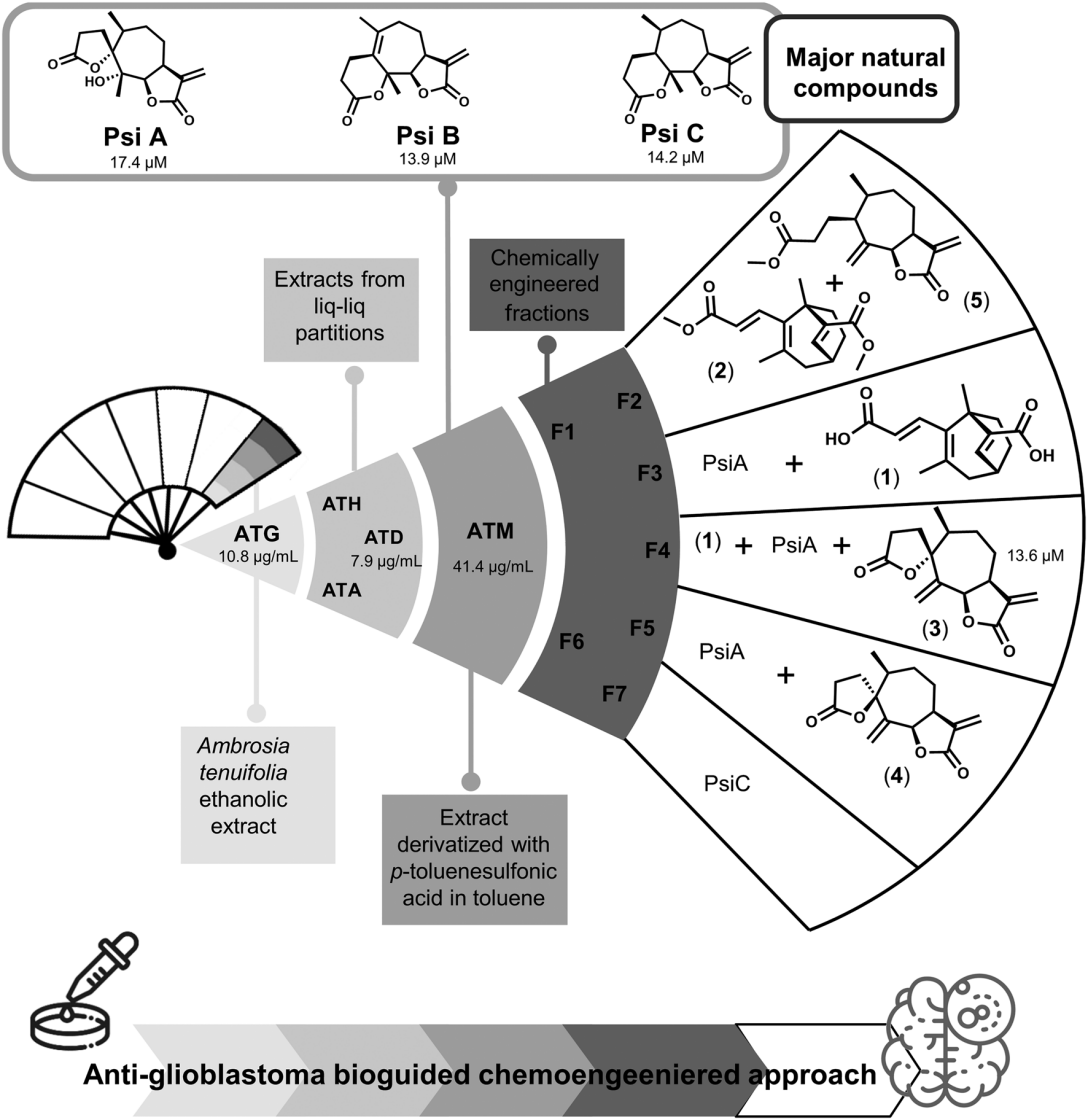
CtD structural “hand fan” approach: schematic representation of bioguided fractionation of DCM *p*-toluensulfonic acid modified extract of *Ambrosia tenuifolia* to isolate and identify the main compounds responsible for the anti-glioblastoma activity. IC_50_ values were evaluated in T98G (GBM cells). All fractions and compounds were evaluated to follow the proposed convergent biofractionation scheme; for interpretation purposes only IC_50_ < 100 µM are showed.

**Figure 3:**
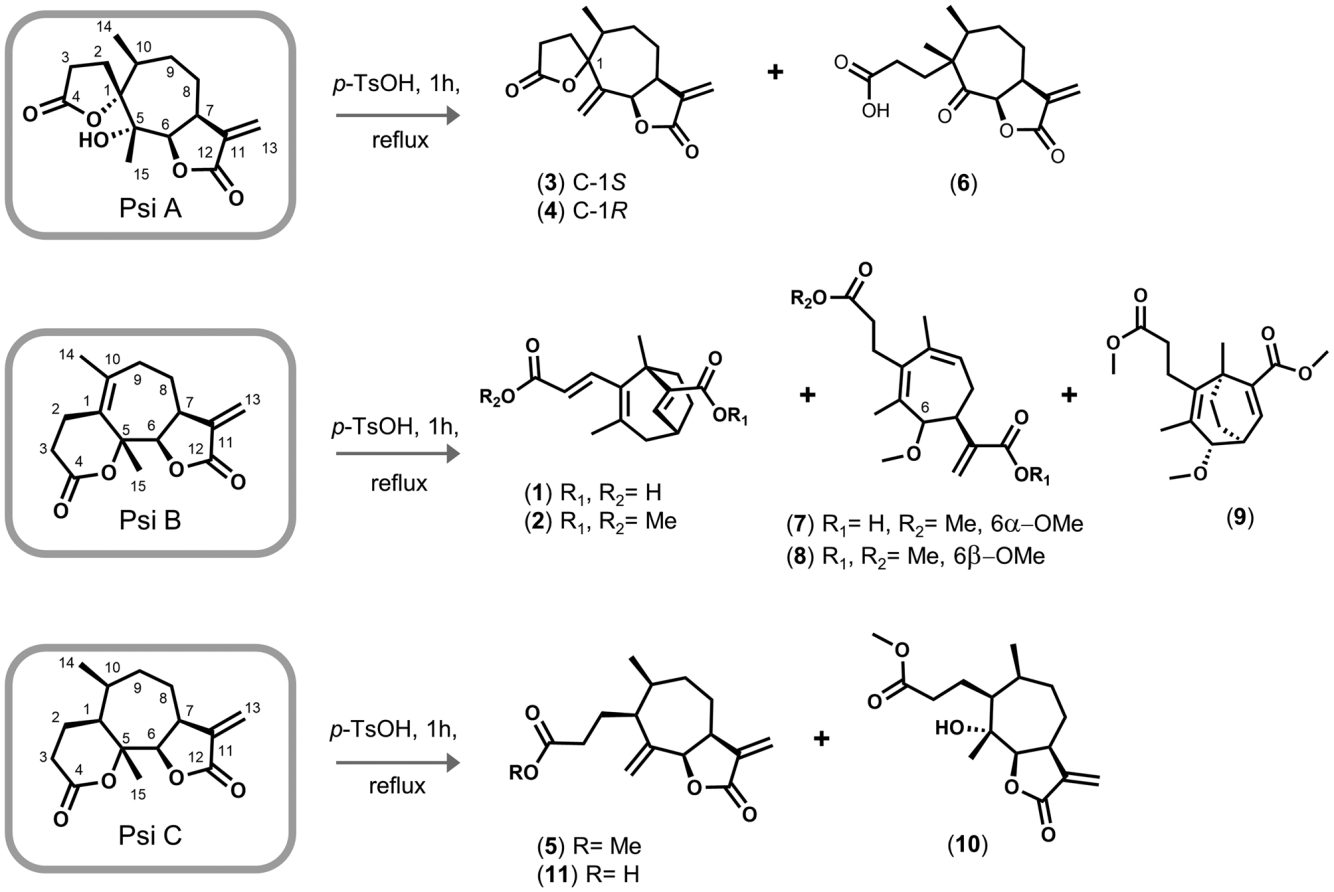
Complexity to diversity products obtained from pure substrates. The isolated yields are shown in the spectroscopic description of each compound (experimental section).

To perform this study, the global ethanolic extract of *Ambrosia tenuifolia* (ATG) was obtained. In previous reports, the phytochemical exploration of this species was carried out, so the composition of the dichloromethane extract (ATD) was verified, being the psilostachyins (Psi) A, B, and C the main components thereof^41,42^. The structures of the isolated SLs (Figs. 2 and 3) were established based on their NMR data and by comparison with those previously reported^43^.

This synthetic approach acknowledges that the spatial arrangement of functional groups is often crucial for interactions with biological targets. So, the installation of molecular complexity in this semisynthetic strategy further allows for the tailoring of chemical properties, such as size, shape, and polarity, for the generation of focused libraries with acceptable bioavailability. As a result, the selection of NPs containing sites for diversification or the introduction of new functional groups during the transformation process is crucial. The reorganization of NPs into complex and diverse scaffolds is a highly complementary technique to NP chemical engineered extract to sufficiently populate screening libraries with complex small molecules. This approach is essential for the discovery of compounds that can effectively interact with challenging targets and explore the biologically relevant chemical space.

As a NP for transformation into complex and diverse structures, sesquiterpene lactones has helpful features that enable several divergent transformations across all the ring systems. These include hydroxyls, carbonyls, lactones, and olefins on the rings. Beginning from classical degradation reactions using different acids, able to hydrolyze or transesterify cyclic esters, initially producing opening of lactone rings, dehydration of alcohols, and formation of carbocations, highly distorted products are obtained.

For this purpose, the global ethanolic extract of *A. tenuifolia* (ATG) was partitioned with solvents of increasing polarity, yielding three subextracts of different polarity [hexane (ATH), CH_2_Cl_2_ (ATD) and EtOAc (ATA)]. From these, the ATD extract held the greater concentration of SLs, (PsiA 21.8%, PsiB 47.3% and PsiC 19.5%, evidenced by ^1^H-NMR). Based on our previous experience with the chemical modification of this extract, the dichloromethane extract was treated with *p*-toluenesulfonic acid in toluene (reflux conditions for 1 h)^44^. This extract (ATM) was subjected to bioguided fractionation as depicted in Fig. 2, following their antiglioblastoma activity (as described in Fig. 2 and S4). The diversity-enhanced extract of *A. tenuifolia* was chromatographed to yield a set of pure compounds (some with unprecedented skeletons) suitable for biological assays with some containing unprecedented molecular skeletons.

As can be seen, PsiB was completely consumed with respect to its congeners PsiA and PsiC, which denotes its differential reactivity. If the content of the fractions obtained is carefully and exhaustively analyzed, after their purification, the metabolites can be grouped according to the compound from which they are derived. All the details corresponding to the complete structural elucidation of the derivatives obtained are found in the supplementary information.

Thus, in fraction F2, as shown in the proposed mechanism of Scheme S1 (supplementary information), by obtaining compounds **1** and **2** an annular distortion is achieved in all cycles of the molecule. This could be obtained by opening of the lactone of 6-membered and the rearrangement of the 7- and 5-membered cycles to form a bicycle, through carbocation rearrangements promoted in an acidic medium.

Regarding PsiA, its presence was observed in three of the five fractions collected, as well as it was the precursor of two epimeric derivatives (compounds **3** and **4**), present in fraction F4 and F5, respectively. Compound **3** has been previously described by Mabry et al. obtained by derivatization of PsiA to perform spectroscopic characterization of the natural compound^45^.

Furthermore, the presence of PsiC, as the only constituent of fraction F7, and in fraction F2 compound **5,** a derivative of PsiC, (previously reported), were detected. Their spectral data are according to the literature (Fig. S7-1) as reported by Kagan et al. ^46^*.*

Although for compounds **3**, **4** and **5** the base skeleton was not remodeled, key structural motifs were added or changed (either unsaturation or epimerization of a stereocenter) that drastically alter the global geometry of the skeleton.

In this way we verified that the reengineering of this extract allowed us to obtain ring distortion through a simple one-step chemical reaction.

To assess the efficiency of the proposed methodology (in terms of quantity and proportion of products obtained in a reaction step from an extract or mixture), the ring distortion model reaction was achieved on each pure substrate, PsiA, B and C. Indeed, other purpose of perform these “control” reactions was to make a comparative assessment to figure out whether the higher concentration and/or reactivity of PsiB are truly the primary factor behind the greater number of products derived from it, potentially masking the chemistry of the two remaining lactones, PsiA and PsiC.

In this way, we were able to obtain the bioactive compounds of the synthesized series, and we also obtained novel distorted compounds (Fig. 3). This also enable us to propose a plausible synthetic route to obtain some rearranged products (see supplementary information Schemes S2 and S3).

For PsiA, **3** and **4** derivatives were obtained, in good yields, making this reaction a feasible protocol for the synthesis of the leading bioactive compound of the series. Furthermore, compound **6** is obtained, a structure not described in the literature. This derivative presents a lactone ring opening and a carbonyl group that supplies a differential electronic density at C-5 (see proposed mechanism in Scheme S2, supplementary material).

In the case of PsiB, the most reactive substrate of the series, evidenced by the large number of rearranged derivatives produced in a single reaction step, in addition to the compounds **1** and **2**, others structurally diverse and complex derivatives were obtained, which are described spectroscopically in detail in supplementary material. For this series, a high annular distortion is evident along with a possible obtaining mechanism for compound **9**, see scheme S3 in supplementary material.

Finally, the protocol applied to PsiC leads to the formation of three structurally closely related products derived from a lactone ring opening. Thus, compounds **5** (which was obtained by derivatizing the extract), **10** (previously described in the literature ^46^, spectral data in agreement with those reported, Fig. S12-1), and **11** (novel compound) were obtained.

In general terms, the derivatization of the pure compounds allows us to explain the reason why PsiA and C are recovered in the derivatization of the extract (due to the greater reactivity of Psi B and/or due to the lower relative amount of these precursors in the extract) and allows us to propose more accurate synthetic routes.

### Biological assays

The activity of the extracts, fractions and pure compounds on cell viability was evaluated in vitro throughout the entire biofractionation process in T98G cells derived from a human glioblastoma.

In general terms, the proposed workflow, which is based on the conjunction between both *divergent* (extract derivatization)/*convergent* (bioguided fractionation) strategies, leads to obtaining bioactive products, which is the aim pursued.

**Figure 4:**
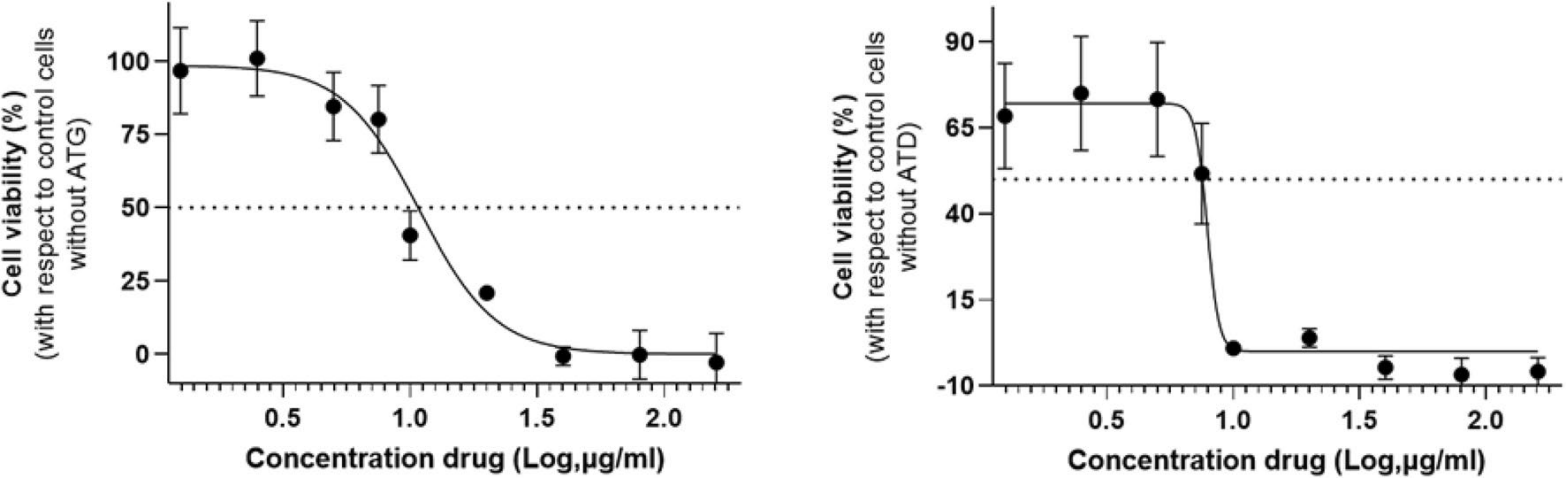
Dose–response curve of crude extract from *Ambrosia tenuifolia* (ATG) or dichloromethane extract (ATD) in T98G cells. Cultures were treated with ATG or ATD in a range of concentrations (1.25, 2.5, 5, 7.5, 10, 20, 40, 80, and 160 µg/mL) for 24 h and cell viability was analyzed by MTT assay. Half maximal inhibitory concentration (IC_50_) calculated using GraphPad Prism software revealed a value of the same order of magnitude (10.84 and 7.88 µg/ml for ATG (left graph) and ATD (right graph), respectively).

The ATG was analyzed, showing an interesting result in the μg/mL range (IC_50_ 10.84), followed by partition by classical polarity, which in turn leads to the most bioactive fraction (ATD, which concentrates the SLs, IC_50_ = 7.88 μg/mL, Fig. 4), which is therefore selected to be derivatized through the reaction of interest (see Table 1 and Fig. S1 for ATH and ATA extracts activity). The fractions obtained from the modified ATM extract showed less activity than the mixture of pure precursors, although when purified, in some cases a substantial increase in anti-glioblastoma activity was obtained.

**Table 1:** IC_50_ values in T98G cells of crude/chemically modified extracts and pure compounds.

		IC_50_	R^2^
Extract	ATG	10.8 μg/mL	0.705
	ATD	7.9 μg/mL	0.566
Compound	PsiA	17.4 μM	0.837
	PsiB	13.9 μM	0.807
	PsiC	14.2 μM	0.790
	(**3**)	13.6 μM	0.743
Standard treatment	Temozolomide	844.0 μM	0.960

GraphPad Prism software was used to calculate the IC_50_ (half maximal inhibitory concentration) value of each extract or compound. R^2^ value indicate how well the experimental data fit the regression model.

**Figure 5:**
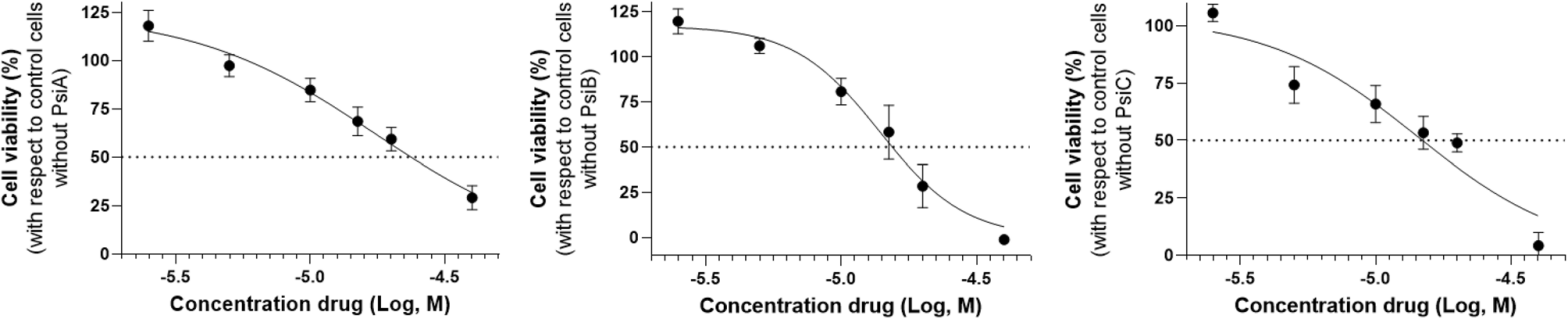
Dose–response curve of T98G cell viability after the treatment with psilostachyins A (PsiA), B (PsiB), and C (PsiC). Cells were incubated with the psilostachyins for 24 h and cell viability was measured by MTT assay. Results showed an IC_50_ value of 17.4 μM, 13.9 μM, and 14.2 μM for PsiA (left graph), PsiB (middle graph), and PsiC (right graph), respectively.

**Figure 6:**
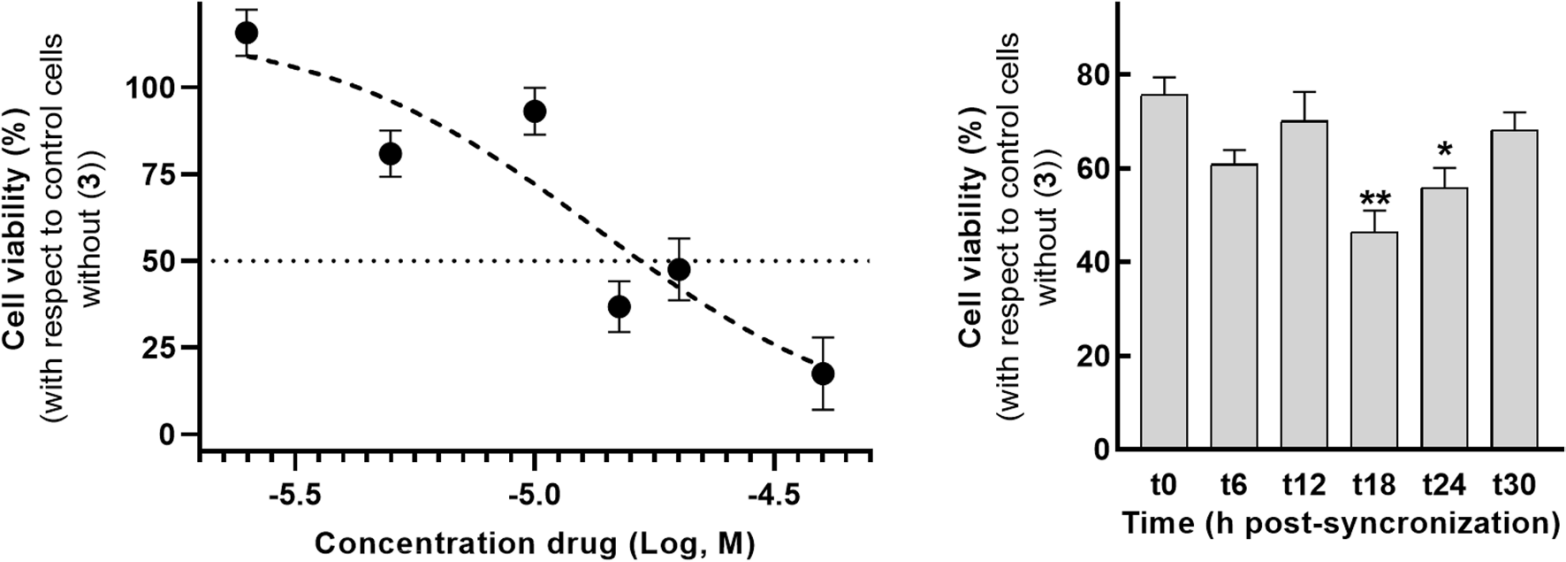
Effects of compound **3** on T98G cell viability. Left graph: Dose–response curve of compound **3** in T98G cells in which cultures were treated with compound **3** concentrations from 2.5 to 40 μM for 24 h and evidence an IC_50_ value of 13.6 μM. Right graph: Cells were synchronized with dexamethasone (100 nM, 1 h 37ºC) at time 0 and **3** was added to the cultures for 24 h at each time post synchronization along 30 h. Results showed a significant effect of time on cell viability treated with compound **3** (**p < 0.005, *p < 0.05, Kruskal Wallis test).

At this point, natural product derivatives with new molecular skeletons were obtained through this divergent-convergent approach with possible applications as chemotherapeutic agents against human GBM T98G cells. Here it is important to note the considerable activity of the pure precursor compounds (IC_50_ = 17.4, 13.9, and 14.2 μM for PsiA, PsiB, and PsiC, respectively, Fig. 5), which in some cases gives good activity to the mixed fraction that contains them and is then lost in the individualized pure compounds. The most remarkable result is that showing the most active derivative of the series **3**, (Table 1, Fig. 6), epimer of **4**, (Fig. S1) in position C-1 which was less active, which highlights a valuable structure–activity relationship in this type of SLs previously unexplored as anti-glioblastoma compounds. The remaining compounds (**1**), (**2**), (**5**), and (**11**) obtained from the bioguided procedure showed weak anti-glioblastoma activity in T98G cultures (Fig. S2).

Due to their activity, compound **3** was selected as the most active of the synthesized panel (IC_50_ = 13.6 µM) for the evaluation of temporal susceptibility on cell viability of GBM cultures. Differential administration of the drug according to time of treatment after synchronization could substantially improve therapy efficacy and reduce side effects. In fact, results evidenced significant lower levels of cell viability at 18 and 24 h after synchronization compared to cultures at time 0 without synchronization. Remarkably, non-synchronized cells exhibited the highest levels of viability (~ 75%), levels that remained elevated during the first 12 h post synchronization, while viability levels at 18 and 24 h post synchronization (45–50%) were the times at which cells displayed more susceptibility to the treatment of compound **3**, see Fig. 6.

The most active derivative of the series **3** along with its precursor (PsiA) were evaluated against a non-tumor model, using Human Embryonic Kidney cells (HEK 293). Only **3** showed a good selectivity index (IC_50_ HEK293 = 39 µM, Fig. S2). Indeed, temozolomide which is the standard therapy to treat GBM show an IC_50_ of 844 µM in T98G cultures. All these results are really encouraging and provide the basis for future synergy and chronotherapeutical studies with different SLs, capable of design novel approaches or improve the current strategies to the treatment of GBM.

Globally, regarding the GBM model, this work describes the use of several natural and chemically modified compounds from a diversity-enhanced extract of *Ambrosia tenuifolia* to look for a novel chemotherapeutic treatment of brain tumors.

Taking into consideration the functioning of the intrinsic cancer cell clock to find the best temporal window to apply a particular chemotherapy, in a series of complementary experiments, we found at least one of the PsiA derivatives, displaying a significant time-related variation in susceptibility of T98G cell viability to the compound administration as previously reported for the treatment with bortezomib or SR9009 in diverse GBM models.

### Chemoinformatic analysis

In general terms, starting from a mixture of easily available SLs, highly distorted, structurally, and stereochemically complex derivatives were obtained in a single reaction step.

Besides, each of these compounds has sites for diversification, allowing the easy and rapid creation of dozens of complex compounds in subsequent steps.

It is well known that NPs have great chemical diversity, and their chemical space is clearly distinguished from the space of synthetic compounds^47^. In this sense, the advances in cheminformatics allow the simultaneous evaluation of large chemical and biological data sets, with the aim of correlating the structural characteristics of small molecules with their biological activity. This analysis is often essential, since many compounds in the selection collections turn out to be false positives due to their physicochemical and structural characteristics, complicating the future development of new therapeutic agents^48^. For this reason a global qualitative and quantitative analysis of the structural similarity (through the calculation of the Tanimoto coefficient)^49^, physical–chemical properties^50^ and chemical space generated ^51,52^ was performed on all the derivatives obtained, Psi. D. Library, using cheminformatics tools^53^.

**Figure 7:**
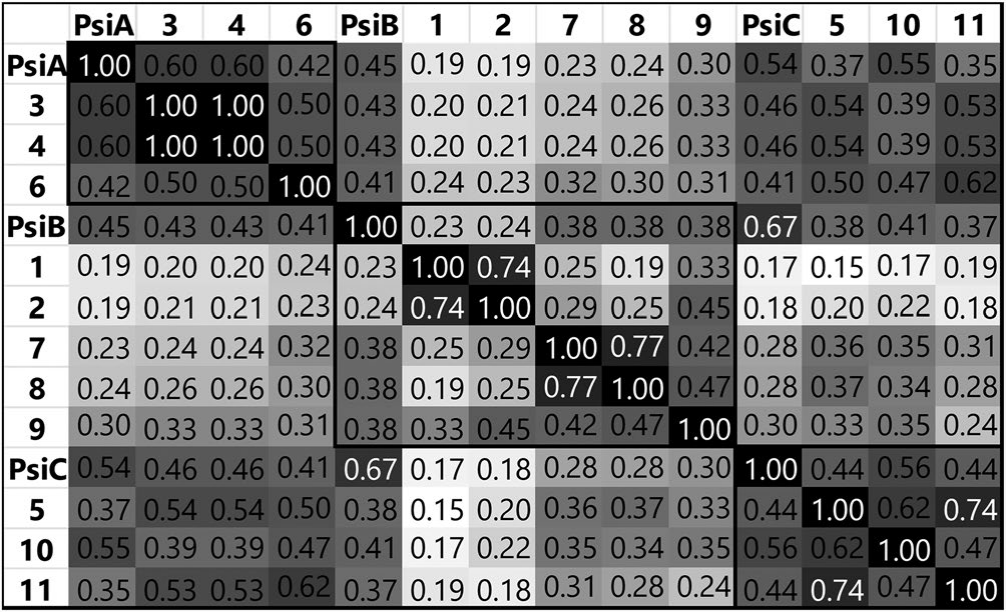
Evaluation of structural similarity between the compounds obtained using Tanimoto coefficients. Direct comparisons of the families obtained from each natural precursor PsiA, B and C are shown in boxes.

Regarding the quantitative analysis using structural similarity or Tanimoto coefficients, the ChemMineTools platform was used, which evaluates similarity using molecular “fingerprints”. A comparative matrix was obtained between all compounds with a similarity score for each correlated pair, on a scale from 0 to 1 (white to black in Fig. 7), where 1 represents perfect similarity. Here the structural descriptor AP (pairs of atoms) was used, which describes the shortest bonding paths between atoms (other than H) in a molecule. An average “variability” of 0.37 was obtained and the synthesized family presented a T range of 0.17 to 0.77, which reflects high structural diversity^54^.

On the other hand, physicochemical descriptors as well as to predict pharmacokinetic properties, druglike nature and medicinal chemistry friendliness were studied^55^, using the SwissADME website. Some of these parameters are lipophilicity, weight, polarity, insolubility, unsaturation, and flexibility; that allow us to know an approximation of the oral bioavailability of the compounds and in turn these properties allow us to construct the “rule of 5” or Lipinski's Rule, which is used when qualitatively evaluating the potential of a chemical compound to become in a lead or drug candidate. The results are shown in Table S1 and S2 (supplementary information). In general terms, all the compounds analyzed show optimal calculated parameters of oral bioavailability, being interesting candidates for future evaluations. A result worth highlighting is that the precursors (PsiA, B and C) and **3** (the compounds that show the best biological activity) are those that show the least flexibility of the entire series obtained by bioguided fractionation. In this context, another of the most valuable tools of this analysis is the ability to predict whether the compounds obtained will be able to cross the blood–brain barrier (BBB)^56^. In this case, all the compounds obtained (except PsiA and **6**) would have brain penetration (see supplementary information Fig. S14).

**Figure 8:**
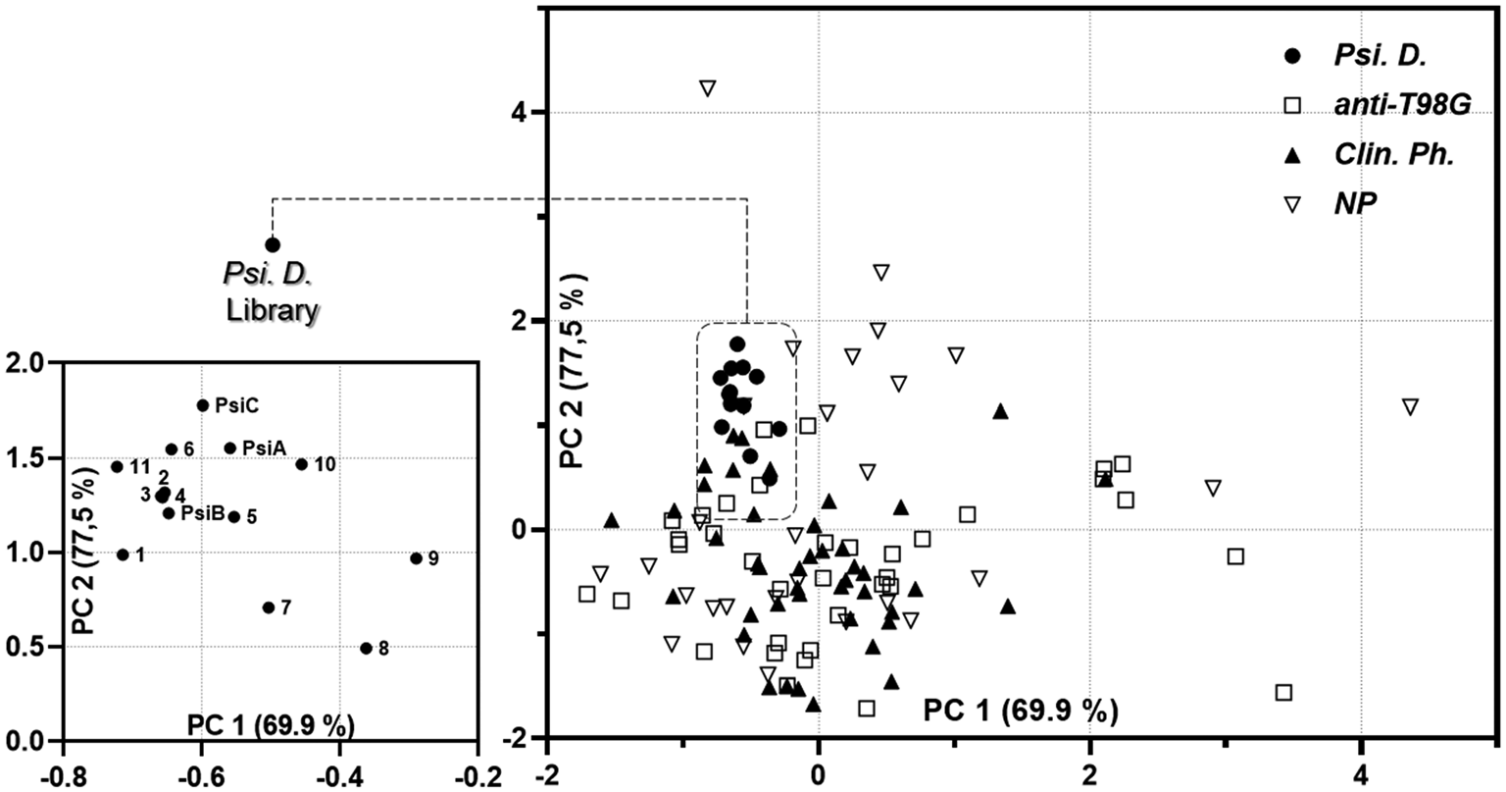
2D visual representation of the chemical space of different compound libraries according to the origin of the compounds. The visual representation was generated with a principal component analysis of 83 descriptors.

Furthermore, to navigate the vast diversity of chemical space, the concept of "chemography" has been proposed, which is like a global positioning system. This involves mapping compounds to coordinates of molecular descriptors^57^ of various physicochemical and topological properties revealing complete information about molecules and are easy to obtain. In this sense, bioactive compounds are only found in a fraction of the chemical space, known as the biologically relevant chemical space, so, by analyzing the library obtained, we seek to know if it was possible to occupy and expand this space, to through the structural transformations carried out. In this case the studies were conducted using the MOE (Molecular Operating Environment) platforms^58^. Many times, to describe chemical libraries (prior to biological activity assays), physicochemical properties are used to represent the occupied chemical space. To illustrate a visual representation of the chemical space based on properties, Fig. 8 shows the comparison of the set obtained in this work, with respect to different libraries selected for this aim. In the chemical space mapping, the system was explained by the first 2 principal components with a variance of 77.5% and 83 descriptors, see supplementary information Table S4.

These ring distortion transformations verified the occupation and expansion of the biologically relevant chemical space; the magnification of Fig. 8 illustrates that ring distortions enable the exploration of the chemical space around the natural starting products, the psilostachyins, resulting in structural diversity, as analyzed with the Tanimoto coefficients. Moreover, the placement of antiglioblastoma molecules from other libraries in the space generated by principal component analysis allows us to observe that our library, *Psi D*, occupies a previously uncharted area of the biologically relevant chemical space. Additionally, a cluster analysis (Fig. S18 in the supplementary material) confirmed that the compounds in this library form a distinct cluster, including only 14 other antiglioblastoma compounds out of a total of 107 molecules analyzed.

Finally, the enhanced diversity of extracts was achieved through simple chemical modifications able to reshape the naturally occurring molecular scaffolds through a one-step chemical reaction. Thus, by acid-catalyzed rearrangement and ring distortion reactions in the molecular skeletons, the different extracts chemical composition was modified, complementing the biosynthetic machinery of the plant species. Indeed, bioguided fractionation assays for anti-glioblastoma activity was applied. This strategy can provide relevant information as a guide for determining how to intensify fractionations and purifications. It is important to be able to efficiently combine and link both methodologies because extract reengineering aims to divergently increase structural complexity and diversity, creating new chemical entities that can be derivatized in subsequent stages. Thus, it requires a complementary convergent strategy, such as biodirected fractionation to end up at pure bioactive compounds.

## Conclusions

As a result of applying this *divergent-convergent* approach, more than ten sesquiterpenoids were unpredictably obtained; these compounds have unique and unprecedented ring systems. Such structures are challenging to obtain through rational or sequential diversity-oriented synthesis. Therefore, this methodology, capable of originating this unexpected collection of compounds in one step highlights one of the advantages of extracts improved by diversity.

Of course, the methodology poses great challenges. After reactions that generate diversity, the reaction crudes become complex mixtures; this issue is circumvented by introducing convergence through the biodirected approach which focuses only on bioactive fractions.

The methodology for diversity-enhanced extracts reported here, is a good diversity tool to create and discover new compounds and skeletons in “one shot” by a single step, simple, inexpensive, versatile, and quick reaction. If biofractionation is added as a screening method, the advantages lie on the time saving, along with obtaining beneficial synergies in the search for bioactive compounds of natural origin.

Thus, we were able to describe for the first time the application of extracts of increased diversity as a novel chemotherapeutic strategy in GBM, evaluating the chronotherapeutic potential of the most active compounds.

This body of evidence allows us to propose the challenge of new therapeutic strategies for treating brain tumors like GBM. These strategies consider the potential synergic combination of different derivatives and natural compounds, as well as the circadian modulation of treatment, aiming to improve efficacy and reduce side effects.

Finally, this strategy evaluated as a “proof of concept” on SLs like bioactive skeletons, leads to a synthetic library involving a wide chemical diversity in terms of chemical skeletons, which can lead to a diversification of biological activities. Thus, the potential and versatility of the proposed extract chemical engineering methodology, are expanded. Therefore, future projections involve the rational search for other possible biological targets using different bioinformatics tools.

## Experimental

### General

Optical rotation was measured on a JASCO P-1010 polarimeter. IR spectra were obtained in a Nicolet iZ10 ThermoScientific spectrophotometer (each compound was dissolved in a minimum amount of solvent, and a drop of solution was added to the AgCl IR plates). NMR experiments were performed on a Bruker AVANCE II 400 MHz instrument. Multiplicity determinations (HSQC-DEPT) and 2D spectra (COSY, HSQC, and HMBC) were obtained using standard Bruker software. Chemical shifts are expressed in ppm (δ) units using tetramethylsilane as the standard. Exact mass spectra were obtained on a Bruker microTOF-Q II mass spectrometer, equipped with an ESI source operating in positive mode. Chromatographic separations were performed by column chromatography and vacuum on silica gel 60 (0.063–0.200 mm), flash chromatography on Sepacore^®^ 10× and 50×, and preparative TLC on silica gel 60 F_254_ (0.2 mm thick) plates. The presence of compounds was revealed by an anisaldehyde reagent. Reagents used for derivatization were purchased in Sigma-Aldrich.

### Plant material

The aerial parts of *Ambrosia tenuifolia* Spreng. (Asteraceae) were collected in Río de los Sauces (−32.535932; −64.589681), Calamuchita Department, Córdoba, Argentina, in February 2019. The plant material was identified by Gloria Barboza (IMBIV-CONICET, Córdoba, Argentina), and a voucher specimen was deposited on the Museo Botánico de Córdoba (CORD 00098343), Universidad Nacional de Córdoba. The authors declare that the pertinent collection, transfer, and conservation permissions have been obtained through Research Authorization No. DRC 443 to the Instituto Multidisciplinario de Biología Vegetal, under procedure IF-2023-15771684-APN-DRC#APNAC. Furthermore, they declare that all experiments and protocols with plant species were carried out with current institutional guidelines and legislation.

### Crude extracts and major compounds

#### Extract derivatization

In a reaction flask, 579.2 mg of ATD extract (dissolved in 15 mL of toluene), *p*-TsOH (304.2 mg; 1.60 mmol, 1 eq.) was added, considering the sum of moles of psilostachyins (A, B and C) in the DCM extract. The solution was stirred for one hour at the reflux temperature of toluene (111 °C). After this time, the crude reaction was cooled and transferred to a separating funnel, 100 mL of distilled water was added, which was partitioned using DCM (5 × 50 mL). The combined organic phases were washed using distilled water (2 × 100 mL) and finally washed with saturated NaCl solution (100 mL). The solution was dried using Na_2_SO_4_ and subsequently evaporated to remove the DCM. The remaining toluene in the organic phase was evaporated with MeOH (by azeotropic mixture formation) to obtain the dry reaction crude, ATM (521.2 mg).

#### Purification of the modified ATM extract.

A 280 mg fraction of the ATM extract was purified by flash chromatography (silica gel 60-G) using Hx:EtOAc:MeOH as eluting solvents in increasing polarity ratios and 0.5% AcOH to acidify the medium, obtaining seven fractions (F1-F7). Fraction 2 (81.4 mg) was repurified on preparative TLC (silica gel 60-G with gypsum as binder) using a mobile phase of Hx:EtOAc (70:30) afforded compound **2** (23.8 mg) and **5** (58.2 mg). Fraction 3 (36.3 mg) was repurified on preparative TLC using Hx:EtOAc:AcOH (60:40:0.5) as mobile phase resulting in PsiA (11.1 mg) and compound **1** (24.7 mg). Fraction 4 (26.5 mg) was repurified with the same methodology as fraction II, afforded PsiA (10.8 mg), compound **1** (10.4 mg), and **3** (7 0.7 mg). Fraction 5 (37.6 mg) was repurified by preparative TLC using Hx:EtOAc (40:60) as mobile phase and resulted in mostly PsiA (33.5 mg) and compound **4** (4.1 mg). Finally, fraction 7 was composed by PsiC (22.5 mg) as the major compound. Fractions 1 and 6 did not show relevant content due to TLC. The structures of the compounds were determined by a combination of 1- and 2-dimensional NMR spectroscopic methods, together with the exact masses and isotopic distribution using HRMS spectrometry. All compounds were determined to be > 95% pure by ^1^H NMR spectroscopy. See supplementary information for detailed NMR data and structural elucidation.

(1*R*,5*S*)-4-((*E*)-2-carboxyvinyl)-3,5-dimethylbicyclo[3.2.2]nona-3,6-diene-6-carboxylic acid (**1**, 20%) White amorphous powder; [α]^25^_D_: + 32.1 (c 0.26, CH_2_Cl_2_).; UV (acetonitrile) λ_max_ (log ε) 278 (3.47), 211 (3.83) nm. IR (dry film) ν_max_: 2925, 2610, 1687, 1640, 1425, 1280, 1216, 755 cm^−1^. ^1^H NMR: 7.50 d (1H, J = 16.2 Hz, H-2), 7.10 d (1H, J = 4.3 Hz, H-13), 5.85 d (1H, J = 16.2 Hz, H-3), 2.71 m (1H, H-7), 2,58 brd (1H, J = 18.4 Hz, H-9a), 2,53 brd (1H, J = 18.5 Hz, H-6a), 2.05 brd (1H, J = 18.4 Hz, H-9b), 2.02 brd (1H, J = 18.5 Hz, H-6b), 1.79 s (3H, H_3_-15), 1.61 m. (2H, H_2_-8), 1.20 s (3H, H_3_-14); ^13^C NMR: 172.7 (C, C-12), 172.4 (C, C-4), 145.3 (CH, C-2), 144.5 (CH, C-13), 135.9 (C, C-5), 133.9 (C, C-1), 129.2 (C, C-11), 120.7 (CH, C-3), 38.7 (CH_2_, C-6), 37.8 (CH_2_, C-8), 36.2 (CH_2_, C-9), 33.1 (C, C-10), 30.0 (CH, C-7), 28.0 (CH_3_, C-14), 21.1 (CH_3_, C-15). HRESIMS m/z [M + Na]^+^ 285.1095 (calcd for C_15_H_18_NaO_4_^+^, 285.1097).

Methyl (1*R*,5*S*)-4-((*E*)-3-methoxy-3-oxoprop-1-en-1-yl)-3,5-dimethylbicyclo[3.2.2]nona-3,6-diene-6-carboxylate (**2**, 12%) White amorphous powder; [α]^25^_D_: −2.01 (c 0.42, Acetone); UV (acetonitrile) λ_max_ (log ε) 333 (2.43) nm, 206 (3.56) nm; IR (dry film) ν_max_: 2927, 1722, 1437, 1274 cm^−1^. ^1^H NMR: 7.37 d (1H, J = 16.1 Hz, H-2), 7.08 d (1H, J = 5.9 Hz, H-13), 5.80 d (1H, J = 16.1 Hz, H-3), 3.75 s (3H, H-1'), 2.69 m (1H, H-7), 2.50 m (1H, H-9a), 2.48 m (1H, H-6a), 2.00 m (1H, H-9b), 1.98 m (1H, H-6b), 1.74 s (3H, H_3_-15), 1.58 m (2H, H_2_-8), 1,17 s (3H, H_3_-14); ^13^C NMR: 172.4 (C, C-12), 167.5 (C, C-4), 144.6 (CH, C-13), 143.5 (CH, C-2), 133.9 (C, C-1), 129.1 (C, C-5), 128.0 (C, C-11), 121.9 (CH, C-3), 51.5 (CH_3_, C-1'), 38.3 (CH_2_, C-6), 37.5 (CH_2_, C-8), 36.3 (CH_2_, C-9), 33.0 (C, C-10), 30.1 (CH, C-7), 28.0 (CH_3_, C-14), 21.0 (CH_3_, C-15). HRESIMS *m/z* [M + Na]^+^ 299.1260 (calcd for C_16_H_20_NaO_4_^+^, 299.1254).

(3a*S*,6*S*,7*R*,8a*R*)-6-methyl-3,8-dimethyleneoctahydro-5'H-spiro[cyclohepta[b]furan-7,2'-furan]-2,5'(3H)-dione (**3**, 33%) White amorphous powder; [α]^25^_D_: −46.2 (c 0.67; acetone). UV (acetonitrile) λ_max_ (log ε) 279 (3.00) nm, IR (dry film) ν_max_: 2933, 1759, 1461, 1270, 1198, 1007, 760 cm^−1^. ^1^H NMR: 6.30 d (1H, J = 2.5 Hz, H-13a), 5.64 d (1H, J = 2.2 Hz, H-13b), 5.57 d (1H, J = 1.9 Hz, H-15a), 5.27 brd (1H, J = 8.0 Hz, H-6), 5.10 d (1H, J = 1.5 Hz, H-15b), 3.34 m (1H, H-7), 2.50 m (1H, H-3a), 2.50 m (1H, H-2a), 2.48 m (1H, H-2b), 2.26 m (1H, H-2b), 2.09 m (1H, H-10), 2.04 m (1H, H-8a), 1.60 m (1H, H-8b), 1.56 m (2H, H_2_-9), 0.95 d (3H, J = 7.3 Hz, H_3_-14); ^13^C NMR: 175.5 (C, C-4), 169.5 (C, C-12), 142.5 (C, C-5), 139.9 (C, C-11), 123.2 (CH_2_, C-13), 113.5 (CH_2_, C-15), 92.3 (C, C-1), 78.7 (CH, C-6), 43.8 (CH, C-7), 39.6 (CH, C-10), 33.9 (CH_2_, C-2), 30.6 (CH_2_, C-8), 28.4 (CH_2_, C-3), 26.9 (CH_2_, C-9), 14.4 (CH_3_, C-14). HRESIMS *m/z* [M + Na]^+^ 285.1099 (calcd for C_15_H_18_NaO_4_^+^, 285.1097).

(3a*S*,6*S*,7*S*,8a*R*)-6-methyl-3,8-dimethyleneoctahydro-5'H-spiro[cyclohepta[b]furan-7,2'-furan]-2,5'(3H)-dione (**4**, 21%) White amorphous powder; [α]^25^_D_: + 28.2 (c 0.39; acetone). UV (acetonitrile) λ_max_ (log ε) 279 (3.32) nm, IR (dry film) ν_max_: 2927, 1762, 1459, 1271, 1194, 1005, 758 cm^-1^. ^1^H NMR: 6.34 d (1H, J = 2.9 Hz, H-13a), 5.62 d (1H, J = 2.4 Hz, H-13b), 5,36 brs (2H, H_2_-15), 5.15 brd (1H, J = 8.6 Hz, H-6), 3.24 m (1H, H-7), 2.59 m (2H, H_2_-3), 2.23 m (2H, H_2_-2), 1.72 m (2H, H_2_-9), 1.71 m (2H, H_2_-8), 0.98 d (3H, J = 6.6 Hz, H_3_-14); ^13^C RMN: 176.2 (C, C-4), 169.4 (C, C-12), 144.2 (C, C-5), 138.2 (C, C-11), 123.9 (CH_2_, C-13), 112.3 (CH_2_, C-15), 88.6 (C, C-1), 77.4 (CH, C-6), 41.8 (CH, C-7), 38.5 (CH, C-10), 31.7 (CH_2_, C-2), 29.8 (CH_2_, C-9), 27.9 (CH_2_, C-3), 27.4 (CH_2_, C-8), 14.1 (CH_3_, C-14). HRESIMS m/z [M + Na]^+^ 285.1099 (calcd for C_15_H_18_NaO_4_^+^, 285.1097).

3-((3a*S*,6*S*,7*R*,8a*R*)-6,7-dimethyl-3-methylene-2,8-dioxooctahydro-2H-cyclohepta[b]furan-7-yl)propanoic acid (**6**, 9%) White amorphous powder; [α]^25^_D_: + 110.4 (c 0.22; acetone). UV (acetonitrile) λ_max_ (log ε) 283 (2.43) nm. IR (dry film) ν_max_: 3505, 2932, 1765, 1703, 1459, 1260, 1174, 982, 957 cm^-1^. ^1^H NMR: 6.11 d (1H, J = 2.9 Hz, H-13a), 5.48 d (1H, J = 2.4 Hz, H-13b), 4.79 brd (1H, J = 7.1 Hz, H-6), 3.22 m (1H, H-7), 2.80 brs (2H, H_2_-2), 2.43 brs (2H, H_2_-3), 2.01 m (1H, H-10), 1.78 m (2H, H_2_-8), 1.35 m (2H, H_2_-9), 1.12 s (3H, H_3_-15), 0.90 d (3H, J = 7.0 Hz, H_3_-14); ^13^C NMR: 214.4 (C, C-5), 180.1 (C, C-4), 170.7 (C, C-12), 139.8 (C, C-11), 118.6 (CH_2_, C-13), 80.7 (CH, C-6), 54.6 (C, C-1), 38.0 (CH, C-7), 34.2 (CH_2_, C-3), 33.5 (CH, C-10), 29.9 (CH_2_, C-2), 25.2 (CH_2_, C-9), 22.6 (CH_2_, C-8), 15.5 (CH_3_, C-14), 13.1 (CH_3_, C-15). HRESIMS *m/z* [M + Na]^+^ 303.1208 (calcd for C_15_H_20_NaO_5_^+^, 303.1203).

2-((1*S*,2*S*)-2-methoxy-4-(3-methoxy-3-oxopropyl)-3,5-dimethylcyclohepta-3,5-dien-1-yl)acrylic acid (**7**, 5%) White amorphous powder; [α]^25^_D_: −12.2 (c 0.11, acetone). UV (acetonitrile) λ_max_ (log ε) 202 (3.57) nm. IR (dry film) ν_max_: 3454, 2925, 2853, 1736, 1629, 1442, 1377, 1260, 1170, 1105 cm^−1^. ^1^H NMR: 6.21 brs (1H, H-13a), 5.91 t (1H, J = 6,7 Hz, H-9), 5.84 brs (1H, H-13b), 3.84 d (1H, J = 11.3 Hz, H-6), 3.66 s (3H, OCH_3_-2'), 3.26 s (3H, OCH_3_-1'), 3.24 m (1H, H-7), 2.70 m (2H, H_2_-2), 2.30 m (2H, H_2_-3), 2.23 m (H, H-8a), 1.88 s (3H, H_3_-15), 1.86 m (1H, H-8b), 1.82 s (3H, H_3_-14); ^13^C NMR: 173.6 (C, C-4), 169.7 (C, C-12), 144.8 (C, C-11), 138.3 (C, C-10), 135.7 (C, C-1), 134.3 (C, C-5), 128.0 (CH, C-9), 123.8 (CH_2_, C-13), 87.0 (CH, C-6), 58.6 (CH_3_, C-1′), 53.8 (CH, C-7), 51.3 (CH_3_, C-2′), 32.8 (CH_2_, C-3), 29.9 (CH_2_, C-8), 24.5 (CH_2_, C-2), 20.2 (CH_3_, C-14), 11.6 (CH_3_, C-15). HRESIMS m/z [M + Na]^+^ 303.1208 (calcd for C_15_H_20_NaO_5_^+^, 303.1203).

Methyl 2-((1*S*,2*R*)-2-methoxy-4-(3-methoxy-3-oxopropyl)-3,5-dimethylcyclohepta-3,5-dien-1-yl)acrylate (**8**, 6%) White amorphous powder; [α]^25^_D_: −22.3 (c 0.13, acetone). UV (acetonitrile) λ_max_ (log ε) 202 (3.63) nm. IR (dry film) ν_max_: 2954, 2919, 2850, 1731, 1438, 1261, 1079 cm^−1^. ^1^H NMR: 6.19 brs (1H, H-13a), 5.86 t (1H, J = 6.5 Hz, H-9), 5.64 brs (1H, H-13b), 3.91 m (1H, H-7), 3.90 m (1H, H-6), 3.74 s (3H, H_3_-3'), 3.66 s (3H, H_3_-1'), 3,20 s (3H, H_3_-2'), 2.68 m (1H, H-2a), 2.43 m (1H, H-2b), 2.35 m (1H, H-3a), 2.33 m (1H, H-3b), 1.93 m (1H, H-8a), 1.86 m (1H, H-8b), 1,76 s (3H, H_3_-14), 1.77 s (3H, H_3_-15). ^13^C NMR: 174.0 (C, C-4), 168.7 (C, C-12), 148.9 (C, C-11), 138.2 (C, C-10), 136.0 (C, C-1), 133.9 (C, C-5), 126.8 (CH, C-9), 122.7 (CH_2_, C-13), 83.7 (CH, C-6), 57.9 (CH_3_, C-2'), 51,7 (CH_3_, C-3'), 51.5 (CH, C-7), 51,3 (CH_3_, C-1'), 33.3 (CH_2_, C-3), 28.7 (CH_2_, C-8), 24.3 (CH_2_, C-2), 20.4 (CH_3_, C-14), 13.6 (CH_3_, C-15). HRESIMS *m/z* [M + Na]^+^ 346.1552 (calcd for C_18_H_26_NaO_5_^+^, 346.1751).

Methyl (2*S*,5*S*)-2-methoxy-4-(3-methoxy-3-oxopropyl)-3,5-dimethylbicyclo[3.2.2]nona-3,6-diene-6-carboxylate (**9**, 9%) White amorphous powder; [α]^25^_D_: −10.2 (c 0.15, acetone). UV (acetonitrile) λ_max_ (log ε) 202 (3.14) nm. IR (dry film) ν_max_: 2931, 1737, 1451, 1382, 1269 cm^−1^. ^1^H NMR: 6.90 d (1H, J = 4.8 Hz, H-13), 3.71 s (3H, H-3'), 3.67 s (3H, H-1'), 3.44 s (3H, H-2'), 3.19 s (1H, H-6), 2.77 m (1H, H-7), 2.38 m (3H, H_2_-3 and H-2a), 2.33 m (1H, H-9a), 2.27 m (1H, H-2b), 1.94 brd (1H, J = 18.1 Hz, H-9b), 1.82 brd (1H, J = 11.5 Hz, H-8a), 1.70 s (3H, H_3_-15), 1.30 m (1H, H-8b), 1.17 s (3H, H_3_-14); ^13^C NMR: 173.8 (C, C-4), 167.9 (C, C-12), 139.5 (CH, C-13), 138.5 (C, C-1), 131.4 (C, C-11), 127.6 (C, C-5), 82.0 (CH, C-6), 57.9 (CH_3_, C-2'), 51.5 (CH_3_, C-3'), 51.5 (CH_3_, C-1'), 35.6 (C, C-10), 35.4 (CH_2_, C-9), 34.0 (CH_2_, C-3), 33.2 (CH, C-7), 32.9 (CH_2_, C-8), 26.8 (CH_3_, C-14), 24.0 (CH_2_, C-2), 17.4 (CH_3_, C-15). HRESIMS *m/z* [M + Na]^+^ 345.1630 (calcd for C_18_H_26_NaO_5_^+^, 345.1672).

Methyl 3-((3a*S*,6*S*,7*S*,8a*R*)-6-methyl-3,8-dimethylene-2-oxooctahydro-2H-cyclohepta[b]furan-7-yl)propanoate (**11**, 50%) White amorphous powder; [α]^25^_D_: + 3.6 (c 0.35, Acetone).UV (acetonitrile) λ_max_ (log ε) 284 (2.71) nm. IR (dry film) ν_max_: 3398, 2918, 2850, 1734, 1460, 1250, 801 cm^-1^.^1^H NMR: 6.23 d (1H, J = 2.8 Hz, H-13a), 5.52 d (1H, J = 2.5 Hz, H-13b), 5.33 d (1H, J = 1.8 Hz, H-15a), 4.86 m (1H, H-15b), 4.86 m (1H, H-6), 3.11 m (1H, H-7), 2.40 m (1H, H-3a), 2.34 m (1H, H-3b), 2.01 m (1H, H-1), 1,90 m (H, H-9a), 1.89 m (1H, H-10), 1.71 m (1H, H-9b), 1,56 m (2H, H_2_-8), 1.52 m (2H, H_2_-2), 0,77 d (3H, J = 7.6 Hz, H-14); ^13^C NMR: 178.8 (C, C-4), 170.2 (C, C-12), 142.3 (C, C-5), 139.5 (C, C-11), 123.0 (CH_2_, C-13), 111.7 (CH_2_, C-15), 82.4 (CH, C-6), 44.3 (CH, C-1), 43.8 (CH, C-7), 34.9 (CH_2_, C-8), 33.6 (CH, C-10), 31.8 (CH_2_, C-3), 26.3 (CH_2_, C-2), 27.8 (CH_2_, C-9), 13.0 (CH_3_, C-14). HRESIMS m/z [M + Na]^+^ 285.1095 (calcd for C_15_H_18_NaO_4_^+^, 285.1097).

### Anti-glioblastoma activity

#### Cell line culture

T98G cells were isolated from the brain of a glioblastoma multiforme patient (ATCC, cat. No. CRl-1690, RRUD: CVCL-0556, Manassas, VA, USA). HEK 293 cells are derived from human embryonic kidney cells (ATCC Cat# CRL-1573, RRID: CVCL_0045). Both cell lines were grown in Dulbecco’s modified Eagle’s medium (DMEM, Invitrogen) supplemented with 10% fetal serum bovine serum (FBS) at 37 °C according to reference ^36^.

#### MTT assay for in vitro evaluation of anti-glioblastoma activity

3 × 10^3^ cells were seeded in a 96-well plate and were allowed to attach overnight at 37ºC. T98G cells were treated in duplicate or triplicate with different concentrations of the crude/chemically modified extract or pure compounds. The stock solutions were dissolved in DMSO at the final concentration of 160 mg/ml and 5 or 80 mM for the crude/chemically modified extracts and pure compounds, respectively. After 24 h, 100 μL of DMEM supplemented with 10% of FBS and 10% of 3-(4,5-dimethylthiazol-2-yl)-2,5-diphenyltetrazolium bromide (MTT) reagent (5 mg/mL; Sigma) were added to each well, and plates were further incubated for 2 h at 37 °C as described in references ^35,36^. Then, 100 μL of DMSO:isopropanol (1:1, v/v) were added to each well followed by incubation for a few minutes at room temperature protected from light. Absorbance was analyzed at 570 nm with a reference at 650 nm in an Epoch Microplate Spectrophotometer. Cells incubated with an equivalent DMSO concentration were considered 100% of viability. The equation “log(inhibitor) vs response—variable slope (four parameters) of the GraphPad Prism software was used to calculate the IC_50_ (half maximal inhibitory concentration) value.

To evaluate the standard treatment, T98G cultures were treated with temozolomide (5 to 1400 μM). After 72 h, the MTT assay was used to determined cell viability and the IC_50_ value was calculated as describe above.

#### Temporal susceptibility and cell viability determination

T98G cells were seeded at the density of 3 × 10^3^ in a 96-well plate and incubated overnight at 37ºC. Cultured cells were synchronized with dexamethasone (100 nM, 1 h at 37 °C) according to reference ^36^ and then treated with (**3**) (13.6 μM) or DMSO at different times post-synchronization for 24 h. After incubation, cell viability was measured by MTT assay as described above. Cells incubated with DMSO were considered as 100% of viability.

### Chemoinformatics analysis

#### Molecular modeling and deployment of 3D structures of compound libraries

The compound library, “Psi.D.”, comprises psilotachyins (PsiA, PsiB and PsiC) and their derivatives (compounds **1–11**). Three additional libraries of compounds with antiglioblastoma properties were created using the information obtained from the ChEMBL ^59^ database (36 approved drugs with IC_50_ reported against the T98G line, named “anti-T98G”), 43 compounds undergoing anti-glioblastoma studies in clinical phase, referred to as “Cli. Ph.”; and a final library composed of 28 Natural antiglioblastoma products, denoted as “NP”. All four libraries were prepared using MOE (Molecular Operating Environment)^60^. Partial charges were assigned to the atoms and their geometries were optimized with the MMFF94x force field. Finally, the majority species of each library were determined at physiological pH (7.4) for further analysis. The composition of these libraries and the bibliographic references used for its construction are detailed in Table S5 of the supplementary information.

#### Generation of chemical space through principal component analysis of molecular descriptors

The MOE program allowed the calculation of 435 molecular descriptors for all libraries^61^. With the pIC50 information obtained for the anti-T98G library, a contingency analysis calculation was carried out with the QuaSAR-Contingency tool available in the program. It was determined that 83 molecular descriptors satisfied the cutoff values of four parameters defined by MOE^62^; therefore, they were selected as having a correlation with the reported IC_50_ values. The values of these parameters and the details of the chosen descriptors are available in the supplementary information (Table S3).

The Principal Components Analysis tool of the MOE program was employed, selecting the 83 molecular descriptors with the Autoscale Fields option activated, to generate two principal components (PC) that together describe 77.5% of the variance (see Table S4 in supplementary information). The first two PCs were plotted using Graphpad Prism 8.0 to position all the libraries in a two-dimensional chemical space and used to perform a cluster analysis with the QuaSAR-Cluster tool using a smoothing value of 15.0. This analysis grouped all the molecules into five clusters and two monoclusters, which can be seen in Table S5 and Fig. S16.

#### Structural comparison and druglikeness analysis

All the SMILES of the compounds forming the Psi D. library were obtained using the MOE program, then loaded and analyzed with the similarity tool of the ChemMineTools platform (http://chemmine.ucr.edu/similarity) and SwissADME predictions (http://www.swissadme.ch/). The Tanimoto coefficients obtained as atom pair descriptors were used to create a symmetrical histogram of comparing all the analyzed molecules and colored according to similarity values.

### Supplementary Information

Supplementary Information.

## Data Availability

Data is provided within the manuscript or supplementary information files. Indeed, all data and materials are available at the Department of Organic Chemistry, Universidad Nacional de Córdoba being able to contact the corresponding author to request the data of this study.
