# Supplementary material for: Enhancing structural diversity through chemical engineering of Ambrosia tenuifolia extract for novel anti-glioblastoma compounds
Source: Sci Rep. 2024 Jun 20;14:14229. doi: 10.1038/s41598-024-63639-y (PMC11190268; doi:10.1038/s41598-024-63639-y)

Structural elucidation of undescribed compounds

For compound (**1**), the ^{1}H NMR spectrum showed three vinylic proton signals at δ_H 7.50 d (16.2 Hz), 5.85 d (16.2 Hz), and 7.10 d (4.3 Hz). The first two correspond to a trans double bond only possible in a freely rotating aliphatic carbon chain. The correlation of these vinyl protons with a carbonyl group at δ_C at 172.4 in the HMBC spectrum and the high chemical shift of H-2 confirmed the opening of the lactone of the A ring of PsiB and the location of the double bond in the C2-C3 position in a conjugate system. The signal at δ_H 7.10, corresponding to a vinylic proton of a trisubstituted double bond, showed coupling in the COSY spectrum with a methine at δ_H 2.71 assigned to H-7 and correlations with carbons at δ_C 30.0 (C-7), δ_C 36.2 (C-8), δ_C 37.8 (C-9), and δ_C 172.7 (C-12) in the HMBC spectrum. On the other hand, the methyl group at δ_H 1.20, assigned to H_3-14, showed correlations in the HMBC spectrum with the signals at δ_C 133.9 (C-1), a quaternary carbon assigned to C-10 (δ_C 33.0), C-8 (δ 37.8), and C-9 (36.2), while the methyl group at δ 1.79, assigned to H_3-15, showed correlations with the signals corresponding to C-1, C-5 (δ 135.9), and C-6 (δ 38.7). All these observations allow us to infer an arrangement with a C10-C11 bond.

The NMR spectra of compound (**2**) were closely related to those of (**1**), differing only by the singlet signal at δ_H 3.75 corresponding to a methoxy group. The analysis of the HMBC spectrum allowed us to establish the esterification of the carboxylic group at the C-4 position by the crossing peak between the signal at δ_H 3.75 and the carbon at δ_C 167.5 assigned to C-4.

Regarding the two epimeric derivatives, the ^{1}H-NMR spectrum of (**3**) showed signals corresponding to two exocyclic double bonds, one corresponding to the C11-C13 double bond (δ_H 6.30 and δ_H 5.64) and two other signals at δ_H 5.57 and δ_H 5,10. The allylic and homoallylic coupling between the signal at δ_H 5.57 and H-6 (δ_H 5.27 brd) and between the signal at δ_H 5.57 and H-7 (δ_H 3.34 m), respectively, allows us to infer the location of the unsaturation in the C5-C15 positions. This product was already obtained by Mabry et al. by dehydration of PsiA with a mixture of acetic acid and sulfuric acid (90:10). The NOESY spectrum was consistent with the configuration of C-1 given the coupling observed between H-15b and H-2a (δ_H 2.50).

The ^{1}H-NMR spectrum of compound (**4**) showed signals corresponding to the vinyl protons of the C11-C13 exocyclic double bond at δ_H 6.34 and δ_H 5.62 and a signal that integrates for two vinyl protons at δ_H 5.36. The chemical shift and the multiplicity of the signal assigned to H-6, δ_H 5.15 brd (8.6 Hz), allow the unsaturation to be established in the same position as in compound (**3**) The detailed analysis of the NMR spectra in 1 and 2 dimensions allow us to confirm that no other modifications have occurred in this derivative in relation to the substrate. The most relevant change observed between compounds (**3**) and (**4**) suggest an inversion in the configuration of C-1. In compound (**3**), the chemical shifts of the H-15a and H-15b protons differ by 0.47 ppm since the oxygen atom of the ester function of ring A is very close to one of the vinyl protons, while in compound (**4**) the protons H_2-15 have the same chemical shift. As can be seen in the Fig. 3, for compound (**4**) the ester function of ring A has an opposite arrangement to the C5-C15 double bond. The C-1 configuration was confirmed through the couplings observed in the NOESY spectrum, only possible for the proposed configuration; these are the crossing peaks between the signal of H_2-2 (δ_H 2.33 m) with H-6.

In the derivatization of the pure compounds, the ^{1}H NMR spectrum of compound (**6**) did not show signals markedly different from those corresponding to PsiA, however, the ^{13}C NMR spectrum showed a signal at δ_C 214.4 typical of a saturated ketone. In the HMBC spectrum, a crossing peak was observed between the signal at δ_C 214.4 and the signal at δ_H 4.79 assigned to H-6 and at δ_H 1.12 assigned to H_3-15 methyl group. This observation suggested the presence of the ketone function at C-5 and a transposition of the H_3-15 group from the C-5 position to C-1 after opening the lactone of the A ring. This structural arrangement was confirmed from the variation of the chemical shift of C-1 from 79.4 ppm for PsiA, typical of a carbinolic carbon, to 54.6 ppm and the correlations observed in the HMBC spectrum between the signal assigned to C-1 and the proton signals of H_3-14 at δ_H 0.90, H_3-15 and H-10 at δ_H 2.01. The configuration of C-1 was determined from the crossing peak observed in the NOESY spectrum between H-6 and H_2-3 (δ_H 2.80), which is only possible with H_3-15 with a β orientation.

The ^{1}H NMR spectrum of (**7**) showed two signals that integrate for three protons each characteristic of two methoxy groups at δ_H 3.26 and δ_H 3.66 and three signals of vinyl protons: the typical signals of the C11-C13 exocyclic double bond (13a at δ_H 6.21 and 13b at δ_H 5.87) and a signal at δ_H 5.91 corresponding to a trisubstituted double bond. The signal at 3.26 ppm showed correlation in the HMBC spectrum with the signal at δ_C

87.0 assigned to C-6 suggesting the opening of the lactone of the C ring and subsequent methoxylation of the free hydroxyl group at C-6. The analysis of the HMBC spectrum allows establishing the presence of two signals corresponding to carbonyl groups at δ_C 173.6 and δ_C 169.7 and six vinylic carbons; the signals at δ_C 144.8 and δ_C 123.8 assigned to positions C-11 and C-13, a signal from a methine at δ_C 128.0 and three quaternary carbon signals at δ_C 134.3, δ_C 135.7 and δ_C 138.3. The signal of the carbonyl group at 169.7 ppm showed cross peak in the HMBC spectrum with the H_2-13 signals, which allows it to be assigned to the C-12 position, and the signal at 173.6 ppm showed a cross peak with the methoxy group at δ_H 3.66 suggesting that the carboxyl group at 169.7 ppm is free and that the opening of the lactone of the A ring and subsequent methoxylation in the C-4 position has occurred. A detailed analysis of the COSY and HMBC spectra allowed us to locate the tetrasubstituted double bond in the C1-C5 position and the trisubstituted double bond in the C9-C10 position. Finally, the orientation of the methoxy group at C-6 is α since the H-6 signal is a doublet with a coupling constant of 11 Hz, only possible with a trans di-axial arrangement with H-7.

Continuing with the derivatives obtained, the NMR spectra indicate that derivative (**8**) is almost identical to compound (**7**). The only difference is the presence of a third signal at δ_H 3.74 corresponding to an OCH_3 group in the ^{1}H-NMR spectrum at the C-12 position. The localization of the OCH_3 group was confirmed by the cross peak observed in the HMBC spectrum between the signal at δ_H 3.74 and the signal at δ_C 168.7 corresponding to C-12. Unlike compound (**7**), the orientation of the methoxy group at C-6 in (**8**) would be β. This proposal was based on two observations; although the signals of the H-6 and H-7 protons are overlapped and the coupling constants cannot be measured in the ^{1}H NMR spectrum, the approximate measurement of the H-6/H-7 coupling constant in the H-6/C-6 cross peak in the HSQC spectrum was around 6.5 Hz; on the other hand, an appreciable variation of the chemical shift of H-7, from δ_H 3.24 for (**7**) to δ_H 3.91 for (**8**) was observed, suggesting the inversion of the C-6 configuration. The NOESY spectrum showed no useful correlations.

In the case of compound (**9**) the ^{1}H NMR spectrum showed a signal corresponding to a vinyl proton at δ_H 6.90, a signal from a carbinolic proton at δ_H 3.19, and five methyl signals of which three correspond to methoxy groups at δ_H 3.44, δ_H 3.67, and δ_H 3.71. The analysis of the HMBC spectrum allowed establishing the presence of two signals of carbonyl groups at δ_C 173.8 and δ_C 167.9 which showed correlations in the HMBC

spectrum with the methyl groups at δ_H 3.67 and δ_H 3.71. These correlations suggested the opening of the two lactones of PsiB, rings A and C. The signal corresponding to the third methoxy group shows correlation in the HMBC spectrum with the carbinolic proton assigned to the H-6 position. From the HSQC and HMBC spectra, the presence of two double bonds, one tetra-substituted and one tri-substituted, was inferred. The detailed analysis of the 2D spectra allowed us to locate the tetrasubstituted double bond in the C1-C5 position. On the other hand, the vinyl proton signal showed a strong scalar coupling with the signal assigned to H-7 (δ_H 2.77) in the COSY spectrum. Likewise, the HMBC spectrum showed key correlations between the methyl group at δ_H 1.17 (H_3-14) with a quaternary carbon at δ_C 35.6 assigned to C-10 and the C-7 at δ_C 32.8 and between the H_2-9 (δ_H at 2.33 and 1.94) and the signal at δ_C 131.4 assigned to C-11. All these considerations allowed us to establish the formation of a C-C bond between positions C10-C11. Finally, the α orientation for the methoxy group at C-6 was established by the NOE observed between the H- 6 and H-13.

Finally, the compound (**11**) was not previously reported. In this case, the chemical shifts of protons and carbons are very similar to those of compounds (**3**) and (**4**). The differences involve the positions of ring A. On the one hand, the spiranic C-1 signal is absent and a methine is deduced in that position (δ_H 2.09, δ_C 44.2) through allylic coupling with H_2-15 (δ_H 5.33 and δ_H 4.86). This observation implies the opening of the lactone of the A ring confirmed by the molecular formula obtained by mass spectrometry and a dehydration at the C5-C15 position.

In all cases, HRMS spectra agree with the proposed structures and the insertion of OMe residues comes from the processing of the sample during the workup with MeOH.

Scheme S1: Plausible ring distortion synthetic pathway of PsiB to produce compound (**1**). (a)(c) protonation and opening of the lactones; (b) loss of allylic proton to produce a conjugated diene; (d) loss of allylic proton to produce a conjugated triene; (e) (f) keto-enol tautomerism to produce extended conjugation; (g) (i) Diels–Alder reactions; (h) Retro-cycloaddition [2+2]; (j) protonation of the carbonyl group; (k) carbocation rearrangements by keto-enol tautomerism; (l) [1,3] hydride migration; (m) deprotonation.

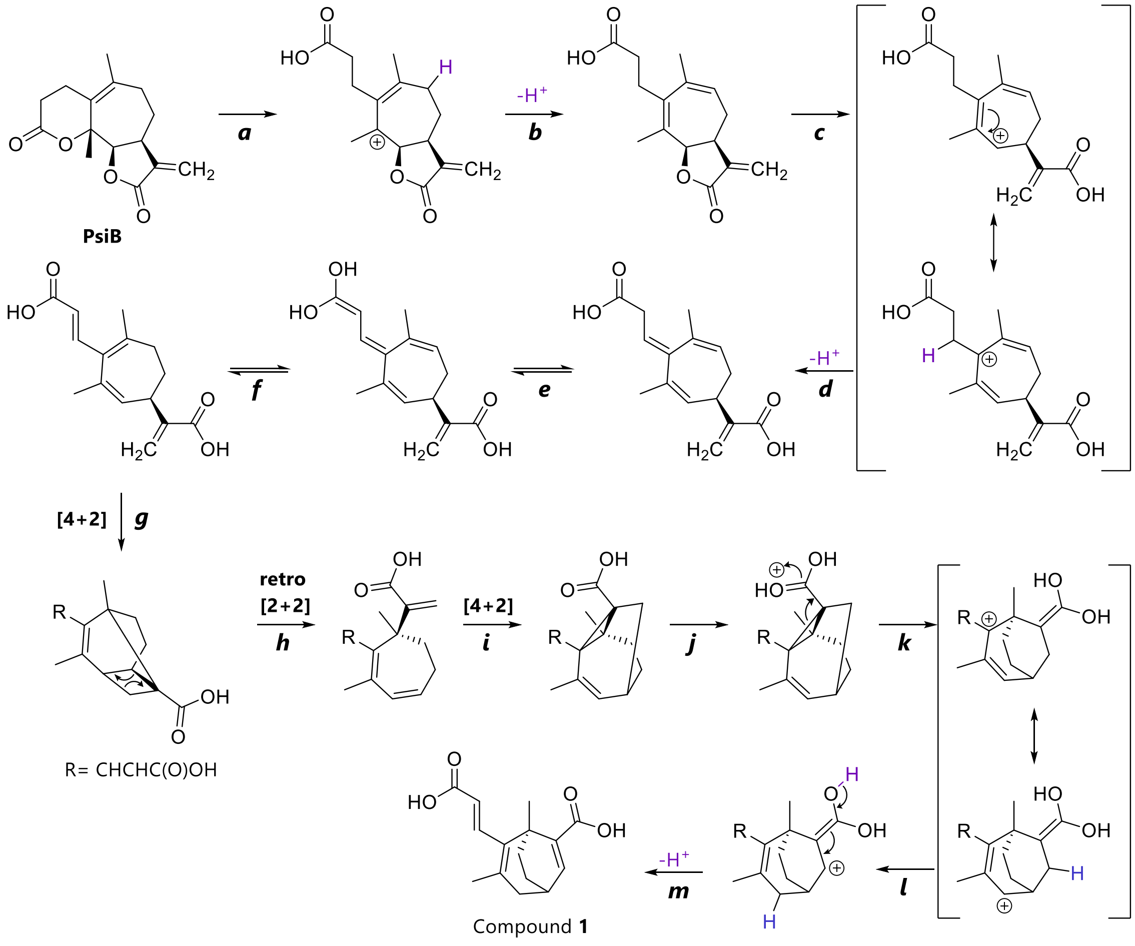

Scheme S2: Plausible ring distortion mechanistic pathway of PsiA to produce compound (**6**) a) spirolactone hydrolysis; (b) the formation of an oxygen-carbon double bond which in concert produces a 1,2-migration of methyl; (c) deprotonation of the ketone carbonyl.

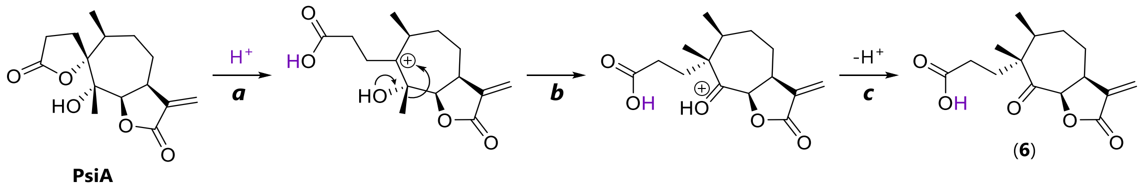

Scheme S3: Plausible ring distortion mechanistic pathway of PsiB to produce compound (**9**) (a) (c) transesterifications; (b) loss of allylic proton to produce a conjugated diene; (d) nucleophilic attack by methanol; (e) Diels–Alder reaction; (f) protonation of the carbonyl group; (g) (i) carbocation rearrangements via keto-enol tautomerism; (h) nucleophilic attack of the enol; (j) 1,3-migration of alkyl chain; (k) 1,4 hydride migration; (l) deprotonation of the carbonyl group.

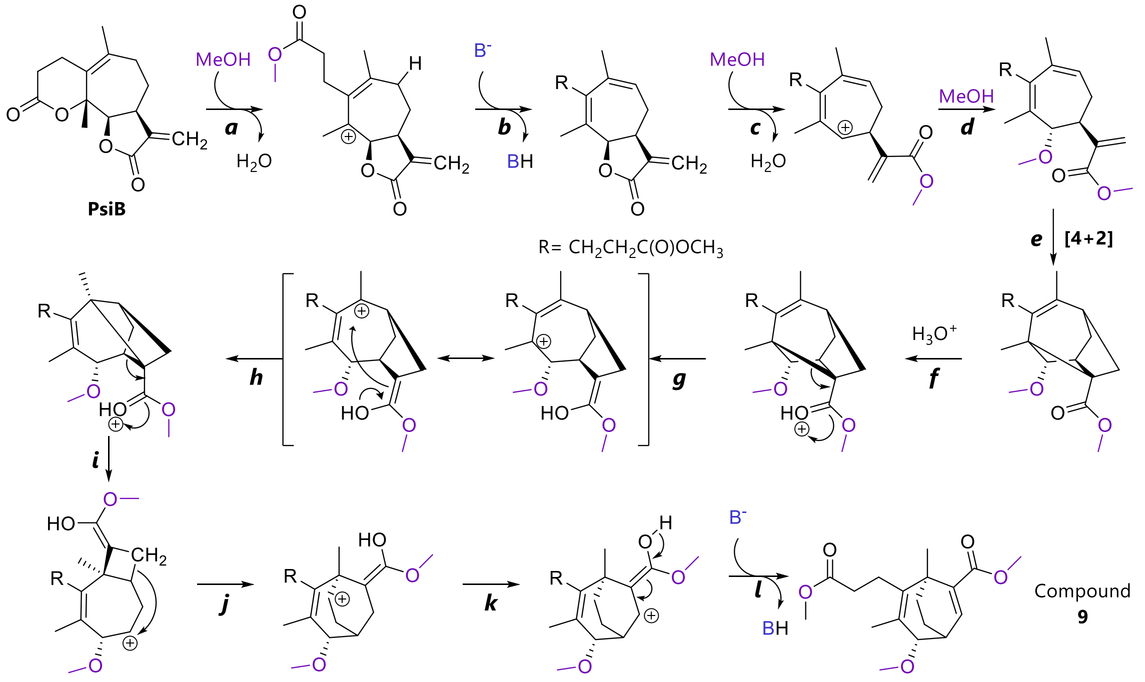

Scheme S4: Global outline of the isolation process workflow using bioguided fractionation.

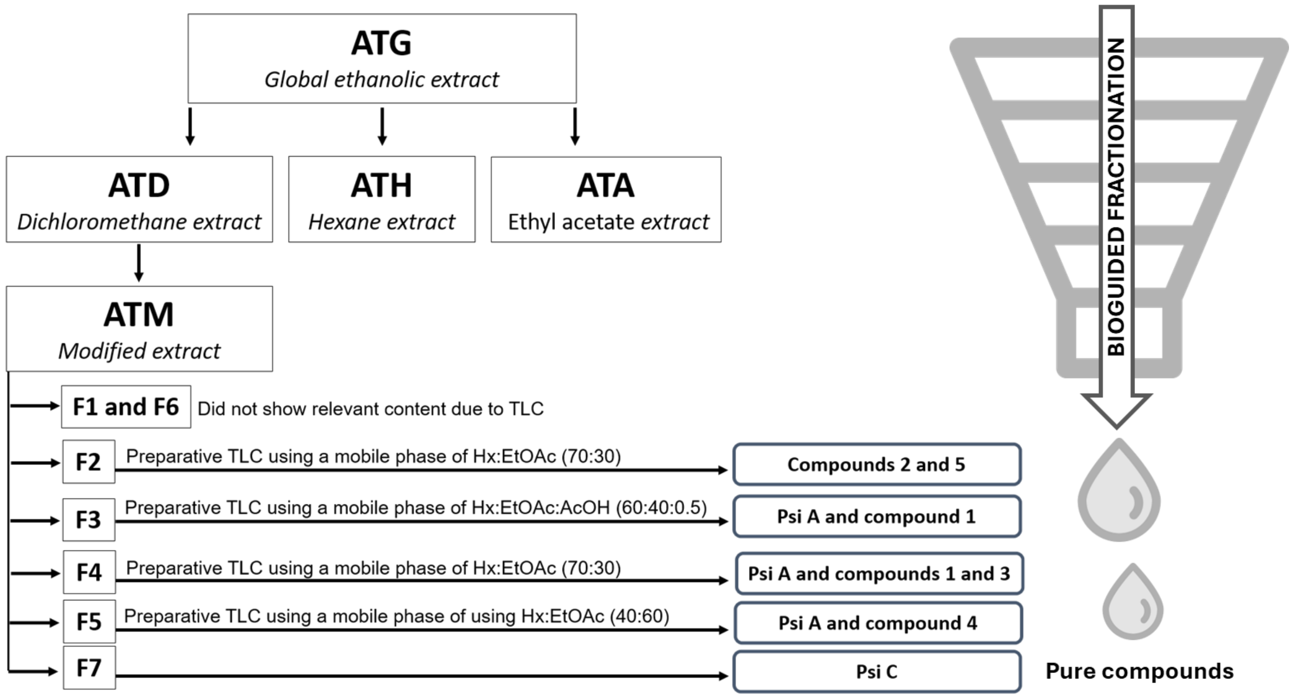

Figure S1: Chemically modified extracts were incubated with T98G for 24 h with concentrations from 10 to 160 µg/mL. A reduction greater than 50% in cell viability assayed by the MTT experiment was observed with a concentration higher than 40 µg/mL for hexane extract (ATH) and extract derivatized in an acid medium with *p*-toluenesulfonic acid (ATM) and 80 µg/mL for ethyl acetate extract (ATA). A concentration greater than 40 µg/mL of the fractions F2, F3, F4, and (**4**) was needed to reduce 50% of cell viability while the fraction F5 showed a reduction of more than 25% of cell viability when T98G cultures were treated with 40 µg/mL of this fraction.

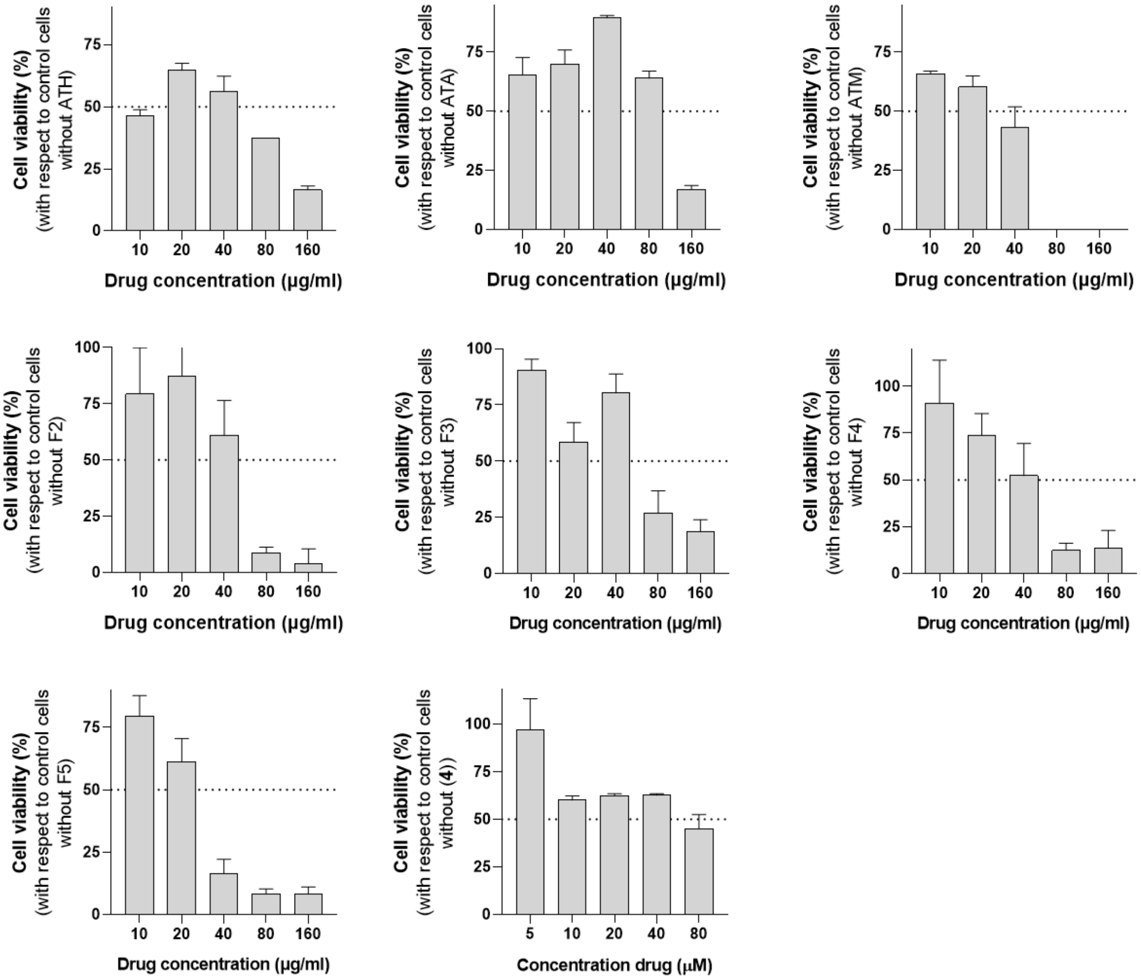

Figure S2. T98G cells were treated with the compounds (**1**), (**11**), (**2**), and (**5**) in a range of concentrations from 2.5 to 80 μM for 24 h. Results analyzed by MTT assay revealed a percentage of cell viability higher than 50% even still in concentrations of 40 o 80 μM. Results from MTT assay showed an IC_{50} of ~39 μM when HEK 293 cultures were treated with (**3**) for 24 h.

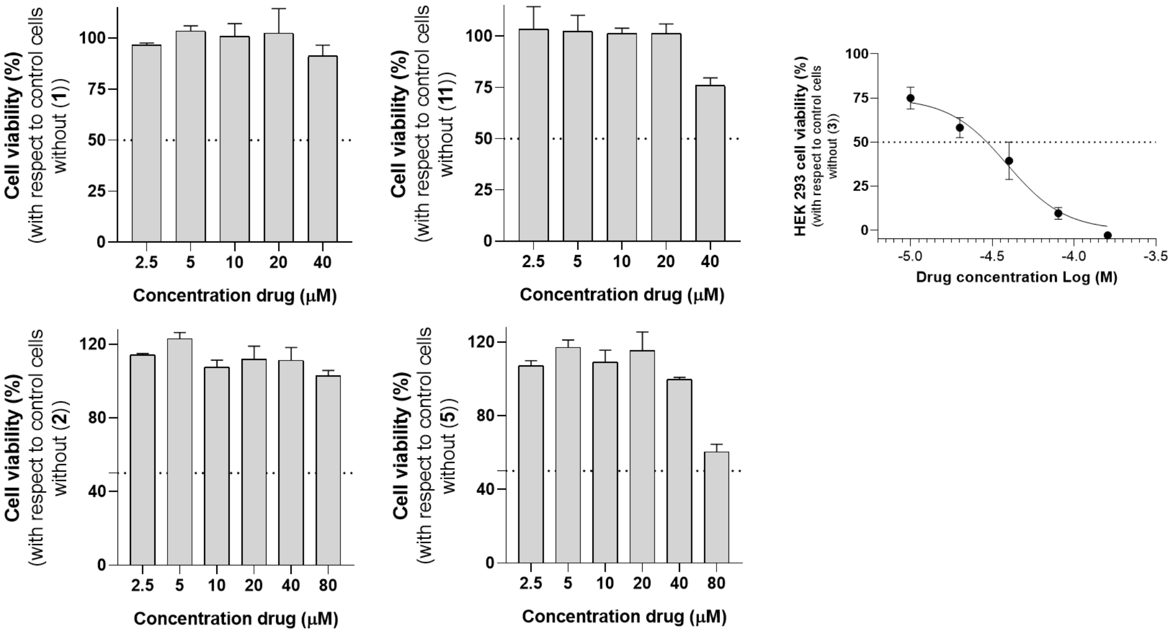

Figure S3-1. ^{1}H NMR spectrum of compound **1** ($CDCl_3$, 400.13 MHz).

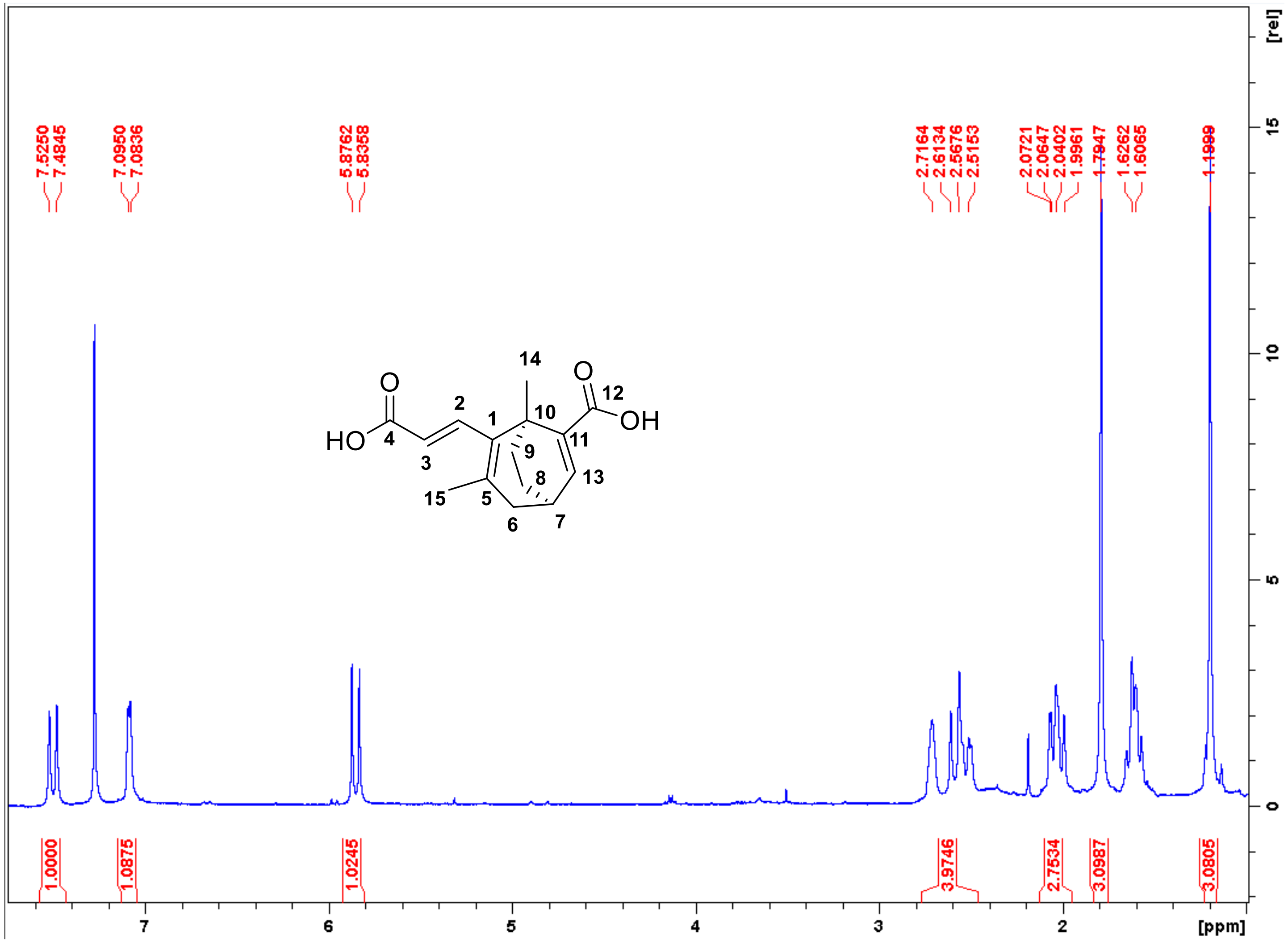

Figure S3-2. ^{13}C NMR spectrum of compound **1** ($CDCl_3$, 100.03 MHz).

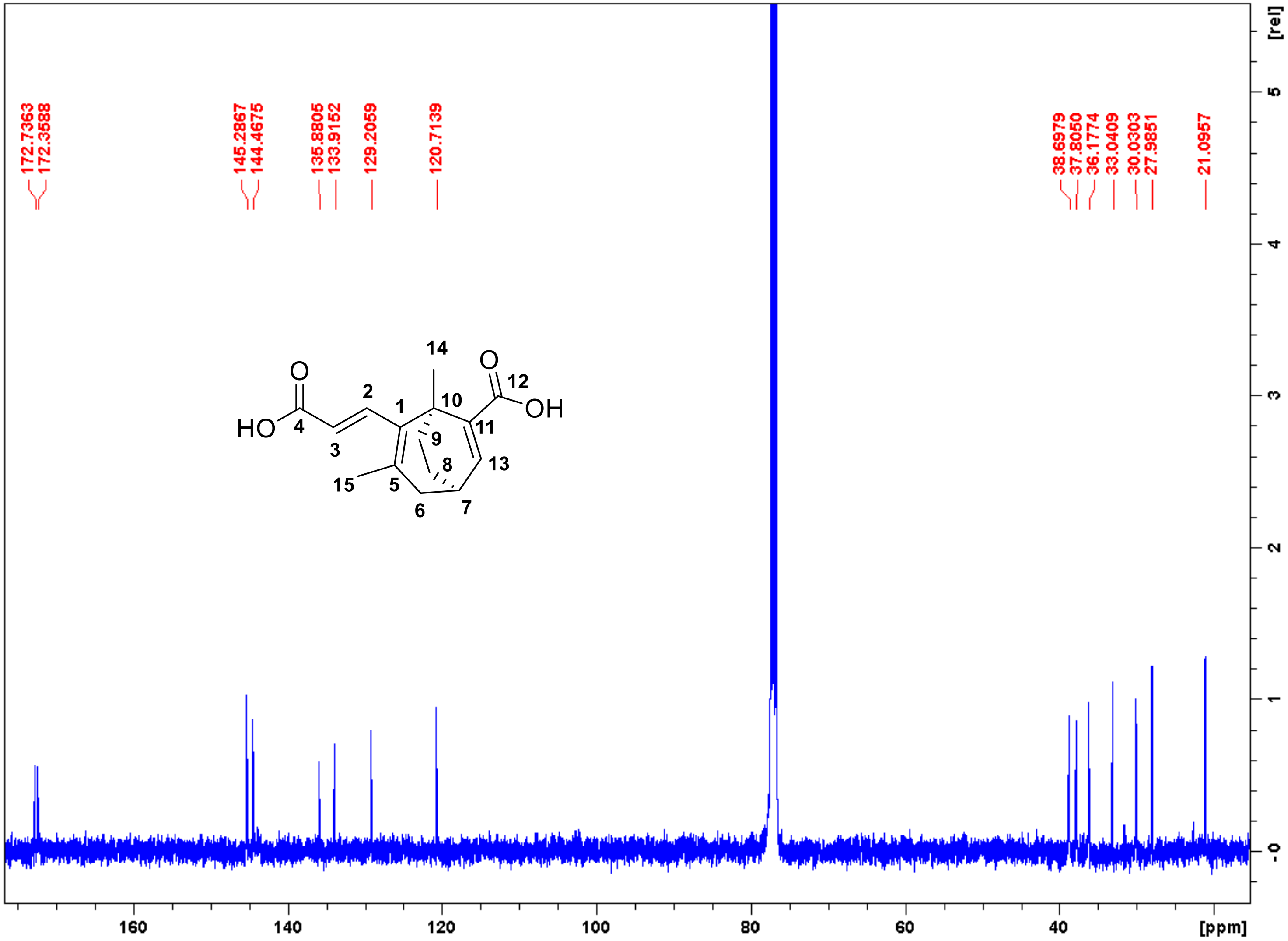

Figure S3-3. COSY spectrum of compound **1** ($CDCl_3$, 400.13 MHz).

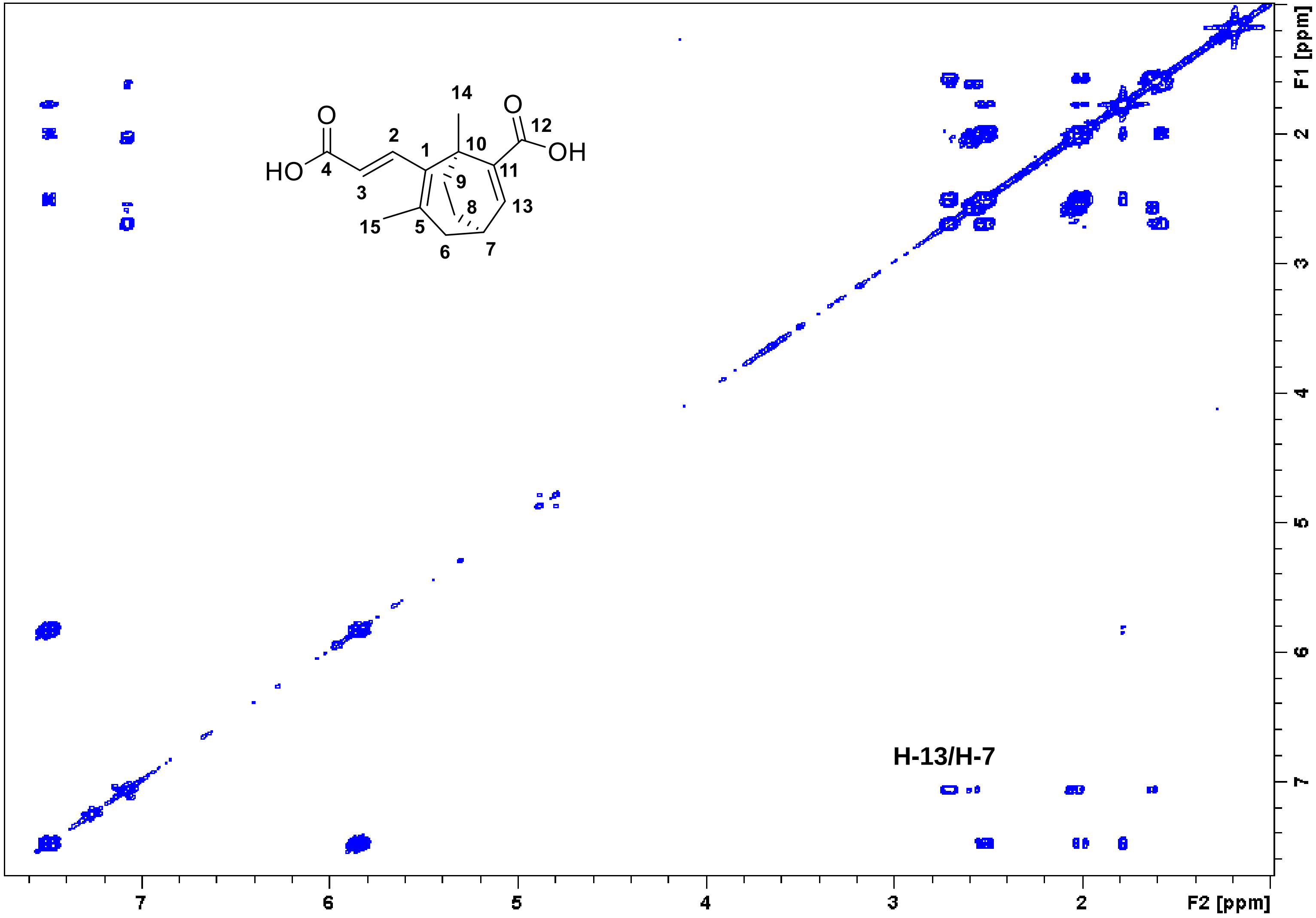

Figure S3-4. HSQC spectrum of compound **1** ($CDCl_3$, 400.13 MHz).

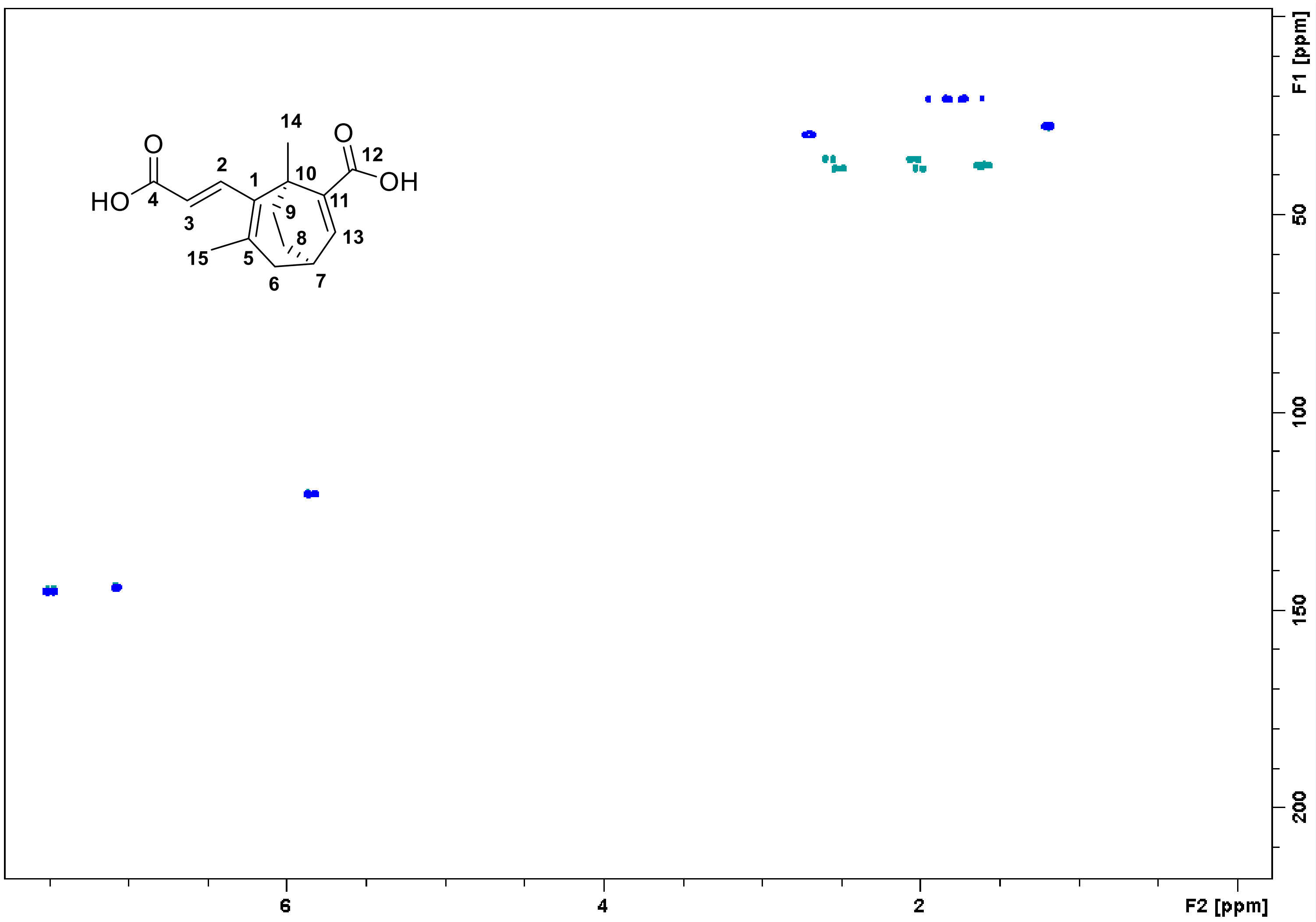

Figure S3-5. HMBC spectrum of compound **1** ($CDCl_3$, 400.13 MHz).

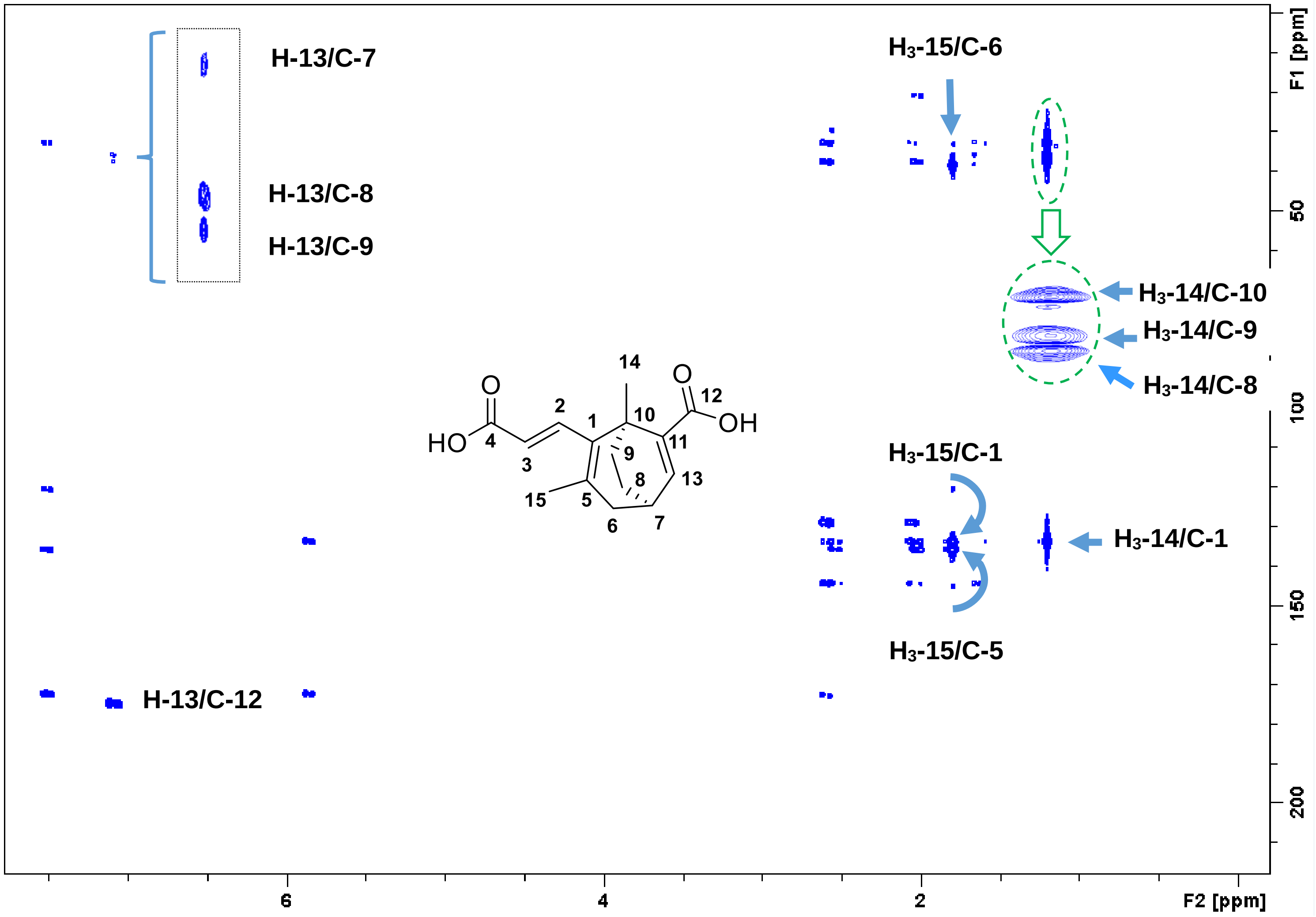

Figure S3-6. HRMS spectrum of compound **1.**

Meas. m/z	#	Ion Formula	Score	m/z	err [mDa]	err [ppm]	mSigma	rdb	e⁻ Conf	N-Rule	Adduct
285.109489	1	C15H18NaO4	100.00	285.109730	0.2	0.8	4.4	6.5	even	ok	M+Na

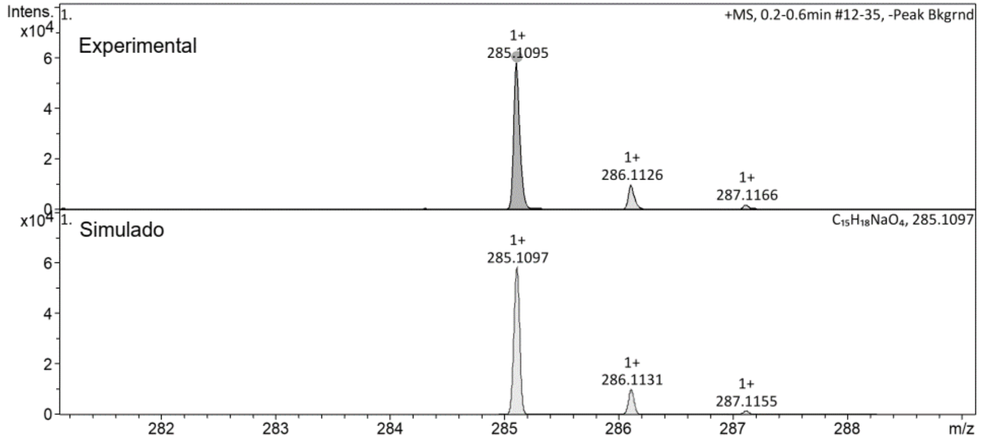

Figure S3-7. IR spectrum of compound **1.**

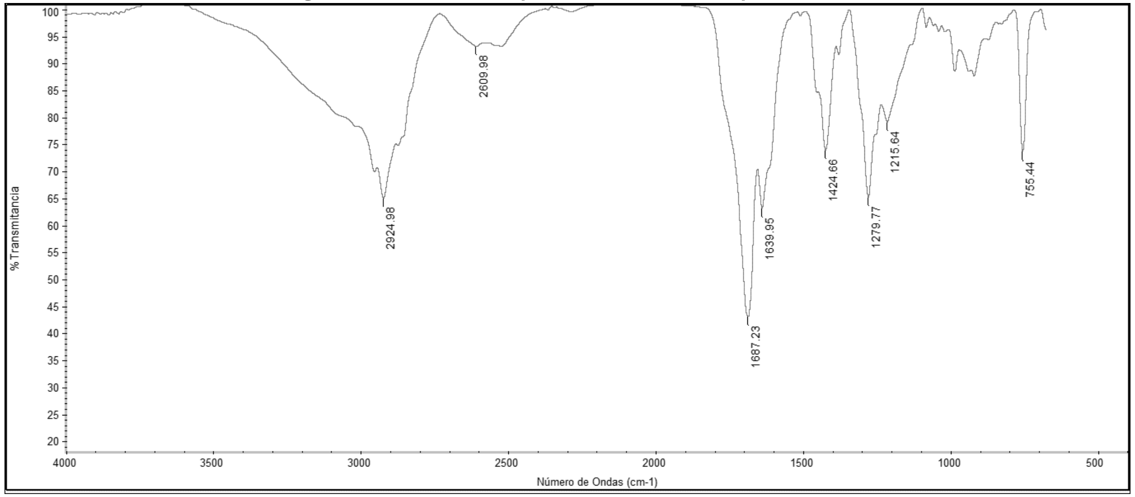

Figure S4-1. ^{1}H NMR spectrum of compound **2** ($CDCl_3$, 400.13 MHz).

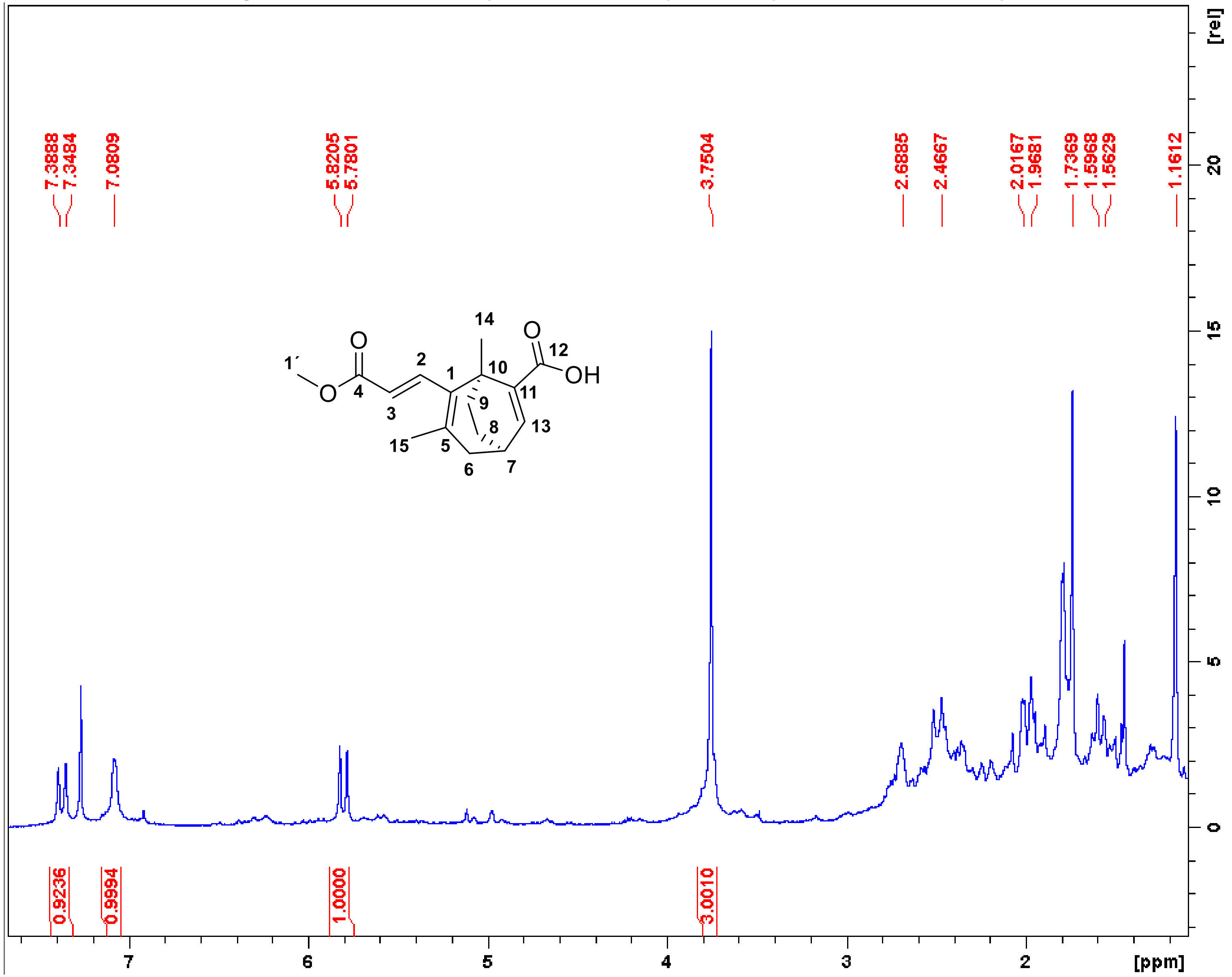

Figure S4-2. COSY spectrum of compound **2** ($CDCl_3$, 400.13 MHz).

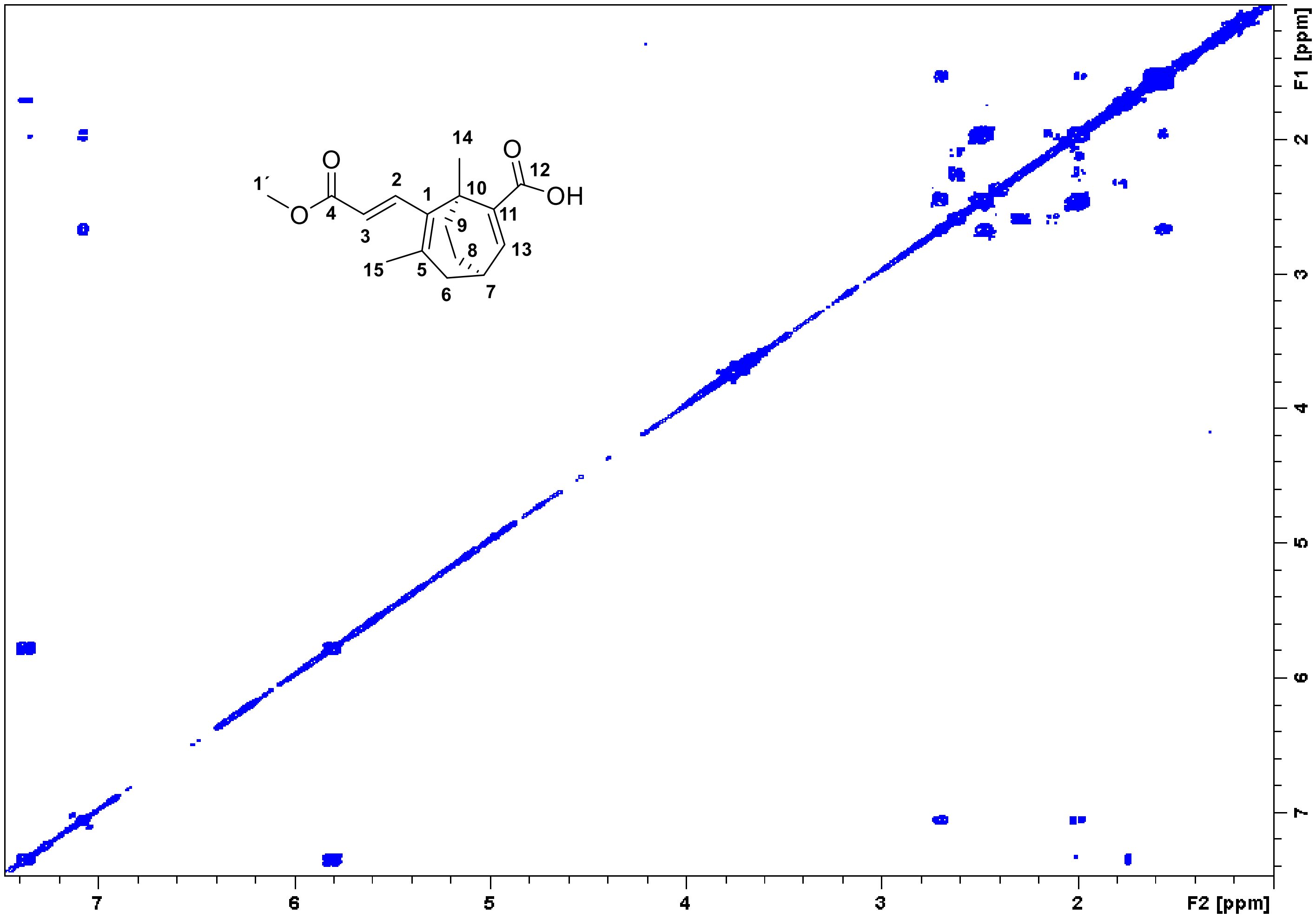

Figure S4-3. HSQC spectrum of compound **2** ($CDCl_3$, 400.13 MHz).

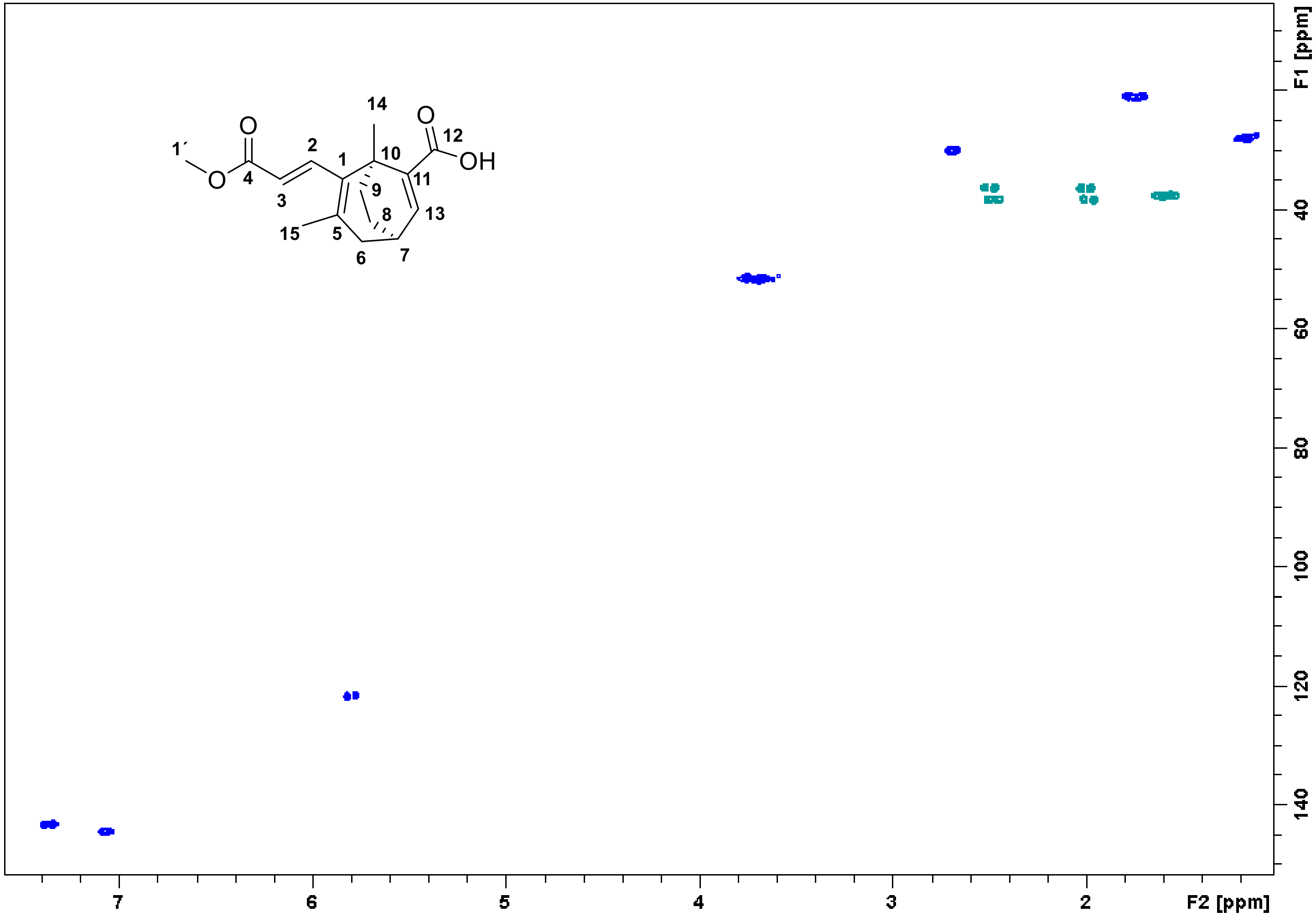

Figure S4-4. HMBC spectrum of compound **2** ($CDCl_3$, 400.13 MHz).

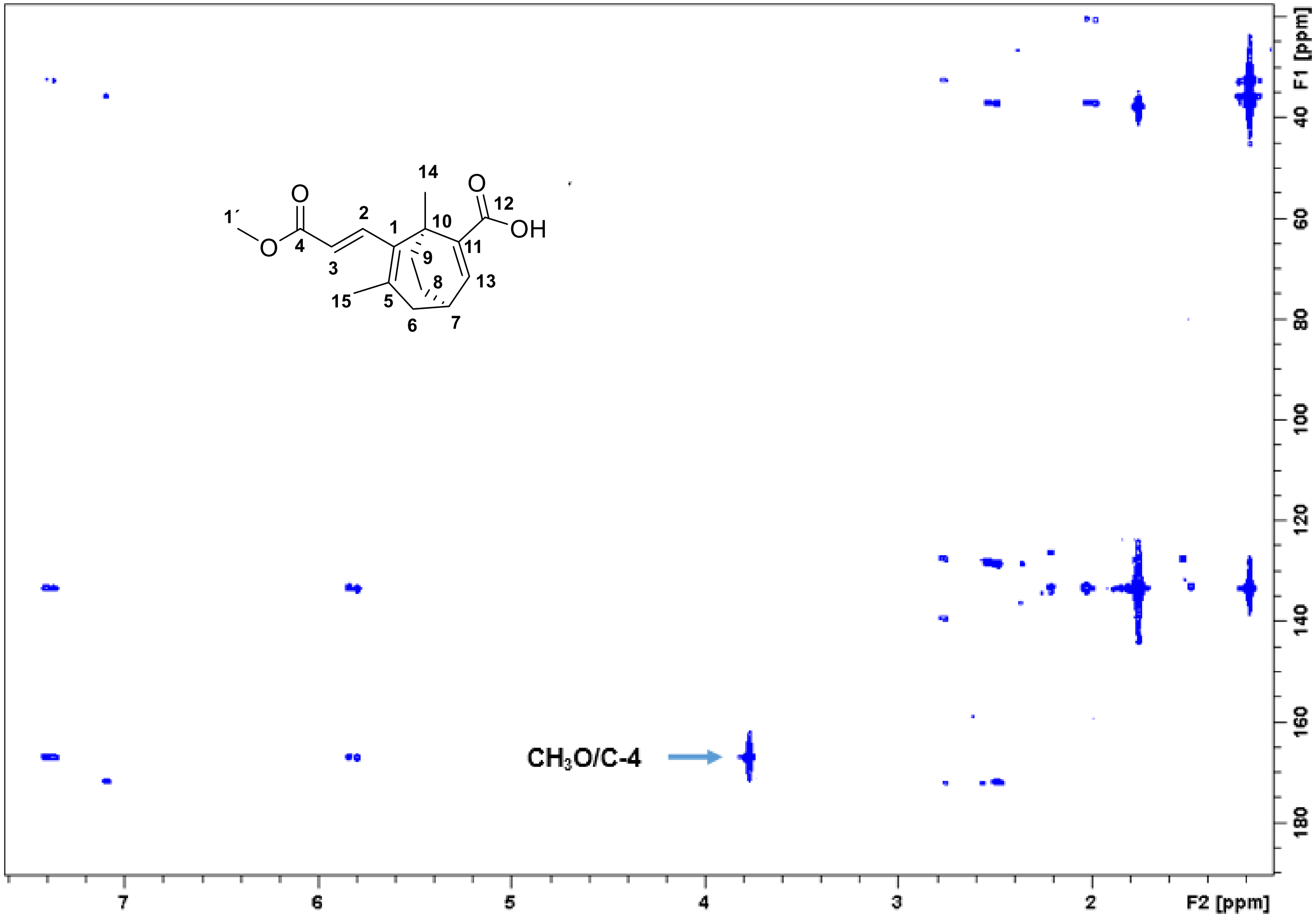

Figure S4-5. HRMS spectrum of compound **2.**

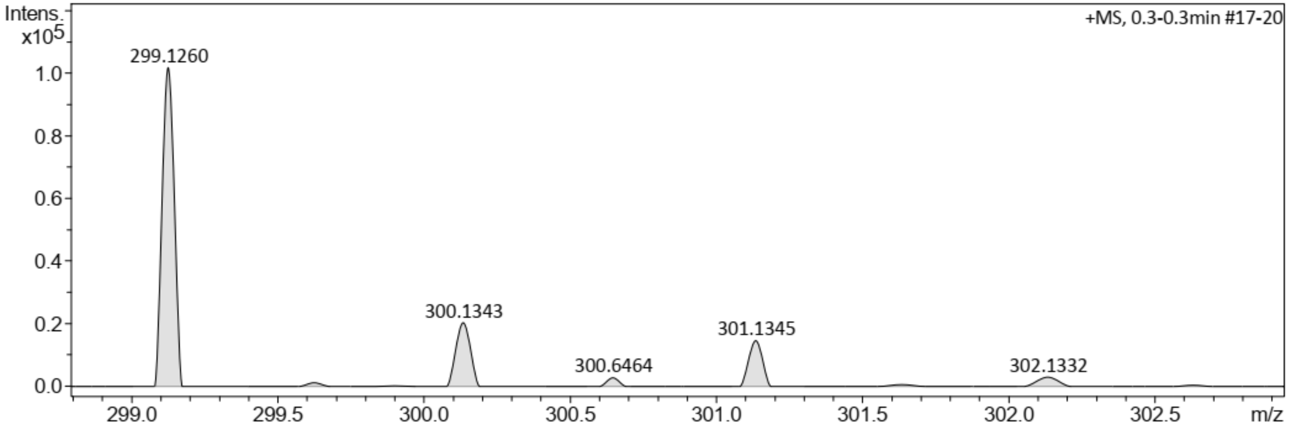

Meas. m/z	#	Ion Formula	m/z	err [ppm]	mSigma	# mSigma	Score	rdb	e⁻ Conf	N-Rule
299.1260	1	C16H20NaO4	299.1254	-2.2	70.2	2	86.58	6.5	even	ok

Figure S4-6. IR spectrum of compound **2.**

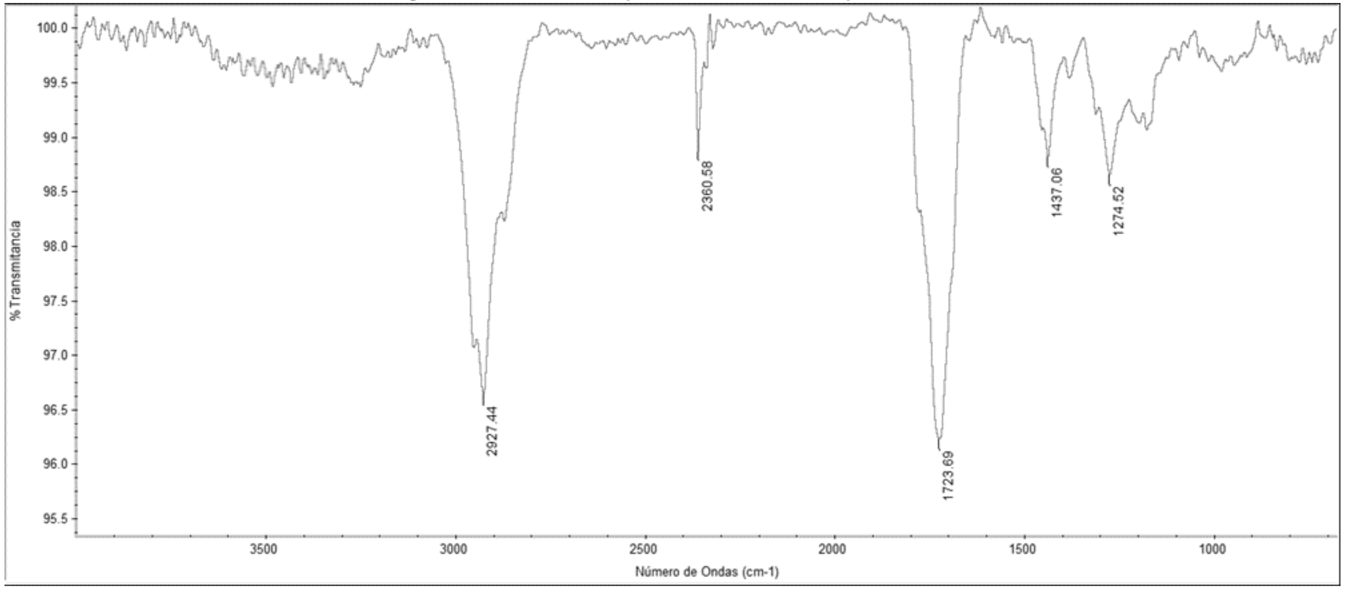

Figure S5-1. ^1H NMR spectrum of compound **3** ($CDCl_3$, 400.13 MHz).

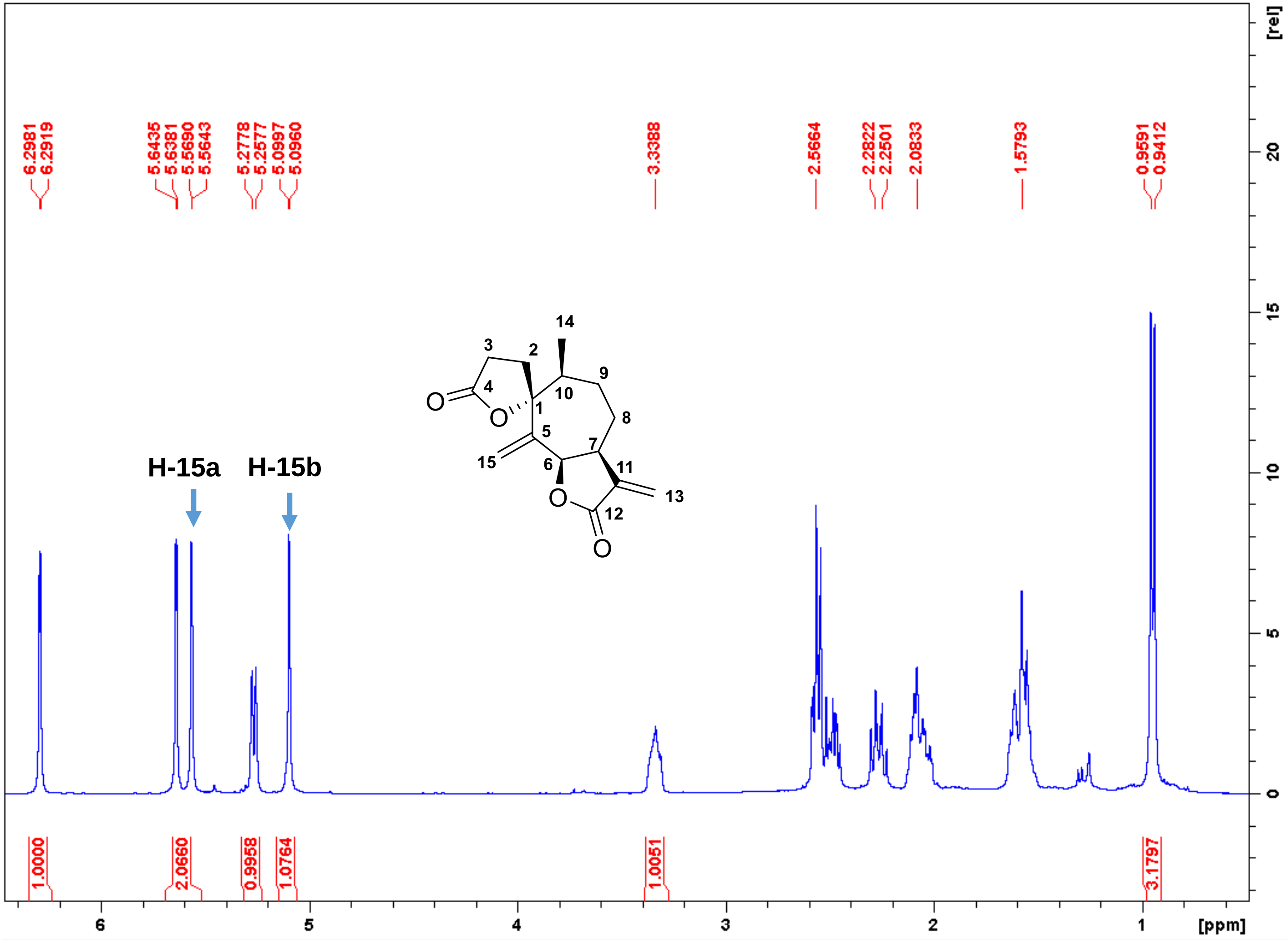

Figure S5-2. ^{13}C NMR spectrum of compound **3** ($CDCl_3$, 100.03 MHz).

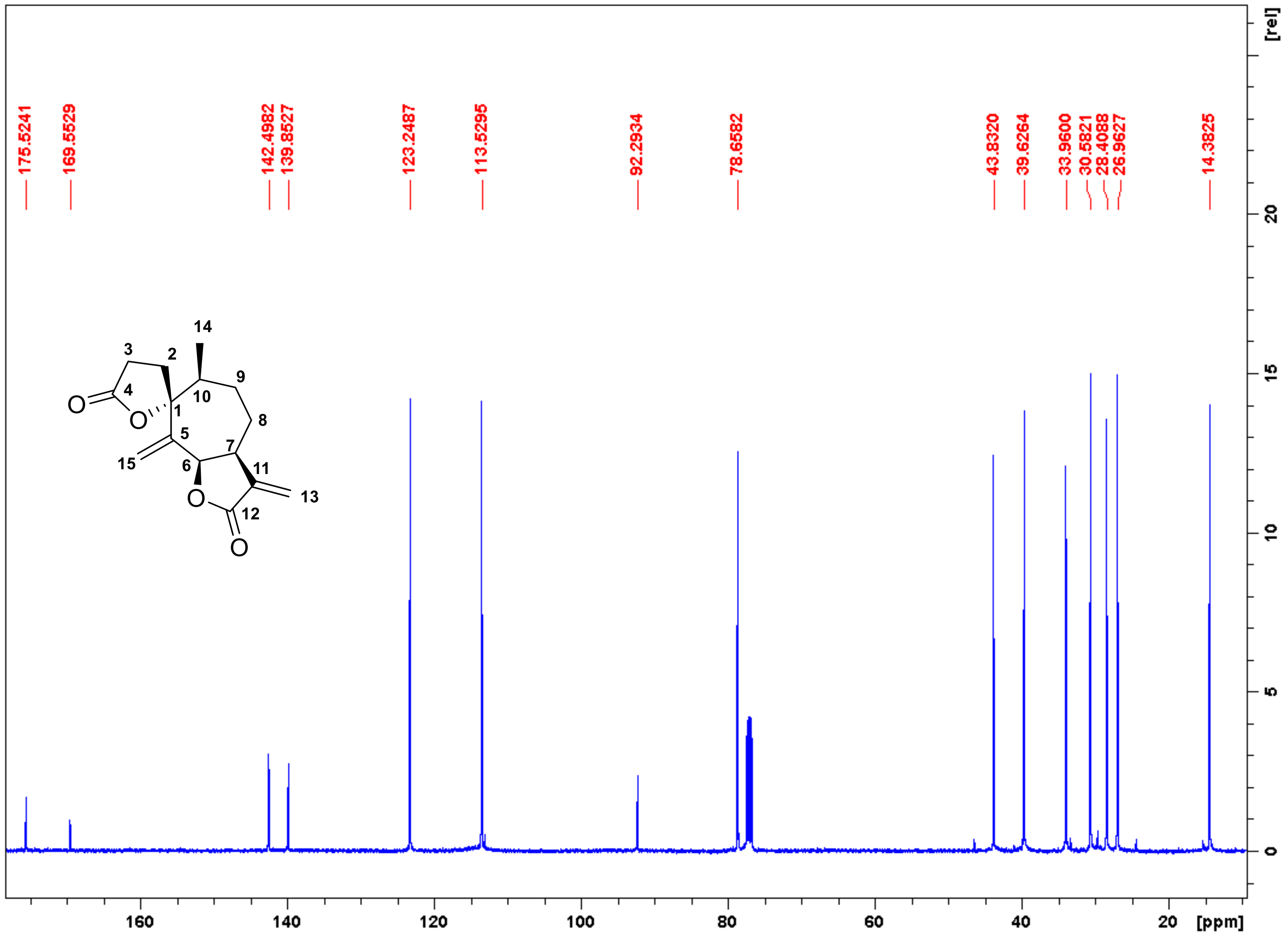

Figure S5-3. COSY spectrum of compound **3** ($CDCl_3$, 400.13 MHz).

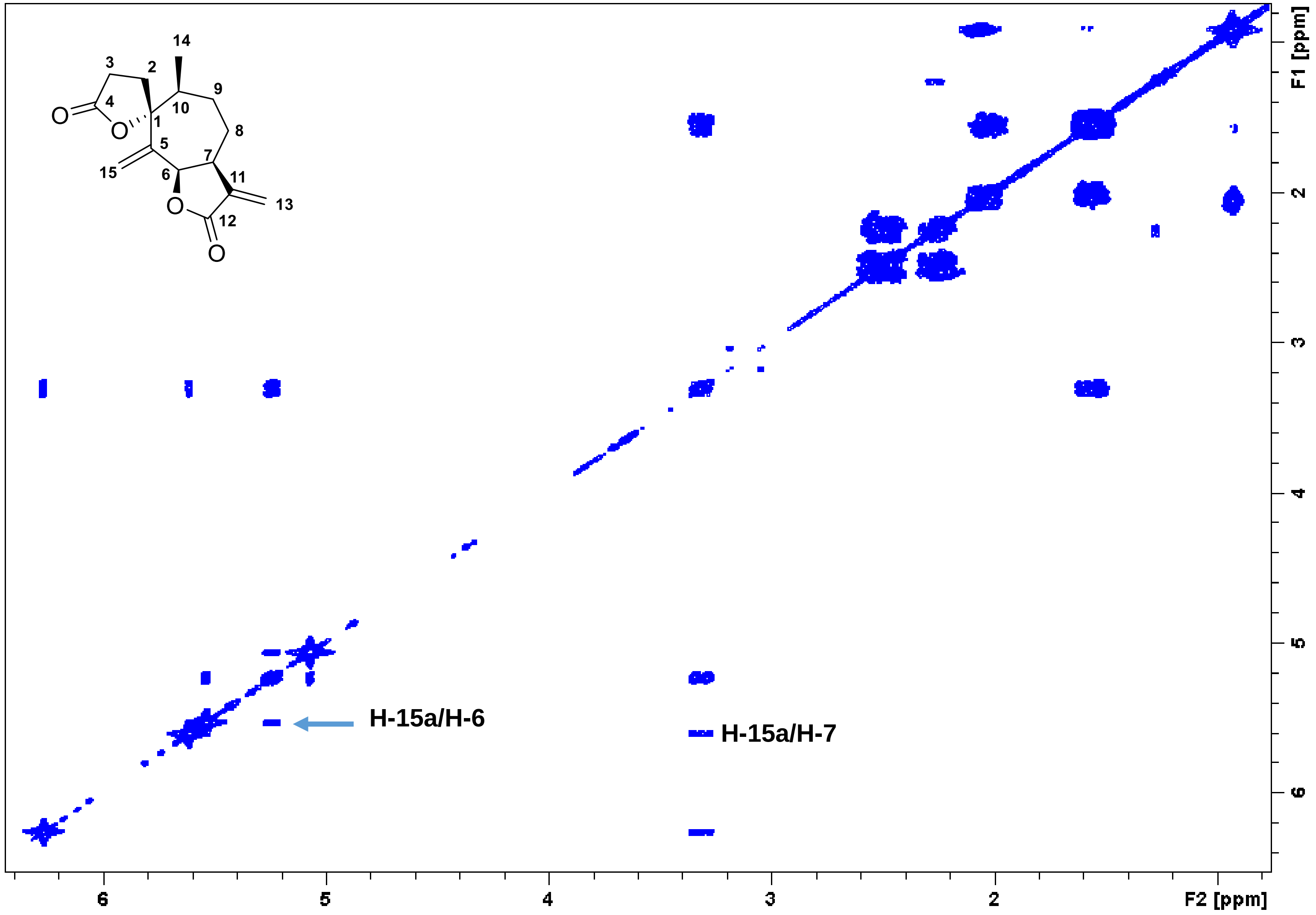

Figure S5-4. HSQC spectrum of compound **3** ($CDCl_3$, 400.13 MHz).

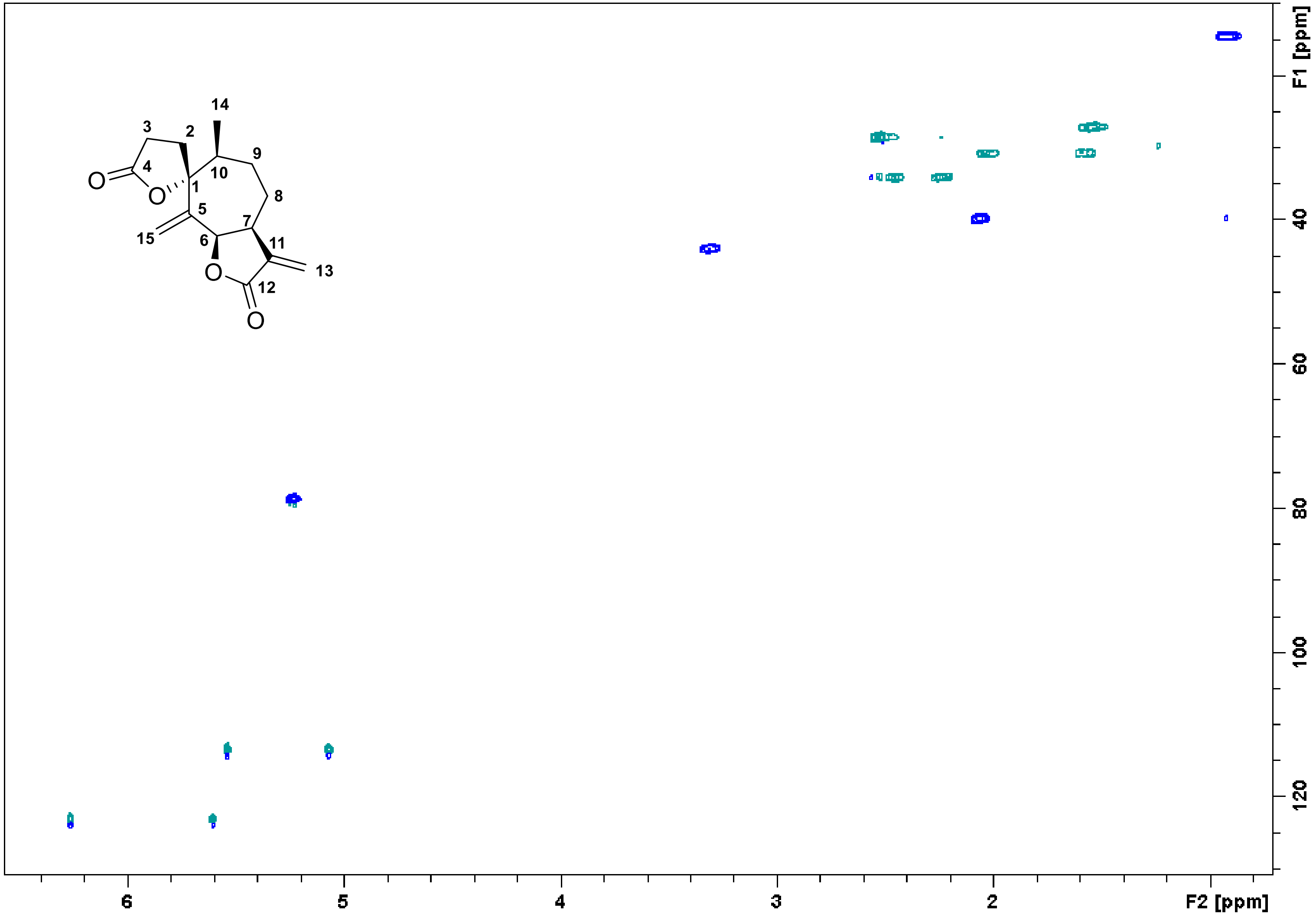

Figure S5-5. HMBC spectrum of compound **3** ($CDCl_3$, 400.13 MHz).

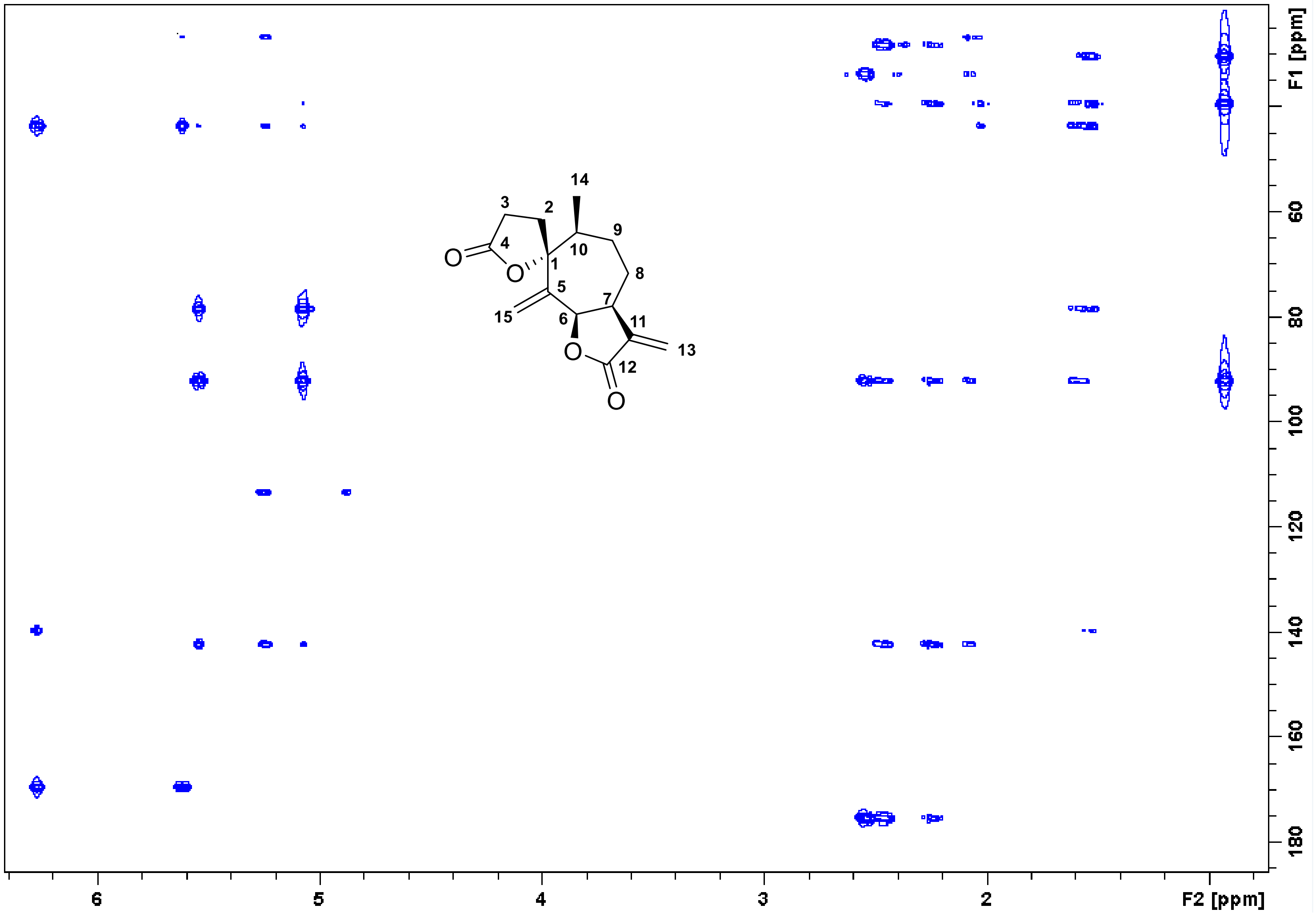

Figure S5-6. NOESY spectrum of compound **3** ($CDCl_3$, 400.13 MHz).

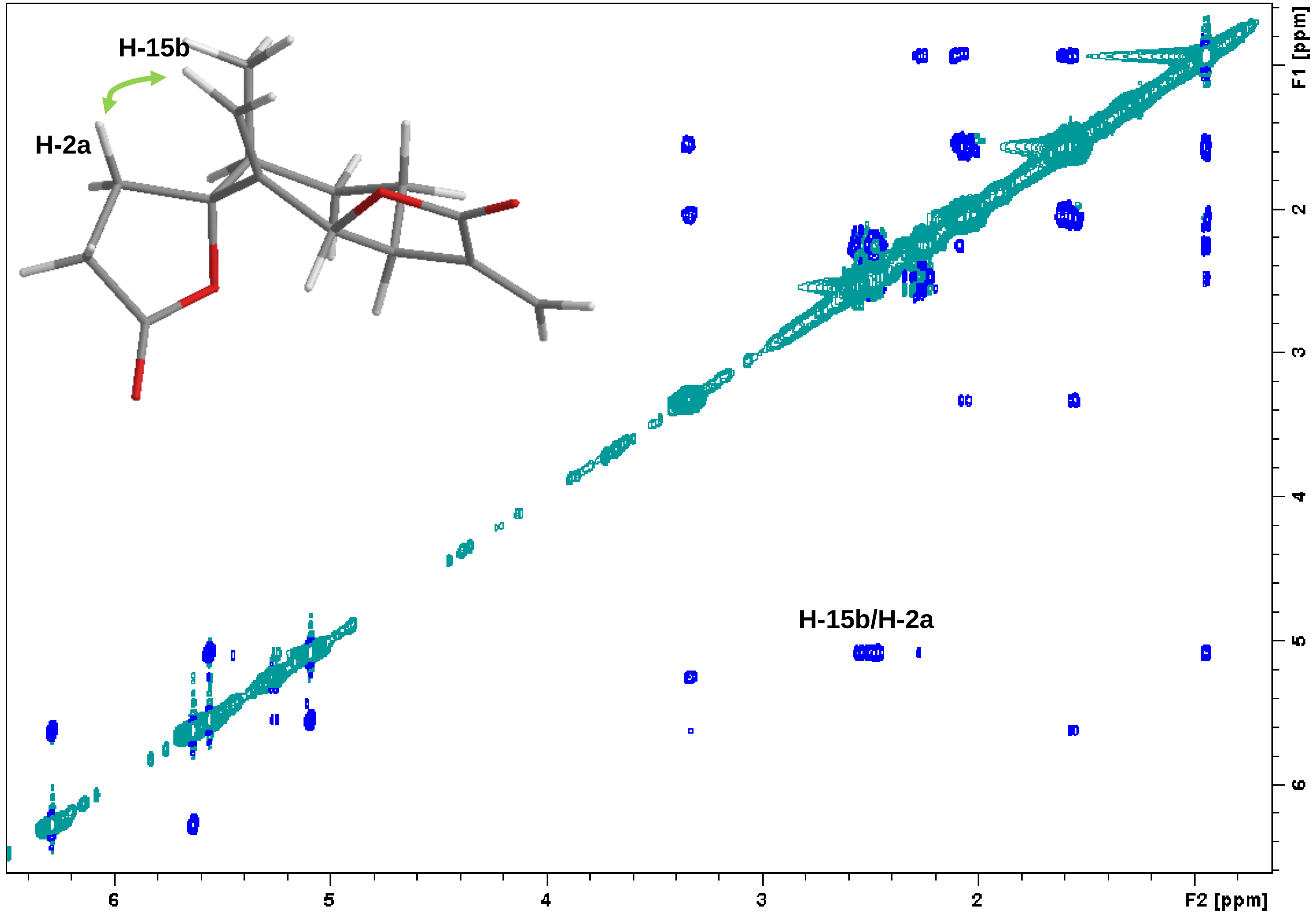

Figure S5-7. HRMS spectrum of compound **3.**

Meas. m/z	#	Ion Formula	Score	m/z	err [mDa]	err [ppm]	mSigma	rdb	e¯ Conf	N-Rule	Adduct
285.109949	1	C15H18NaO4	100.00	285.109730	-0.2	-0.8	29.8	6.5	even	ok	M+Na
	2	C14H16N2NaO3	0.00	283.105313	-0.9	-3.2	813.5	7.5	even	ok	M+Na

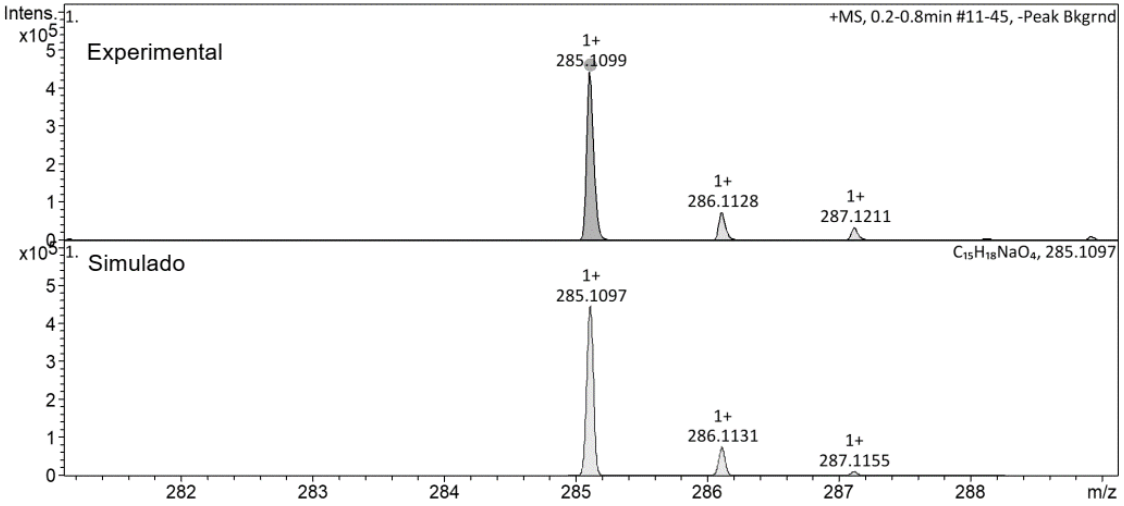

Figure S5-8. IR spectrum of compound **3.**

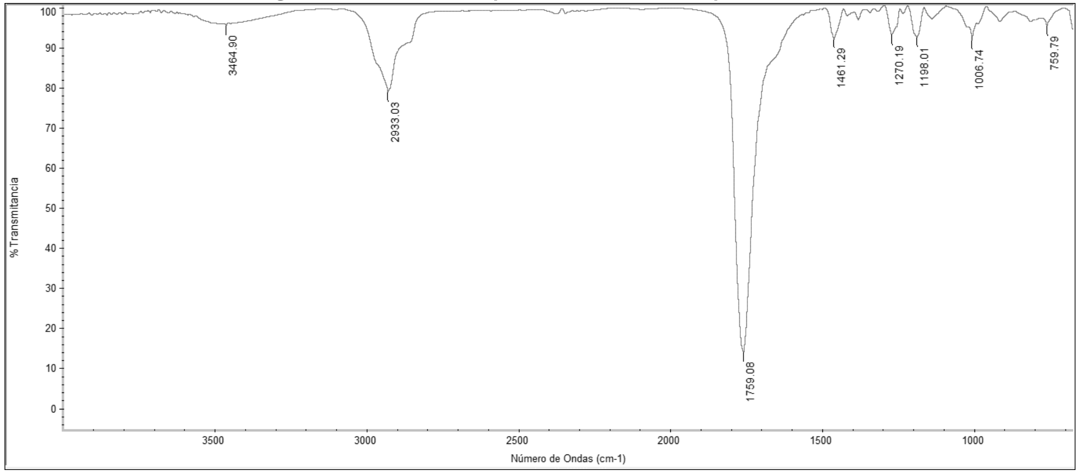

Figure S6-1. ^{1}H NMR spectrum of compound **4** ($CDCl_3$, 400.13 MHz).

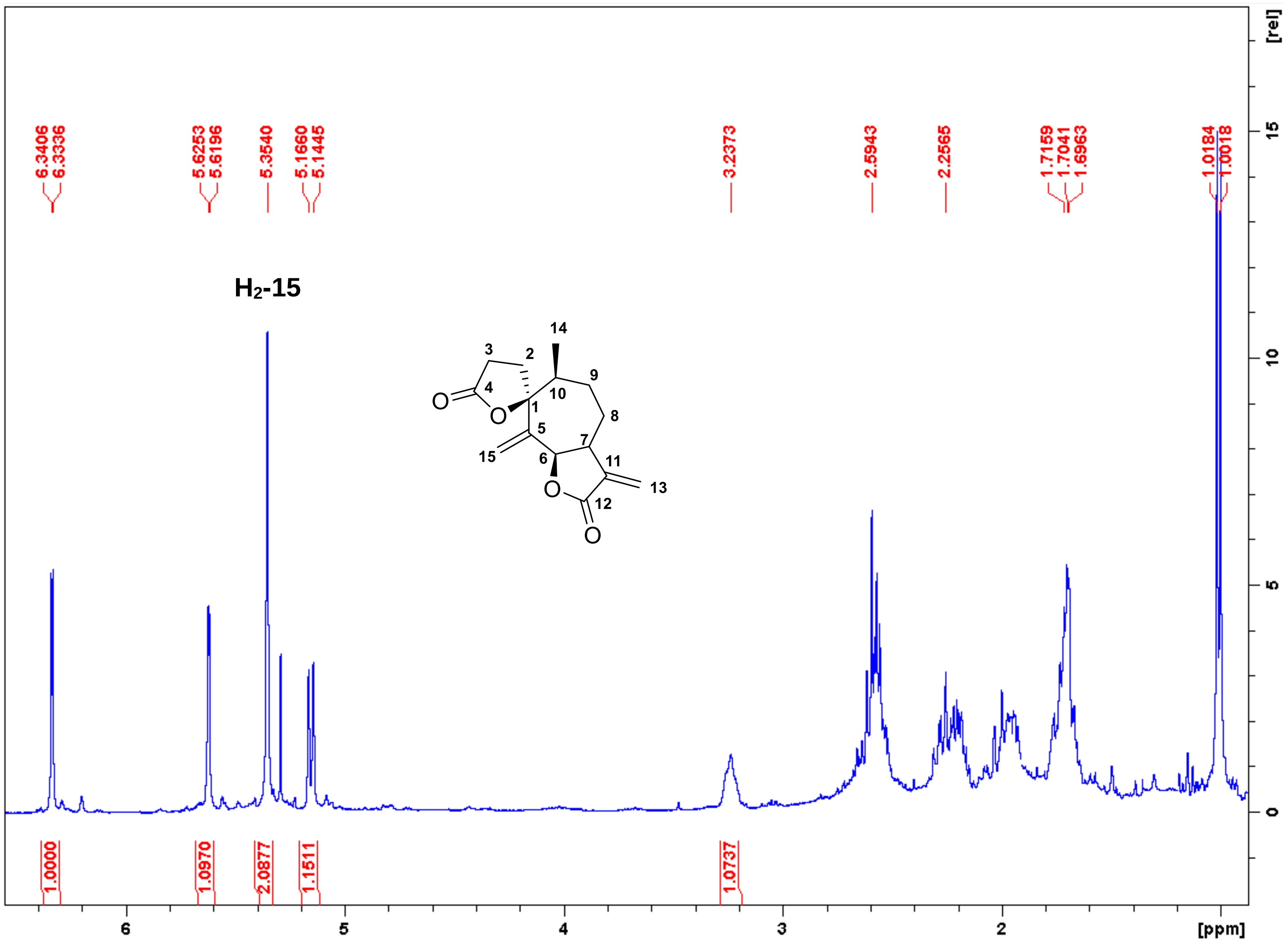

Figure S6-2. COSY spectrum of compound **4** ($CDCl_3$, 400.13 MHz).

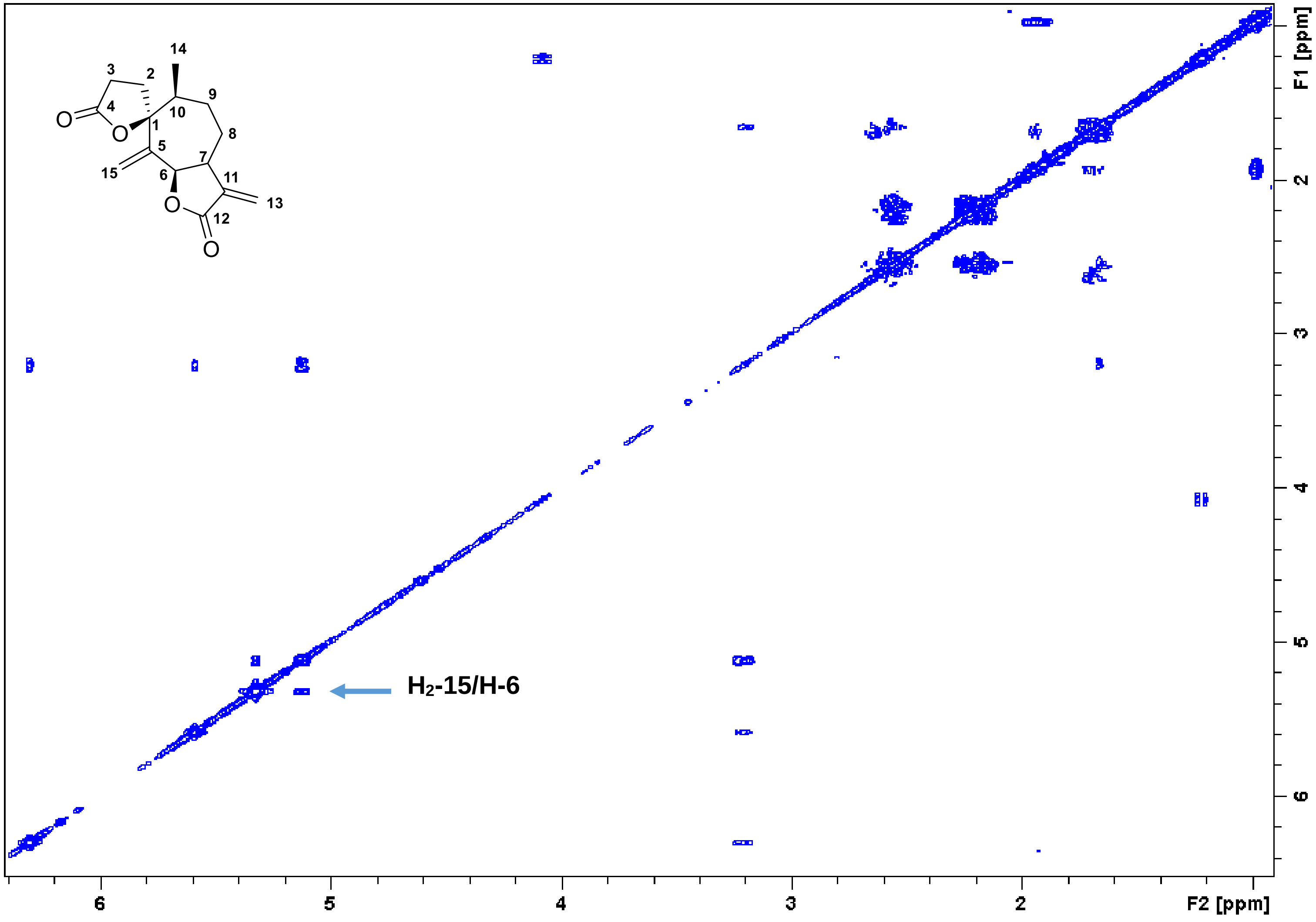

Figure S6-3. HSQC spectrum of compound **4** ($CDCl_3$, 400.13 MHz).

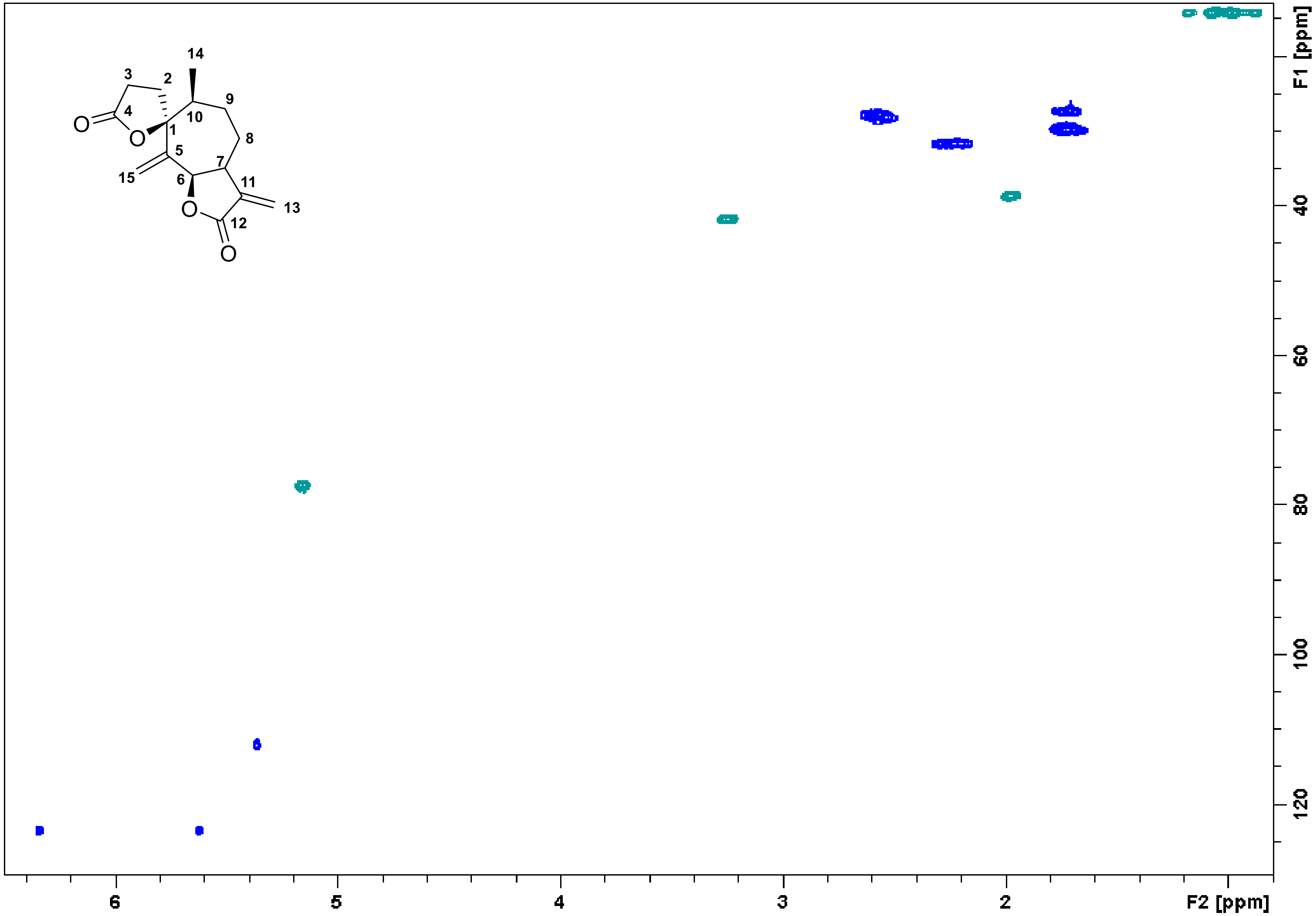

Figure S6-4. HMBC spectrum of compound **4** ($CDCl_3$, 400.13 MHz).

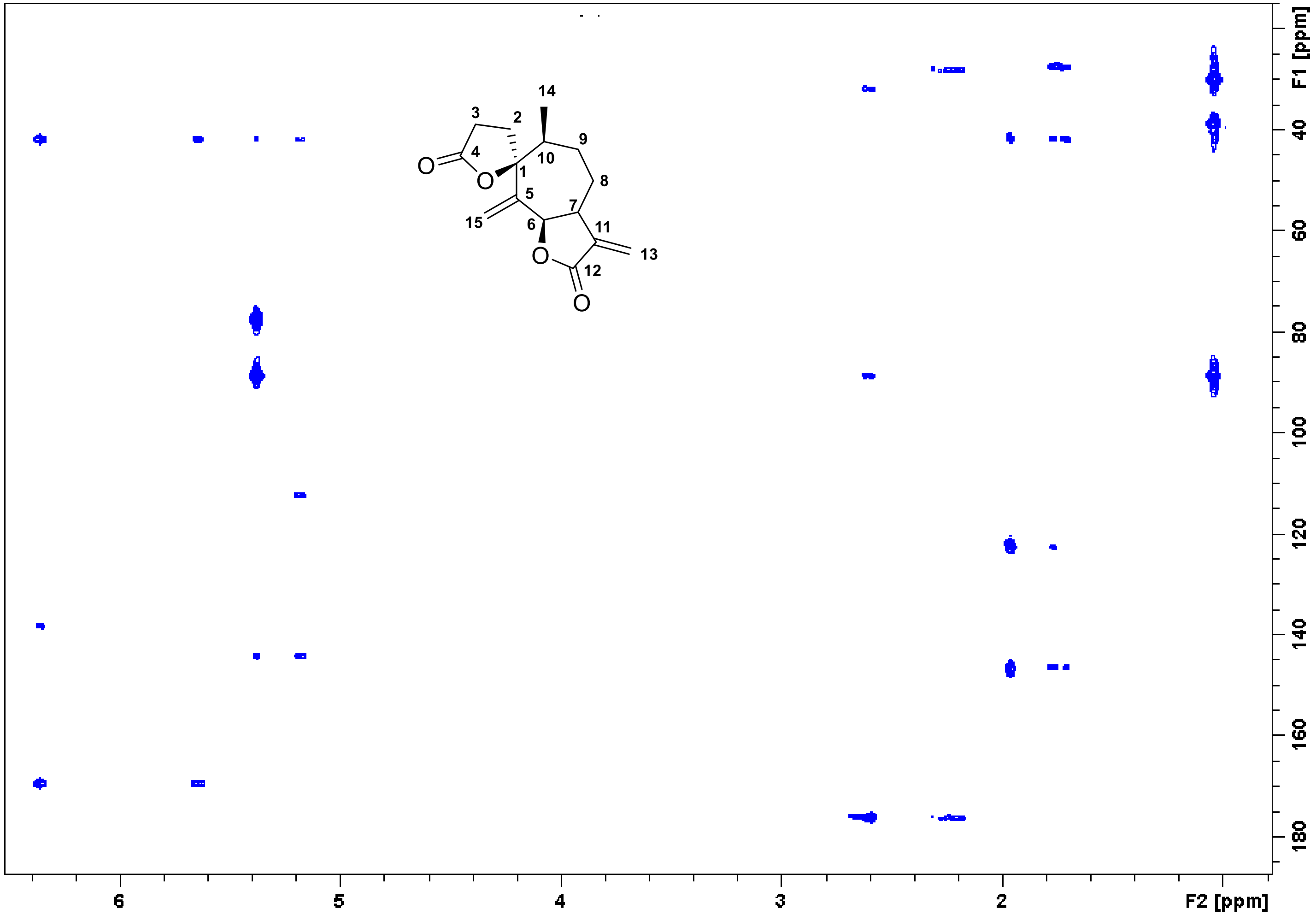

Figure S6-5. NOESY spectrum of compound **4** ($CDCl_3$, 400.13 MHz). AH 2 29

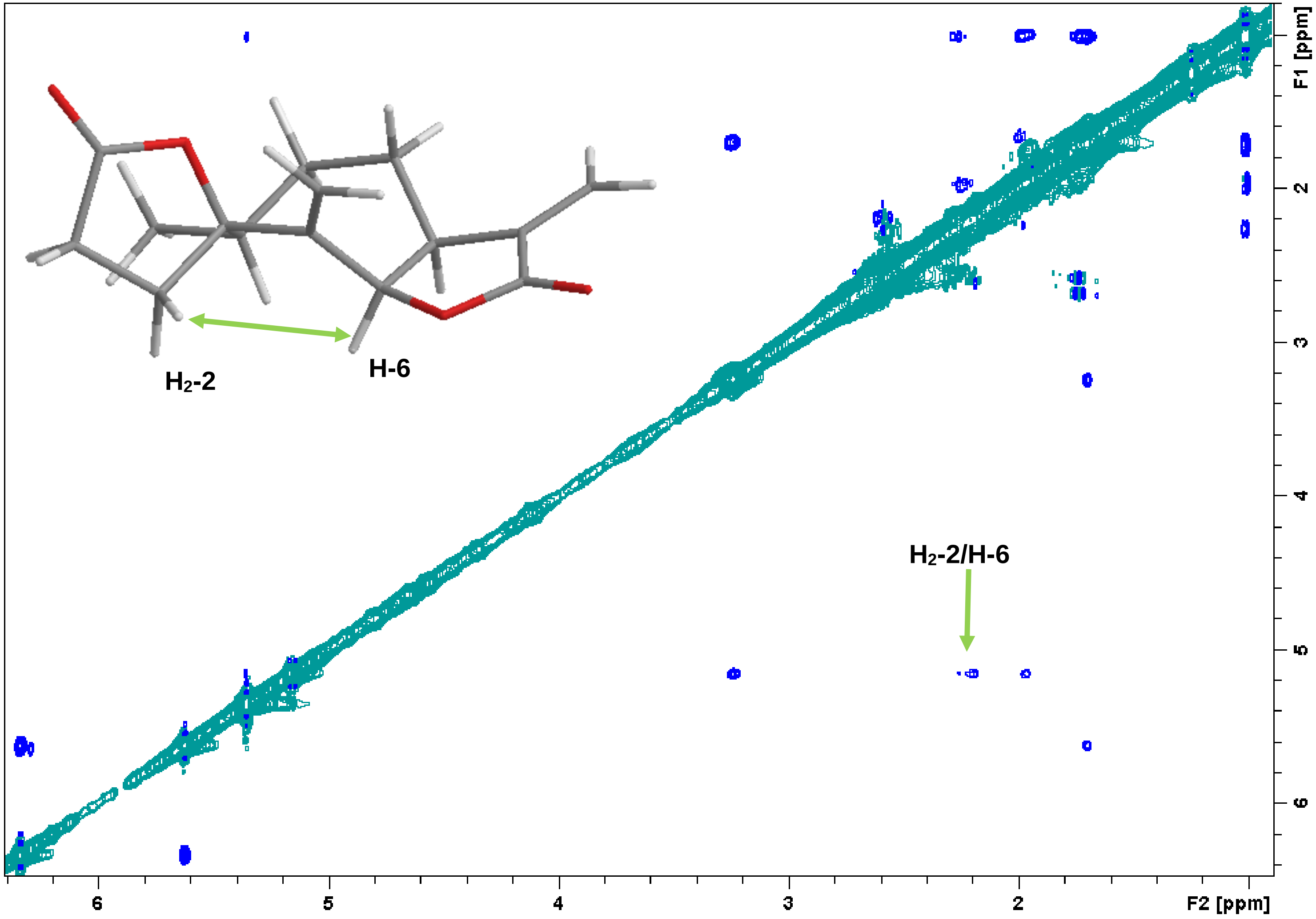

Figure S6-6. HRMS spectrum of compound **4.**

Meas. m/z	#	Ion Formula	Score	m/z	err [mDa]	err [ppm]	mSigma	rdb	e⁻ Conf	N-Rule	Adduct
285.108504	1	C15H18NaO4	100.00	285.109730	-1.2	-4.3	222.6	6.5	even	ok	M+Na
	2	C14H16N2NaO3	0.00	283.105313	-2.4	-8.3	811.8	7.5	even	ok	M+Na

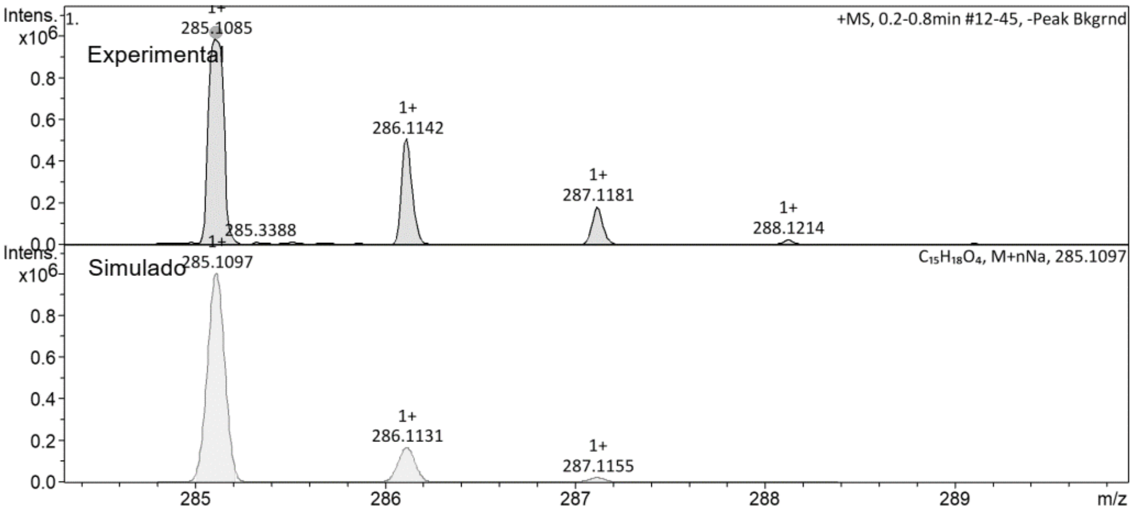

Figure S6-7. IR spectrum of compound **4.**

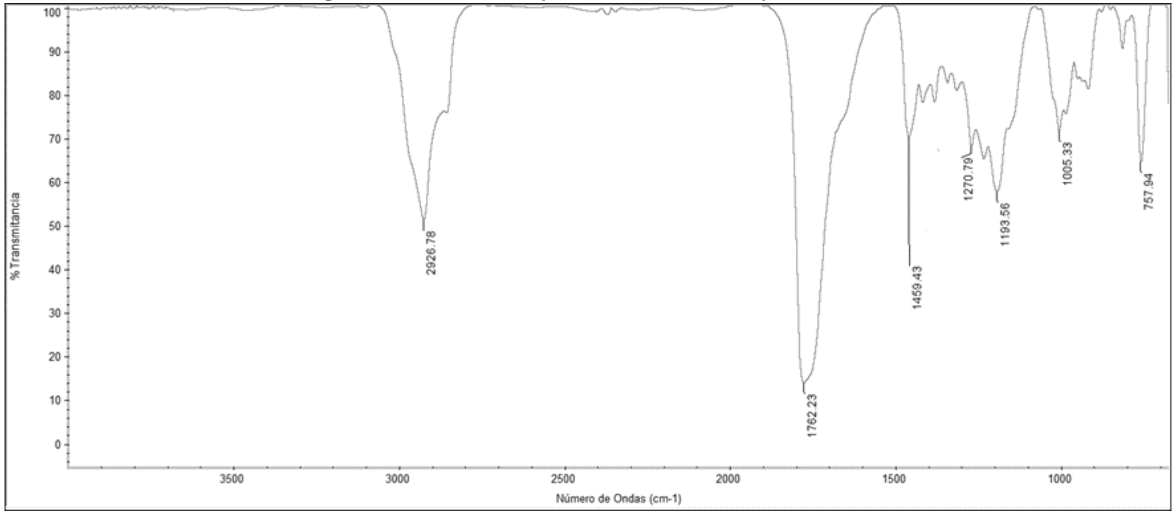

Figure S7-1. ^{1}H NMR spectrum of compound **5** ($CDCl_3$, 400.13 MHz).

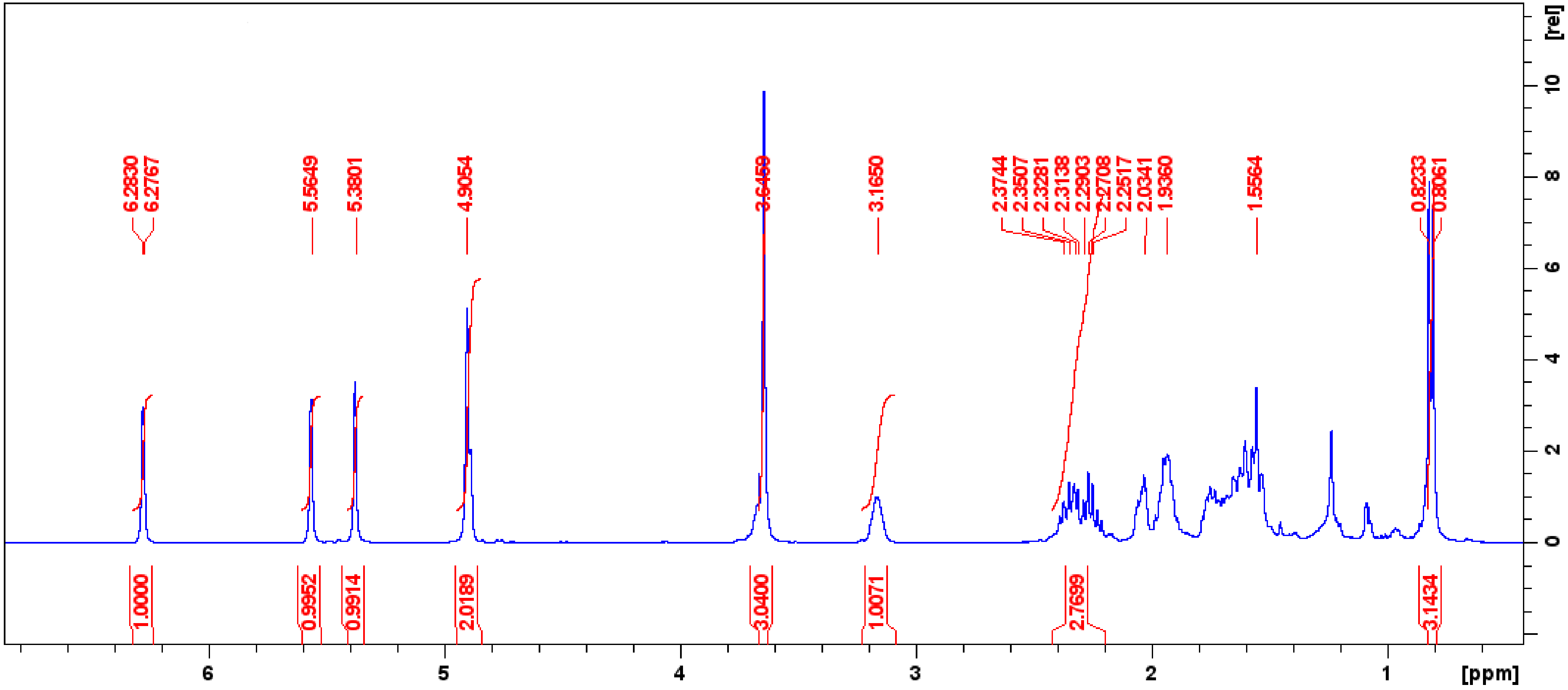

Figure S8-1. ^{1}H NMR spectrum of compound **6** ($CDCl_3$, 400.13 MHz).

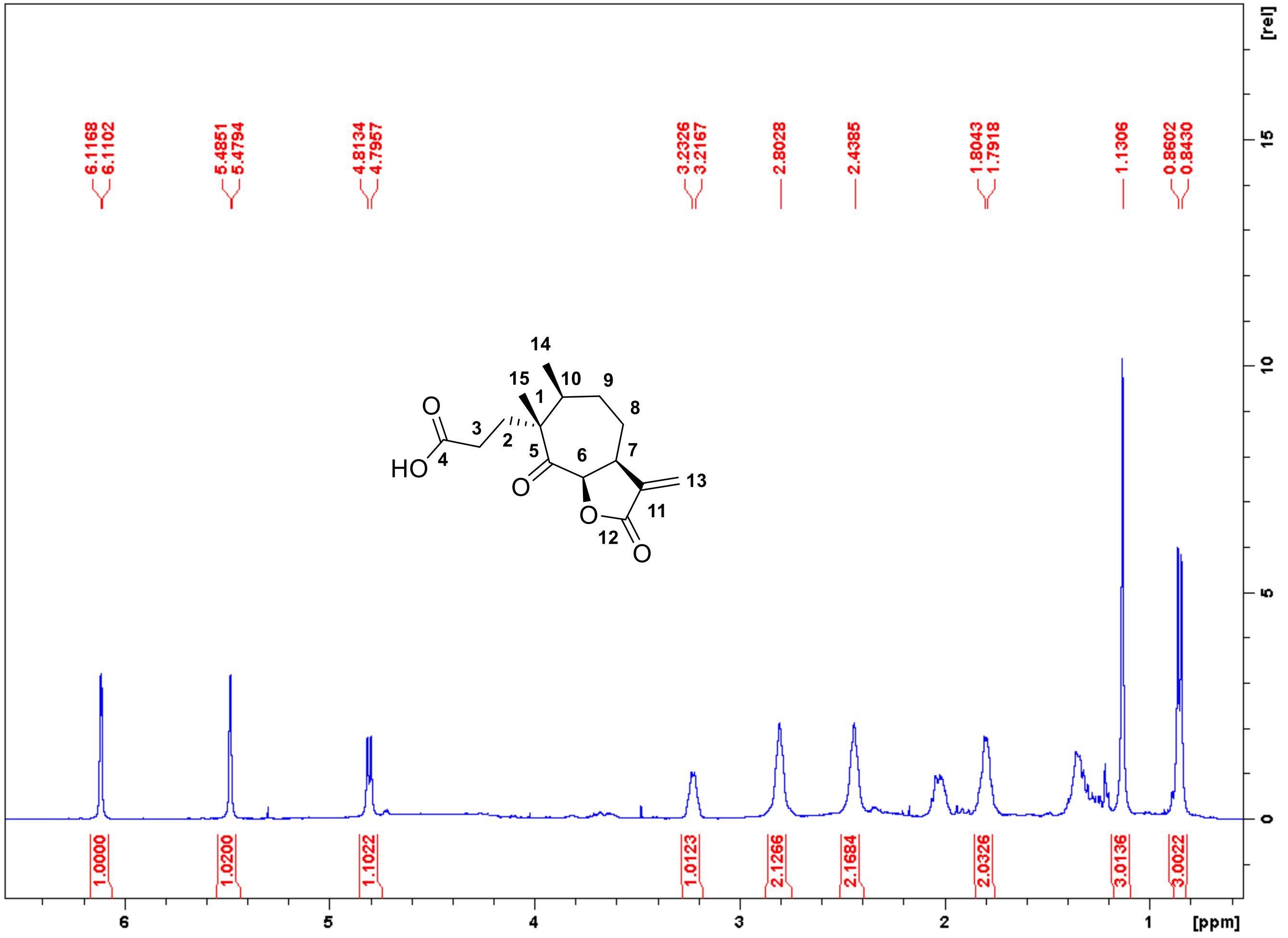

Figure S8-2. ^{13}C NMR spectrum of compound **6** ($CDCl_3$, 100.03 MHz).

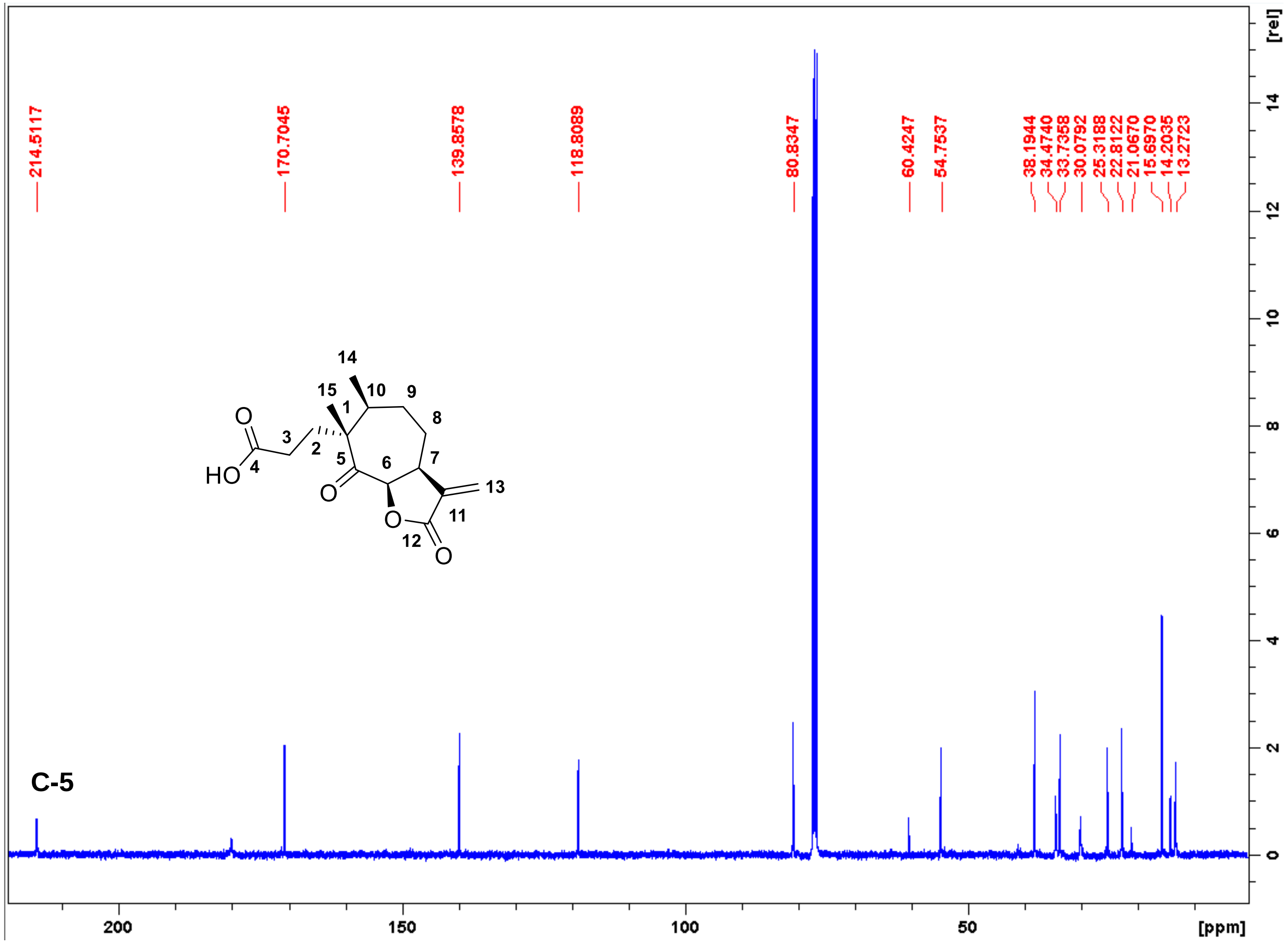

Figure S8-3. COSY spectrum of compound **6** ($CDCl_3$, 400.13 MHz).

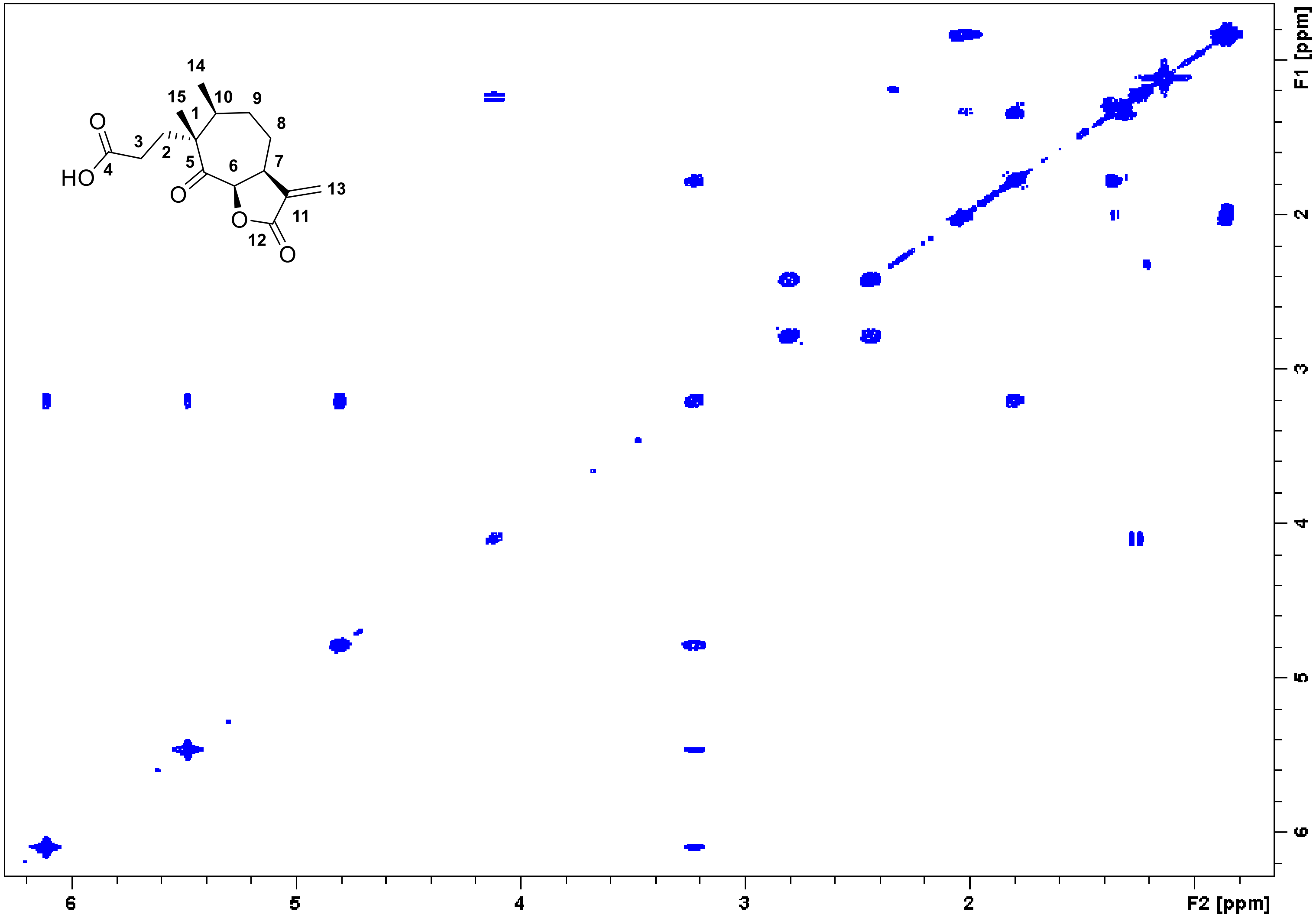

Figure S8-4. HSQC spectrum of compound **6** ($CDCl_3$, 400.13 MHz).

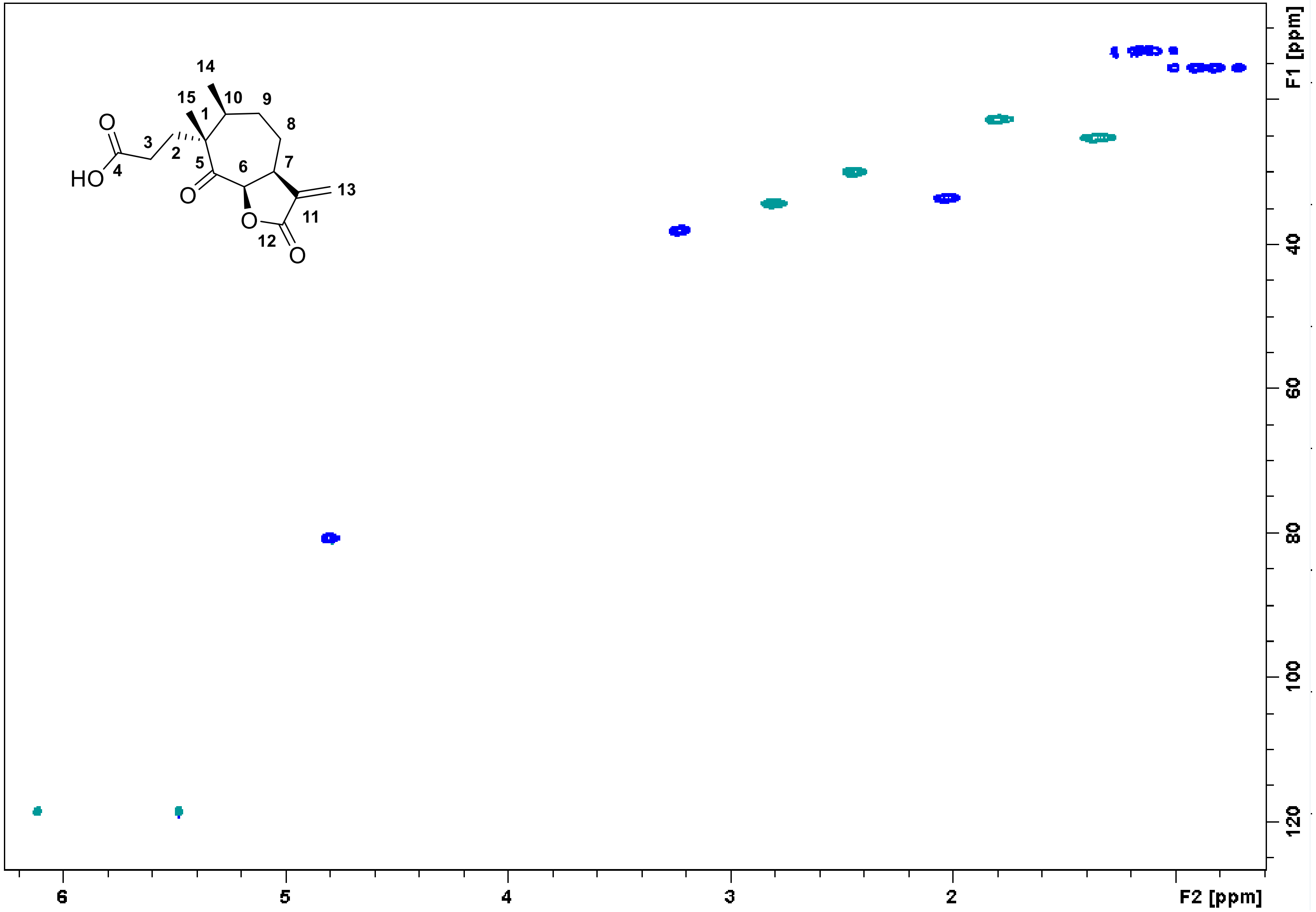

Figure S8-5. HMBC spectrum of compound **6** ($CDCl_3$, 400.13 MHz).

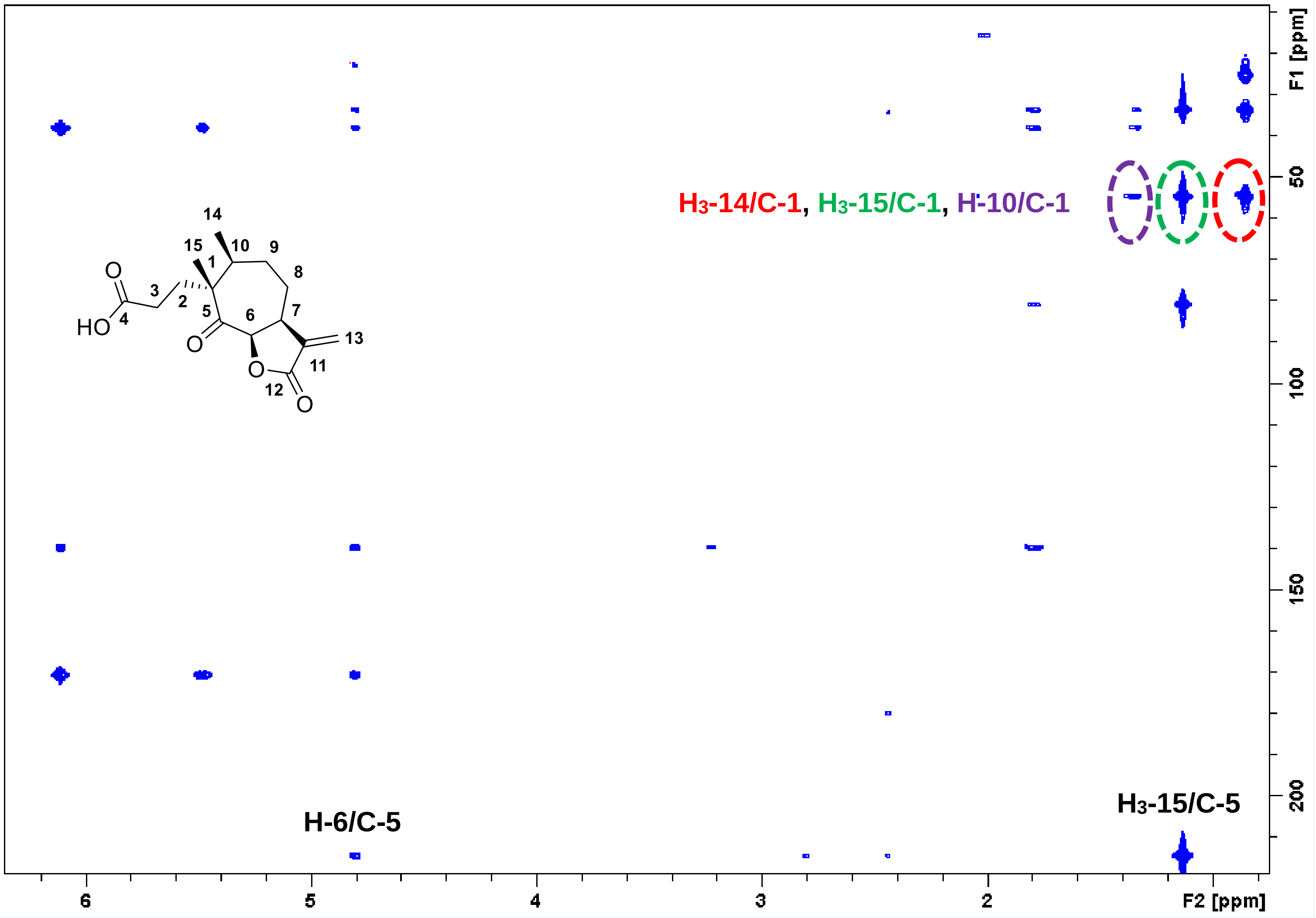

Figure S8-6. NOESY spectrum of compound **6** ($CDCl_3$, 400.13 MHz).

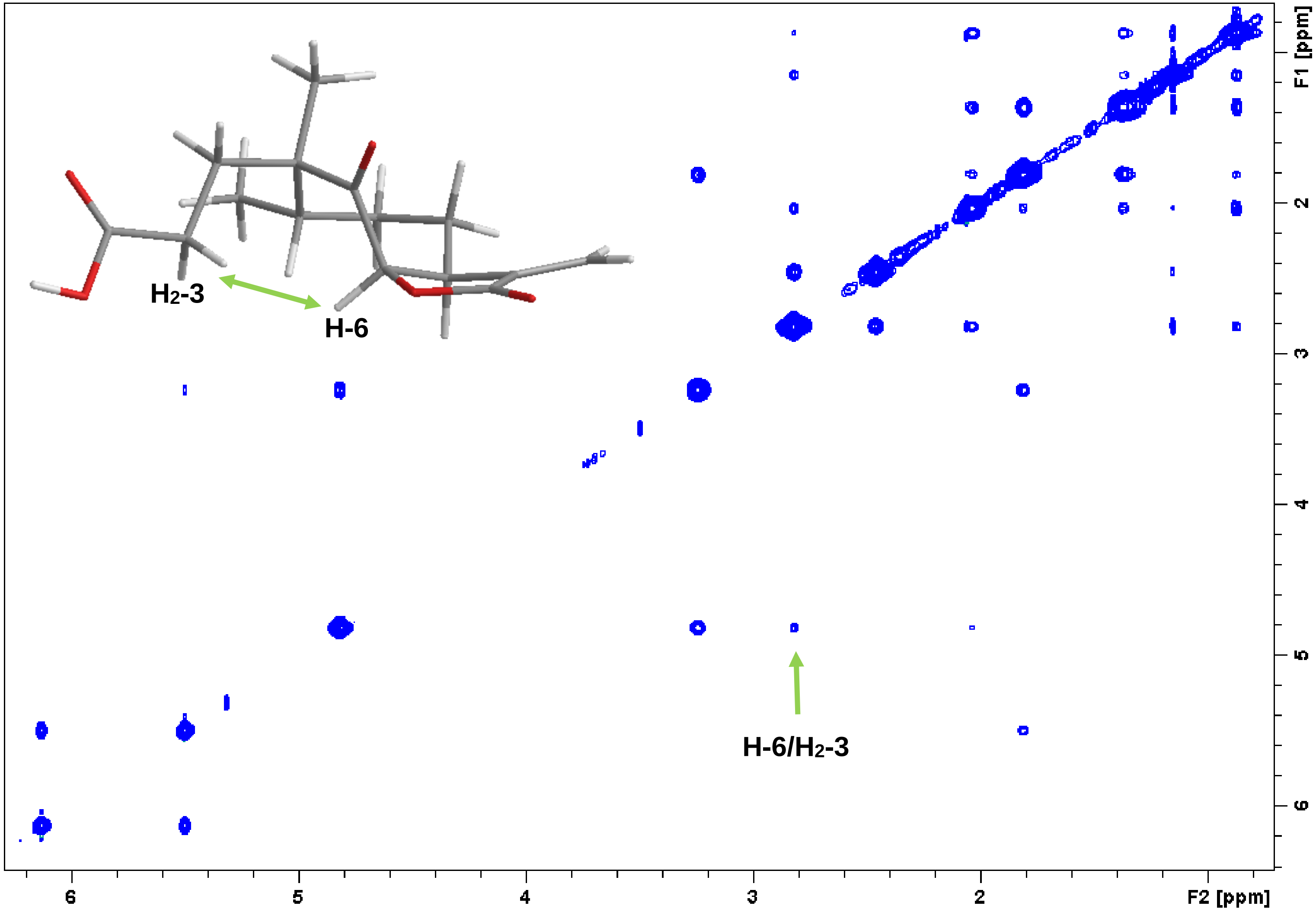

Figure S8-7. HRMS spectrum of compound **6.**

Meas. m/z	#	Ion Formula	Score	m/z	err [mDa]	err [ppm]	mSigma	rdb	e⁻ Conf	N-Rule	Adduct
303.120800	1	C15H20NaO5	100.00	303.120294	-0.5	-1.7	144.5	5.5	even	ok	M+Na
	2	C14H18N2NaO4	0.00	301.115878	-0.5	-1.7	774.9	6.5	even	ok	M+Na

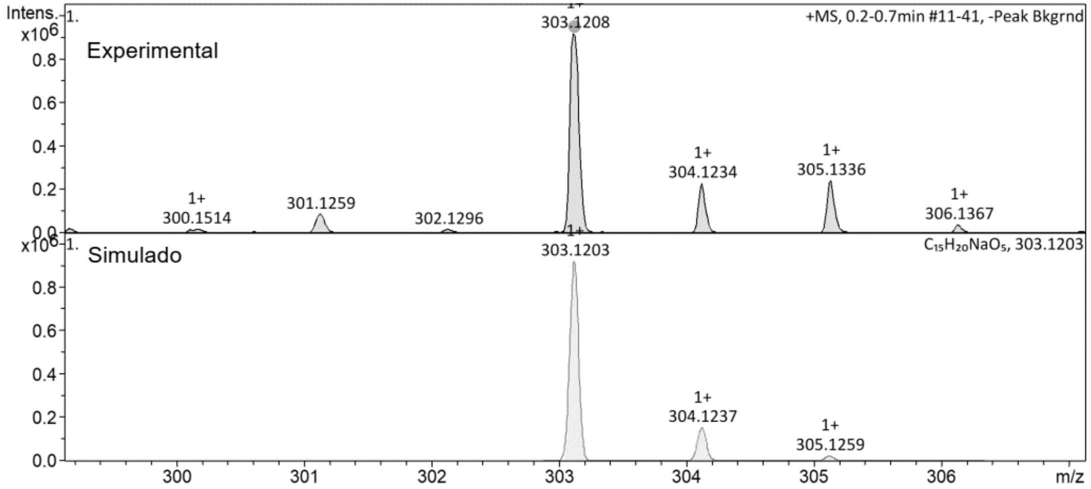

Figure S8-8. IR spectrum of compound **6.**

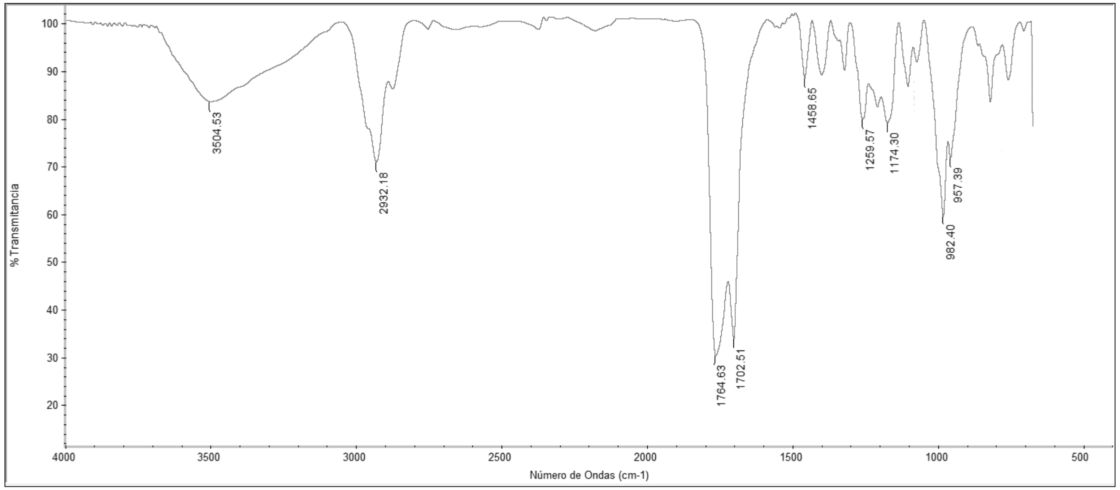

Figure S9-1. ^{1}H NMR spectrum of compound **7** ($CDCl_3$, 400.13 MHz).

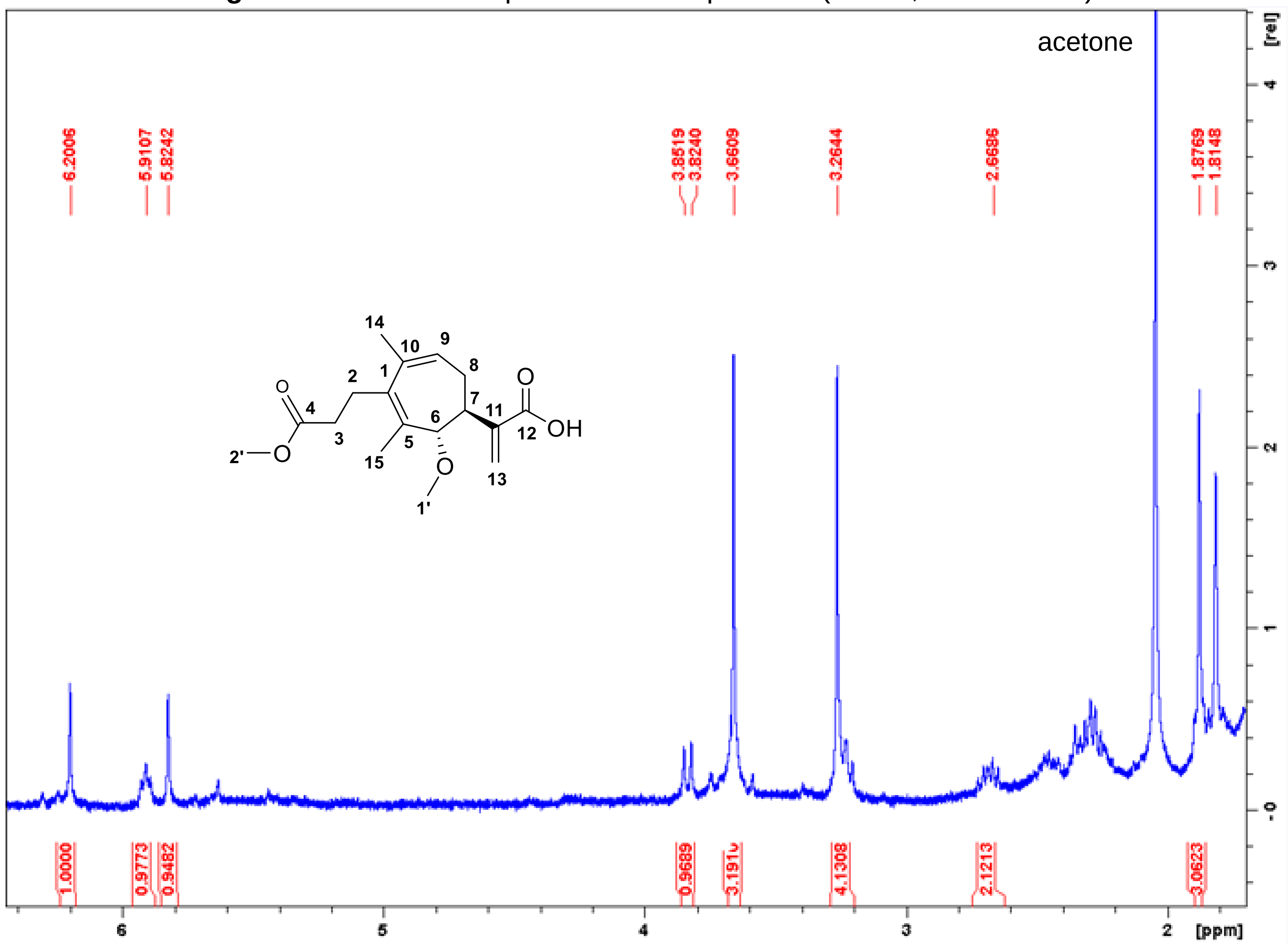

Figure S9-2. COSY spectrum of compound **7** ($CDCl_3$, 400.13 MHz).

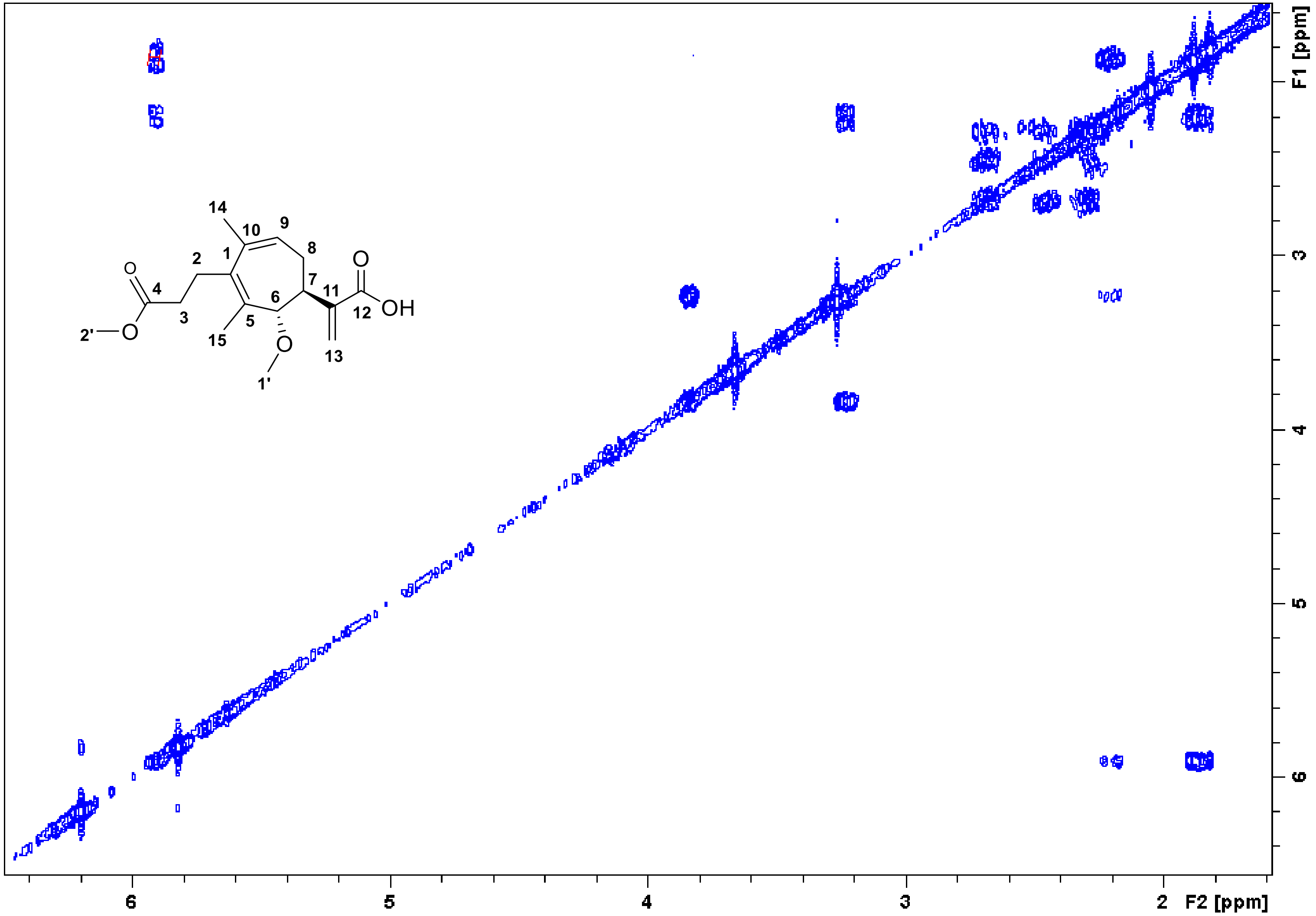

Figure S9-3. HSQC spectrum of compound **7** ($CDCl_3$, 400.13 MHz).

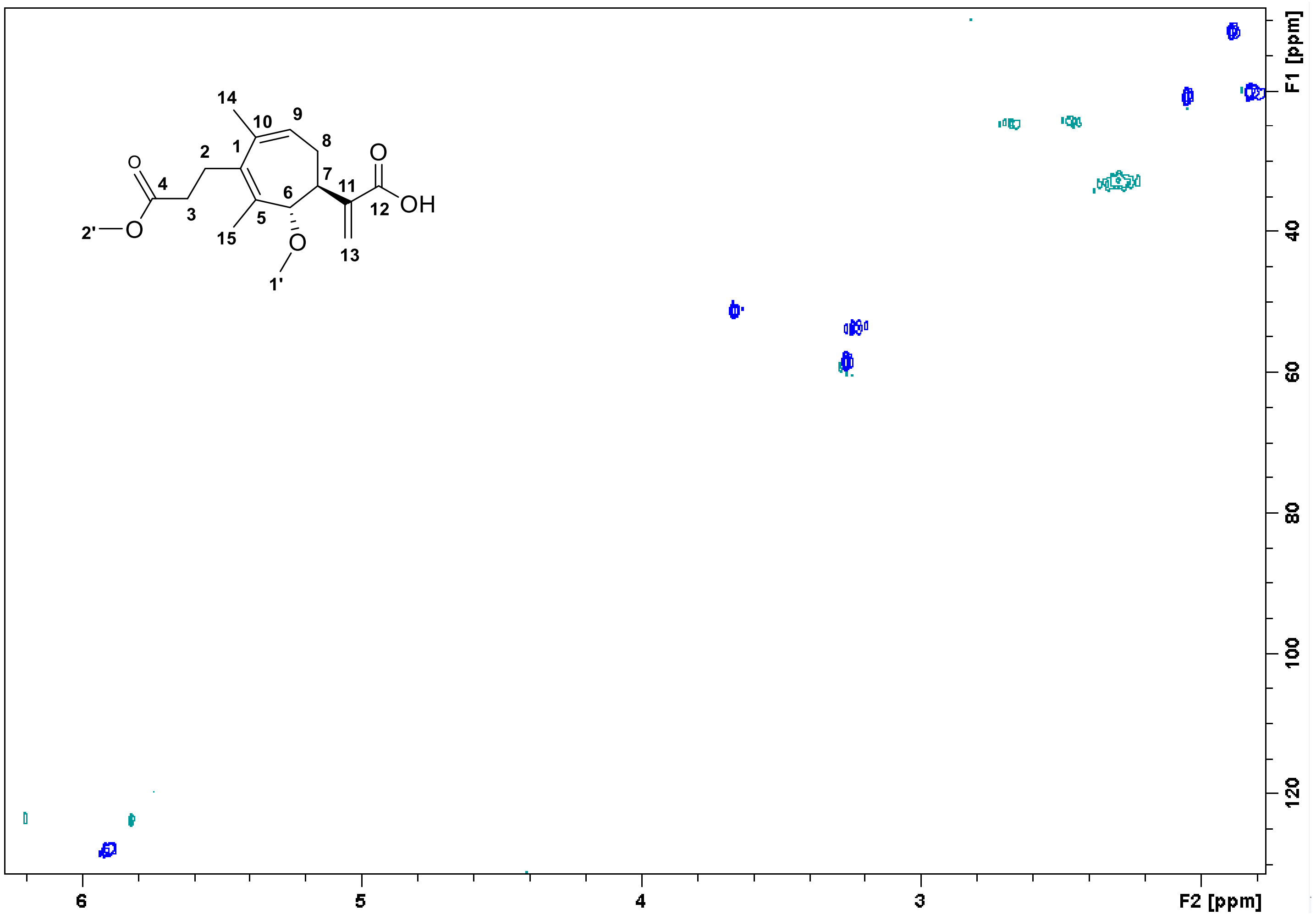

Figure S9-4. HMBC spectrum of compound **7** ($CDCl_3$, 400.13 MHz).

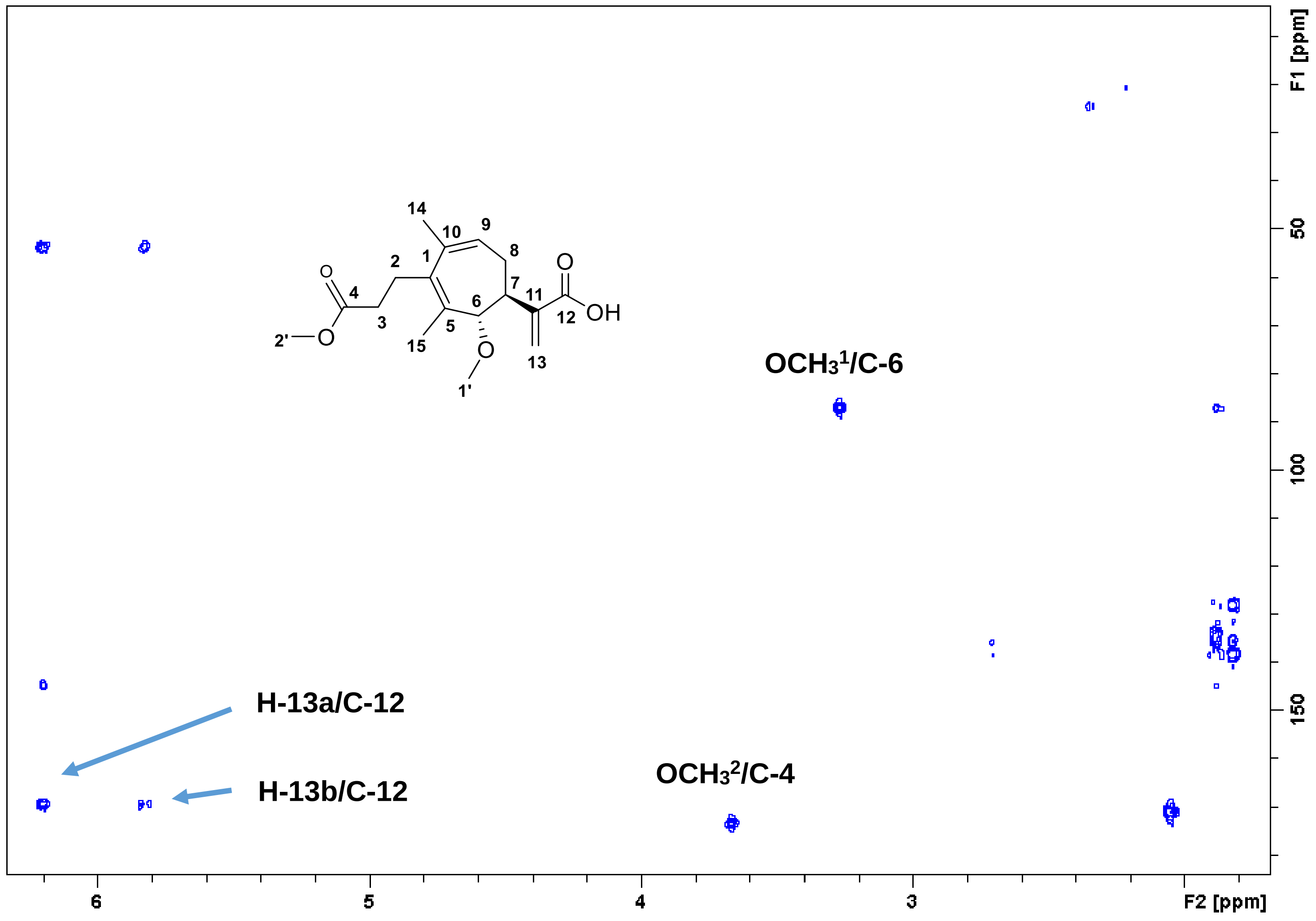

Figure S9-5. HRMS spectrum of compound **7.**

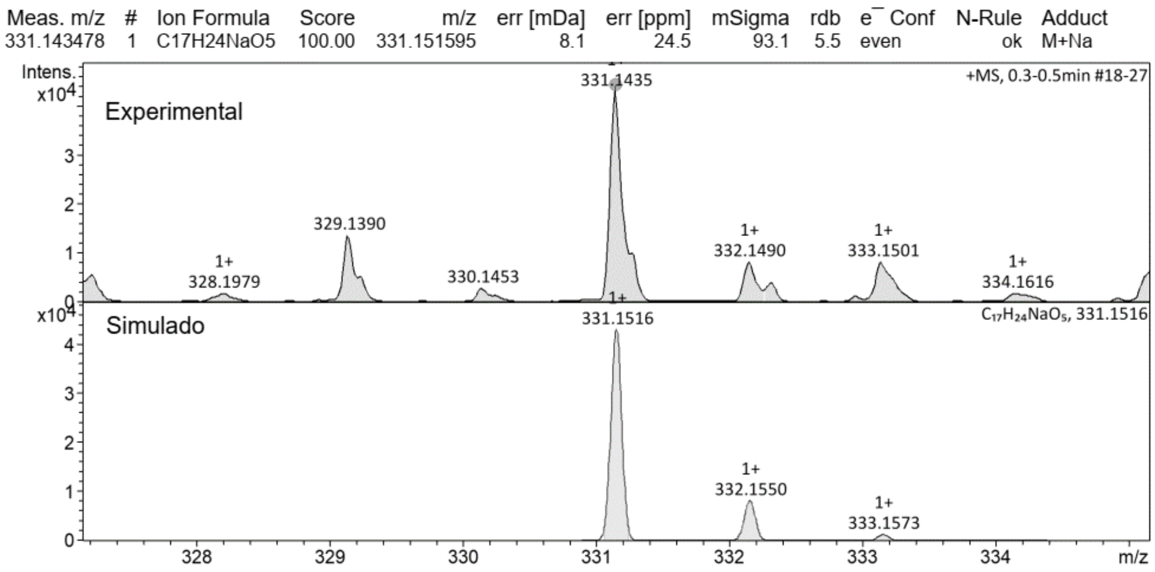

Figure S9-6. IR spectrum of compound **7.**

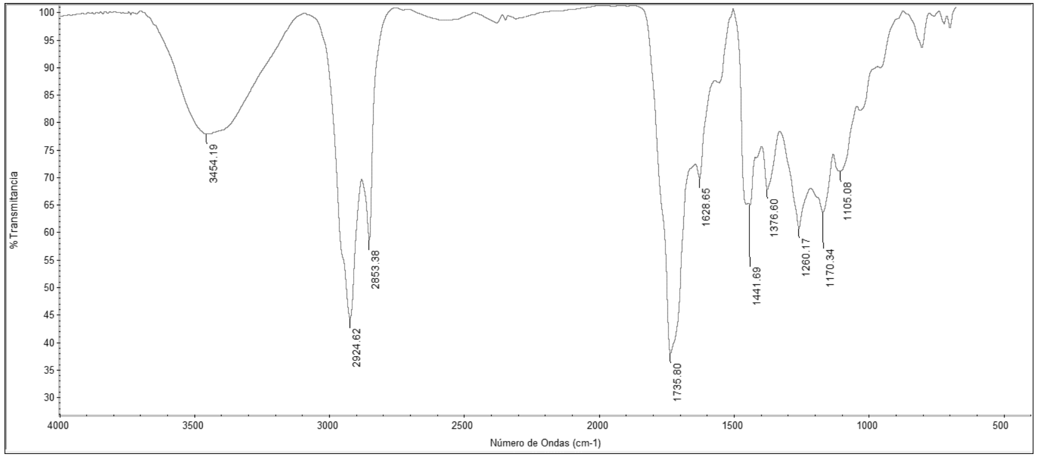

Figure S10-1. ^{1}H NMR spectrum of compound **8** ($CDCl_3$, 400.13 MHz).

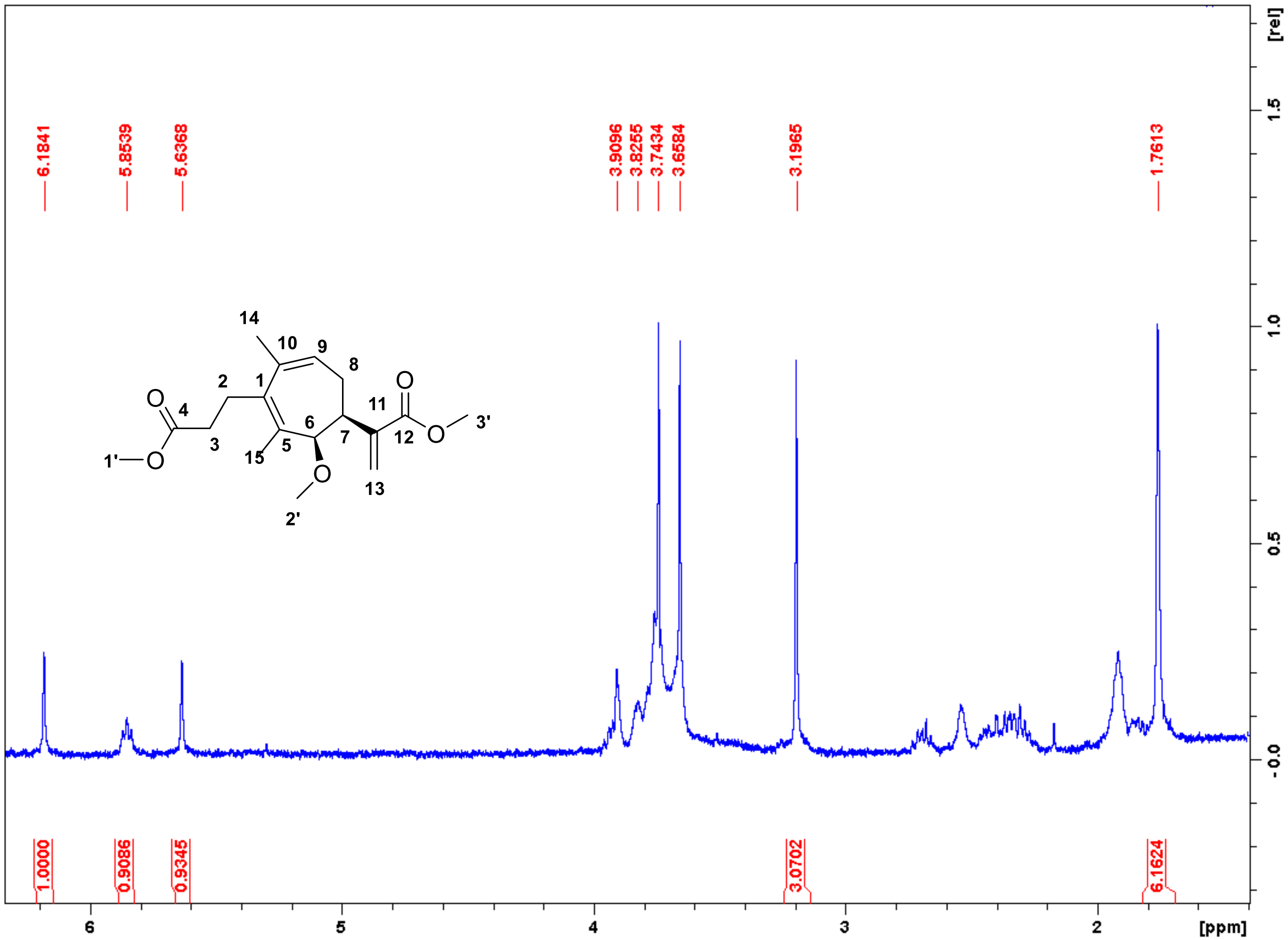

Figure S10-2. COSY spectrum of compound **8** ($CDCl_3$, 400.13 MHz).

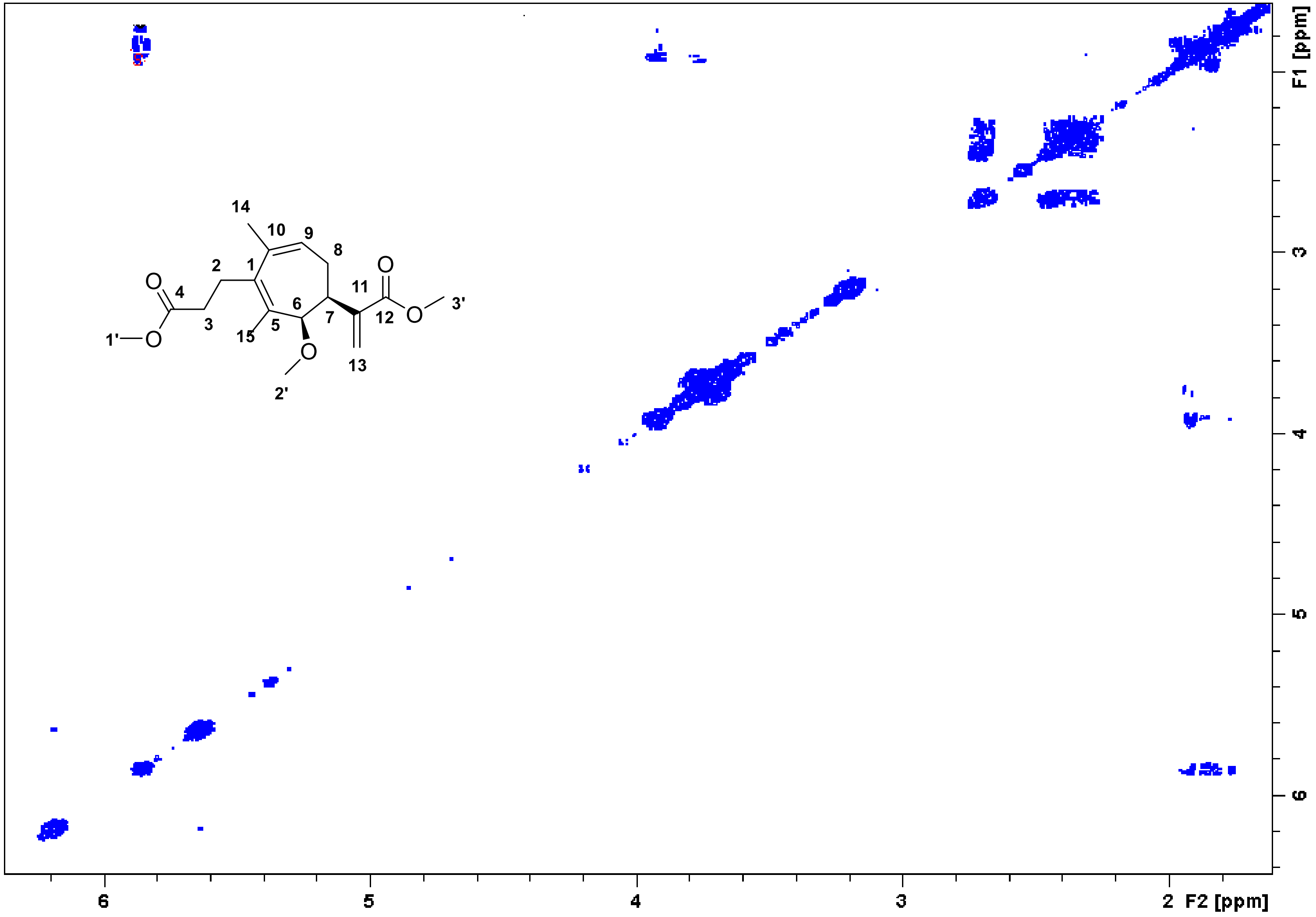

Figure S10-3. HSQC spectrum of compound **8** ($CDCl_3$, 400.13 MHz).

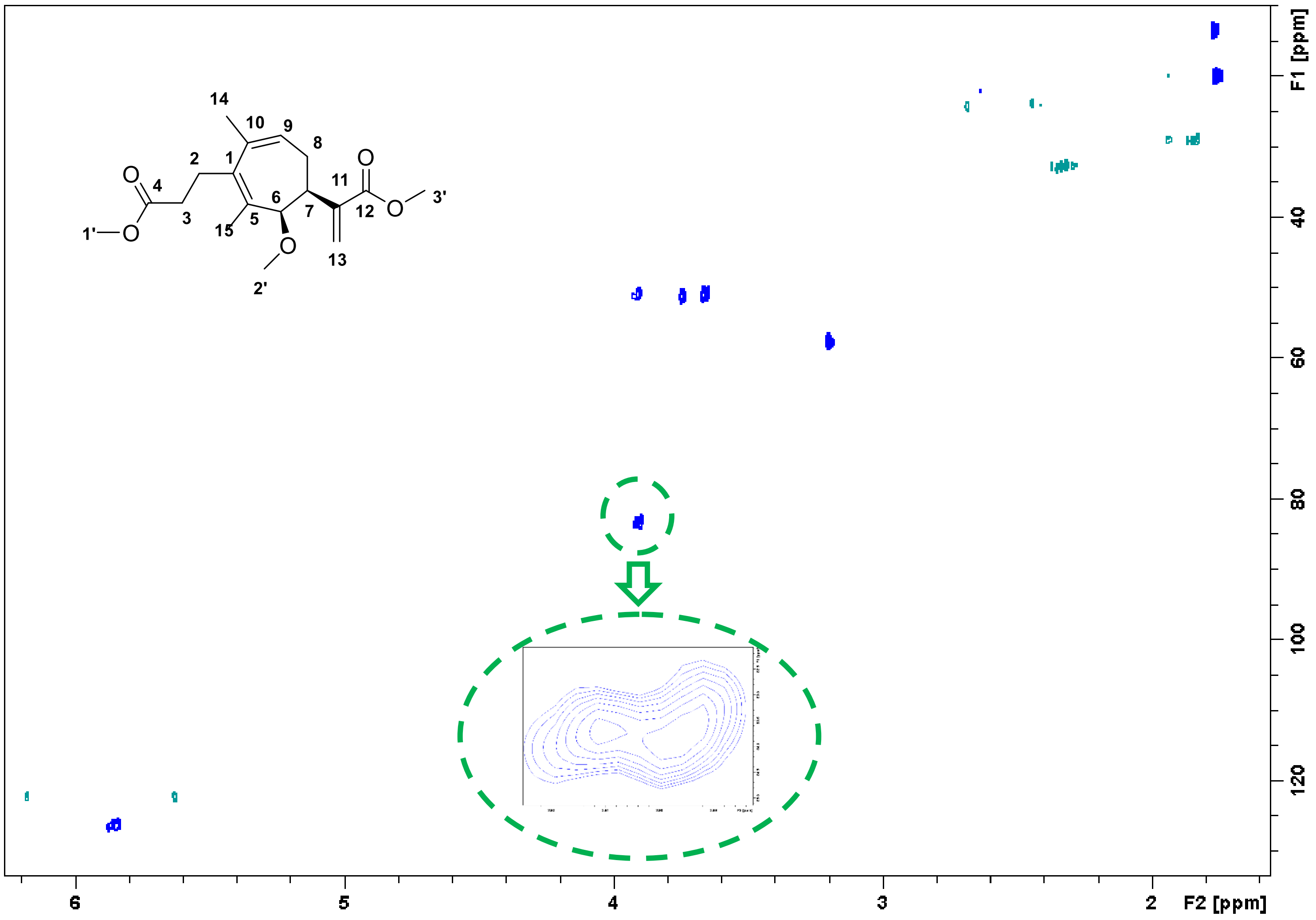

Figure S10-4. HMBC spectrum of compound **8** ($CDCl_3$, 400.13 MHz).

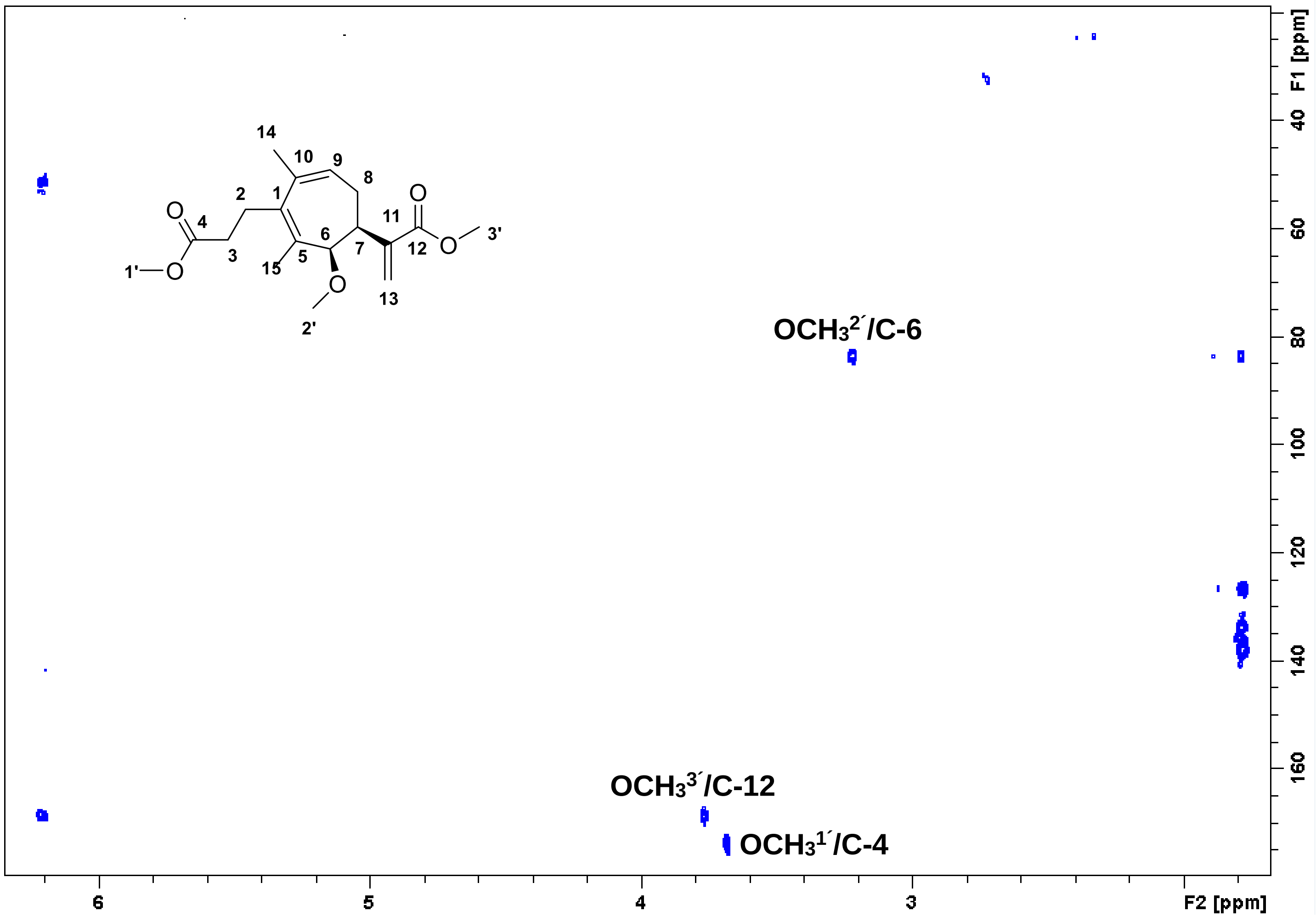

Figure S10-5. HRMS spectrum of compound **8.**

Meas. m/z	#	Ion Formula	Score	m/z	err [mDa]	err [ppm]	mSigma	rdb	e⁻ Conf	N-Rule	Adduct
345.163042	1	C18H26NaO5	100.00	345.167245	4.2	12.2	17.3	5.5	even	ok	M+Na

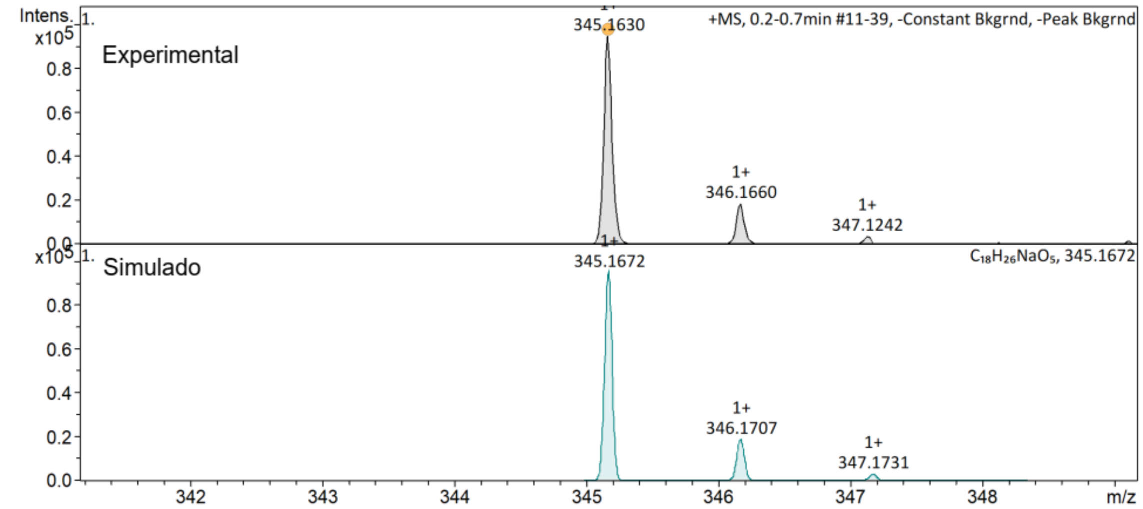

Figure S10-6. IR spectrum of compound **8.**

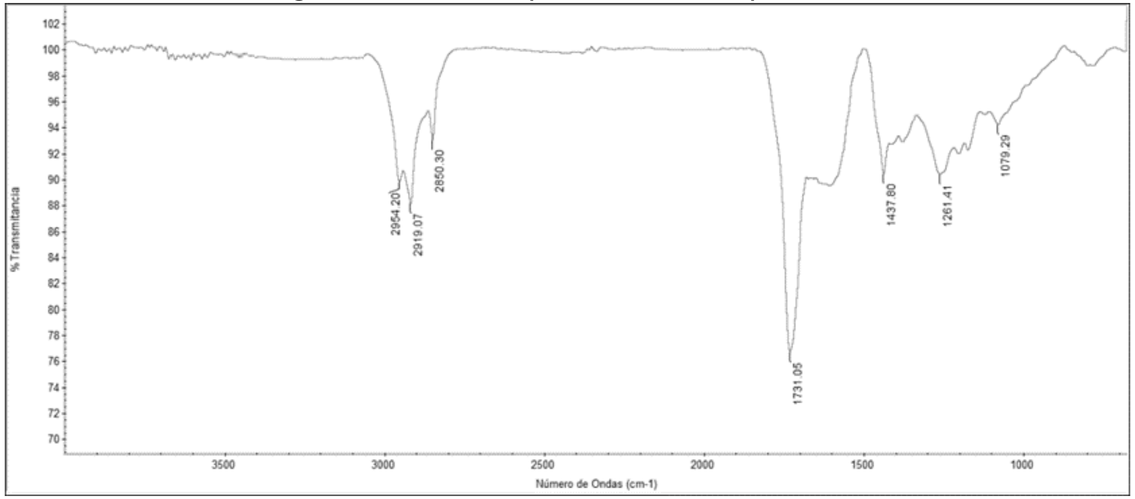

Figure S11-1. ^{1}H NMR spectrum of compound **9** ($CDCl_3$, 400.13 MHz).

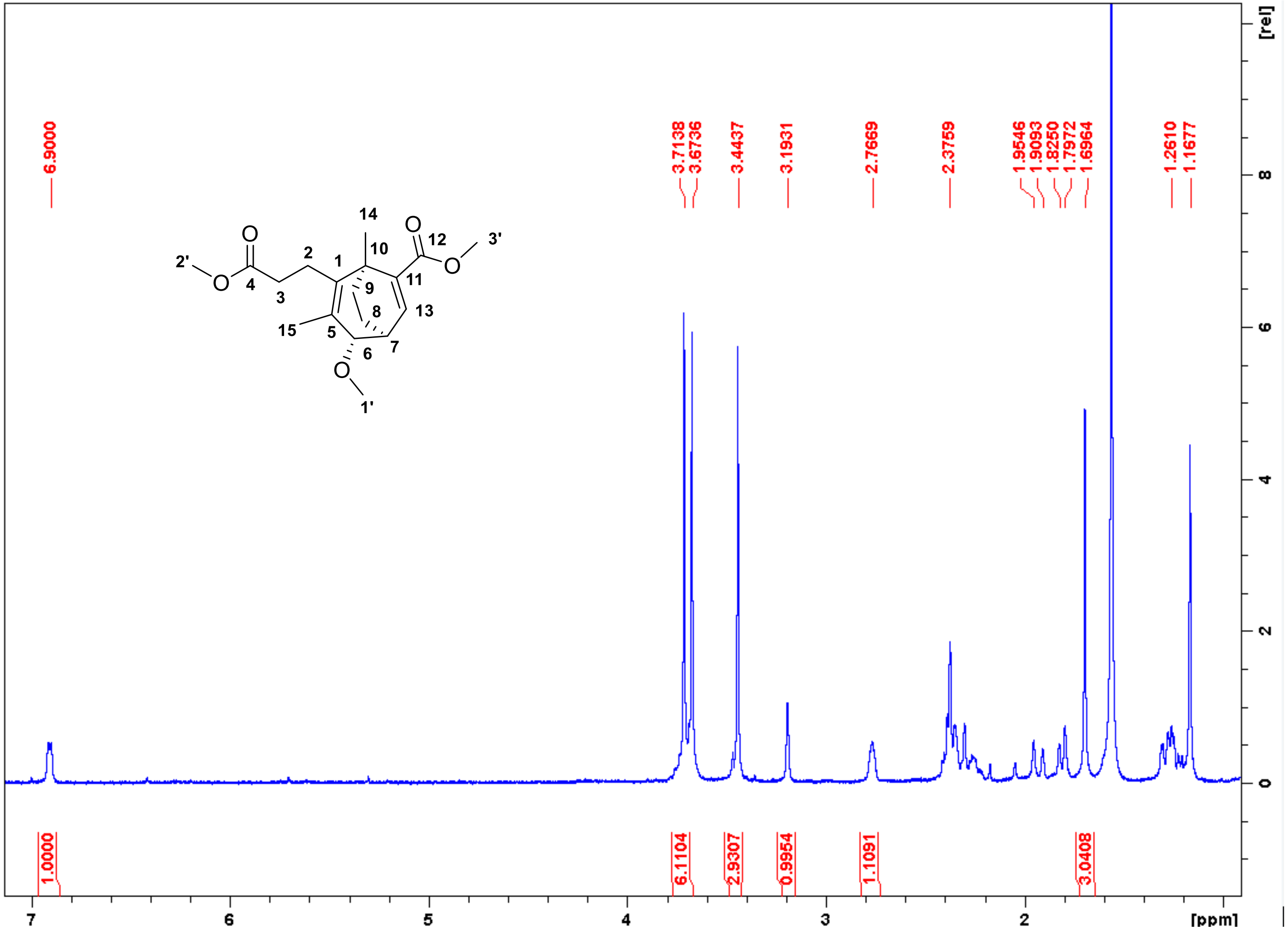

Figure S11-2. COSY spectrum of compound **9** ($CDCl_3$, 400.13 MHz).

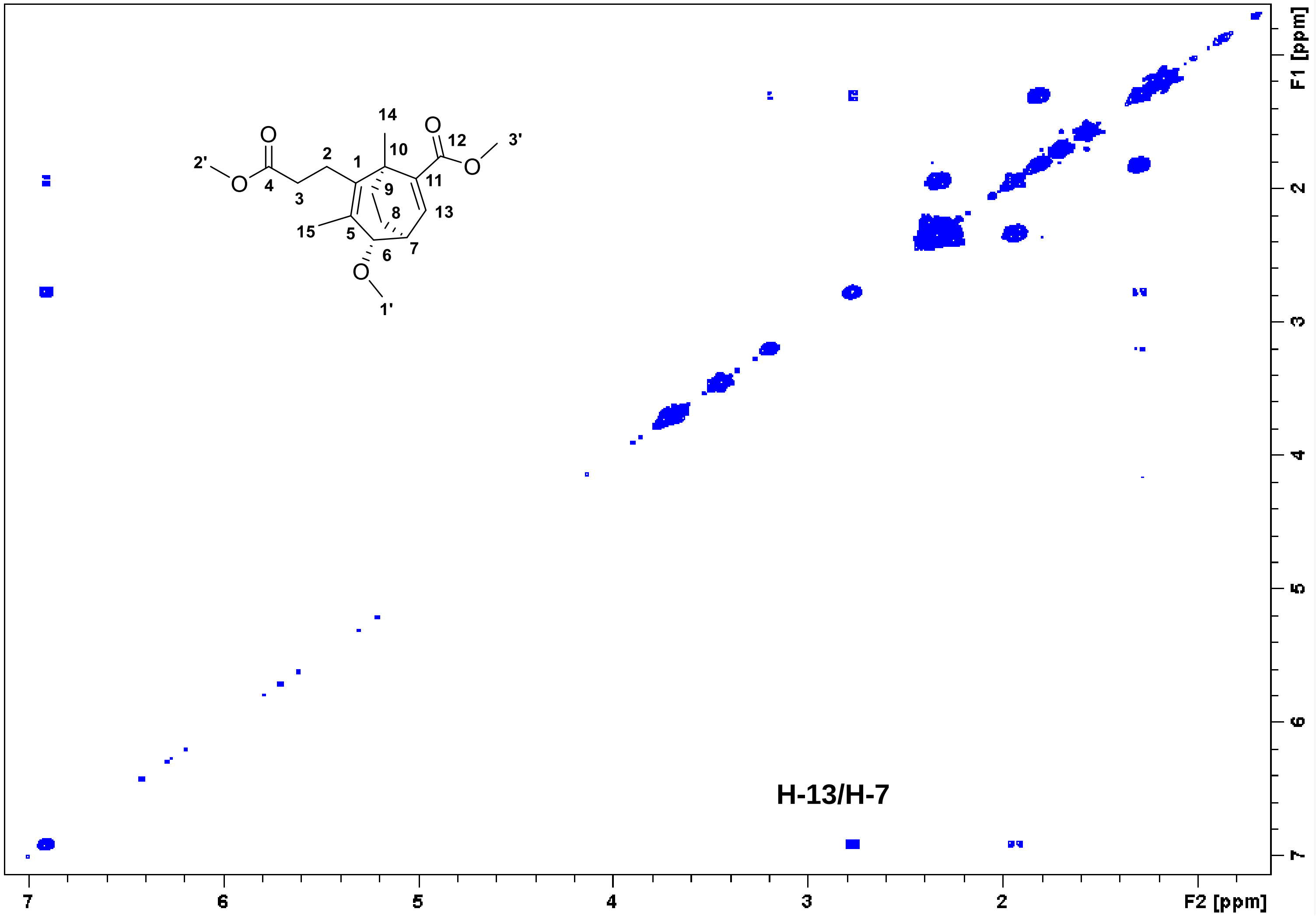

Figure S11-3. HSQC spectrum of compound **9** ($CDCl_3$, 400.13 MHz).

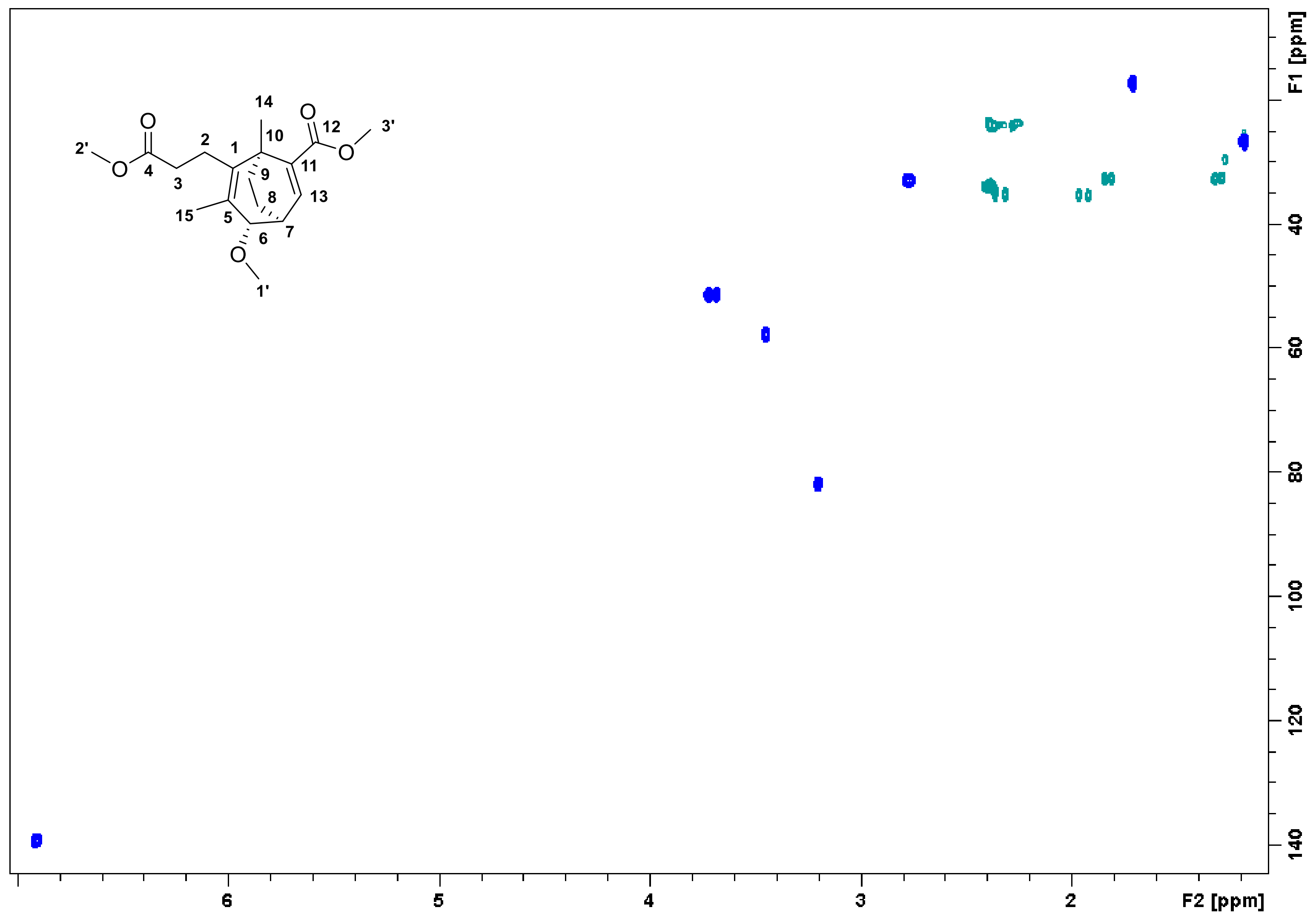

F1 [ppm]
F2 [ppm]
40
60
80
100
120
140
6
5
4
3
2
14
10
12
3'
2'
2
1
9
11
4
3
8
13
15
5
6
7
1'

Figure S11-4. HMBC spectrum of compound **9** ($CDCl_3$, 400.13 MHz).

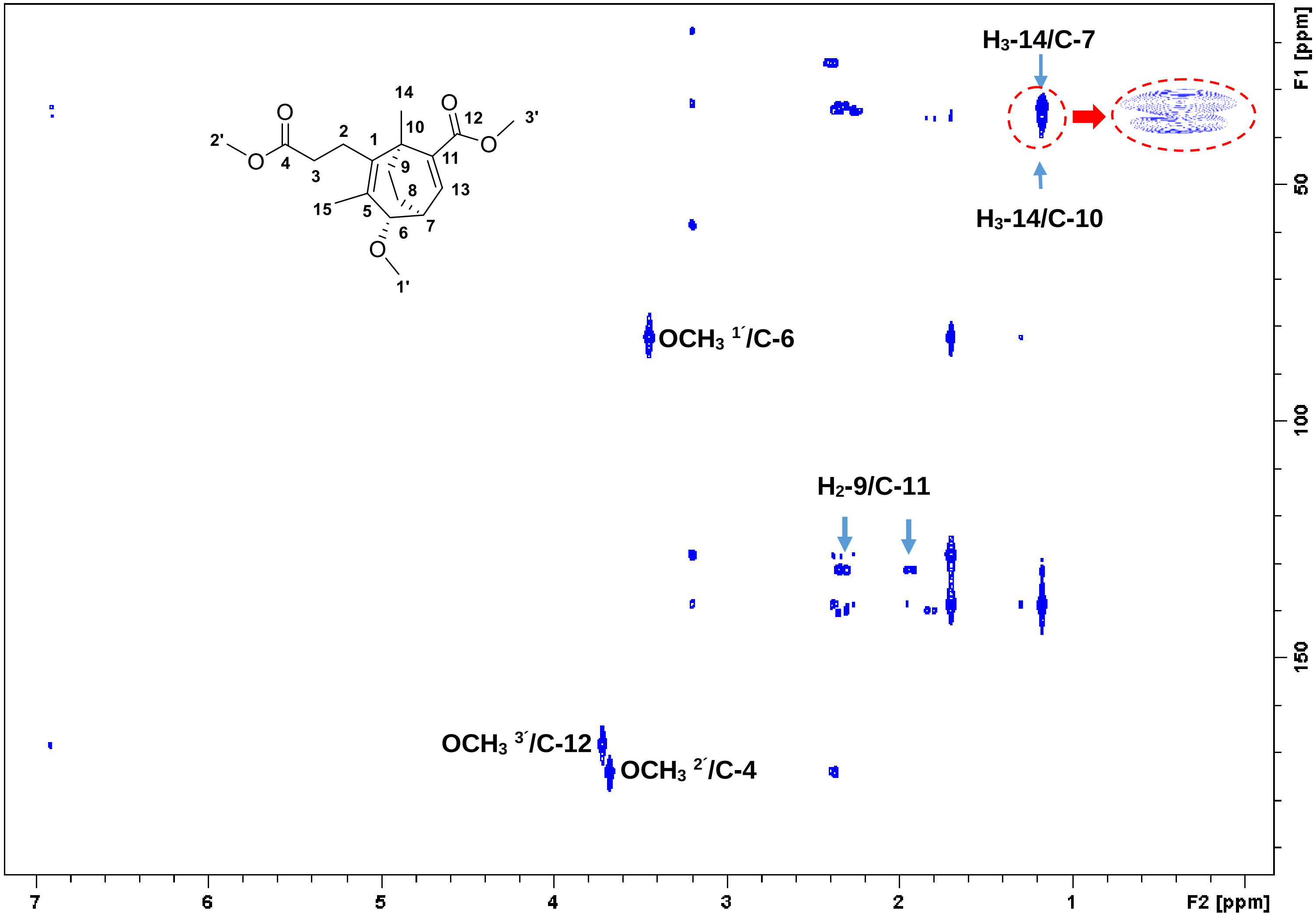

Figure S11-5. NOESY spectrum of compound **9** ($CDCl_3$, 400.13 MHz).

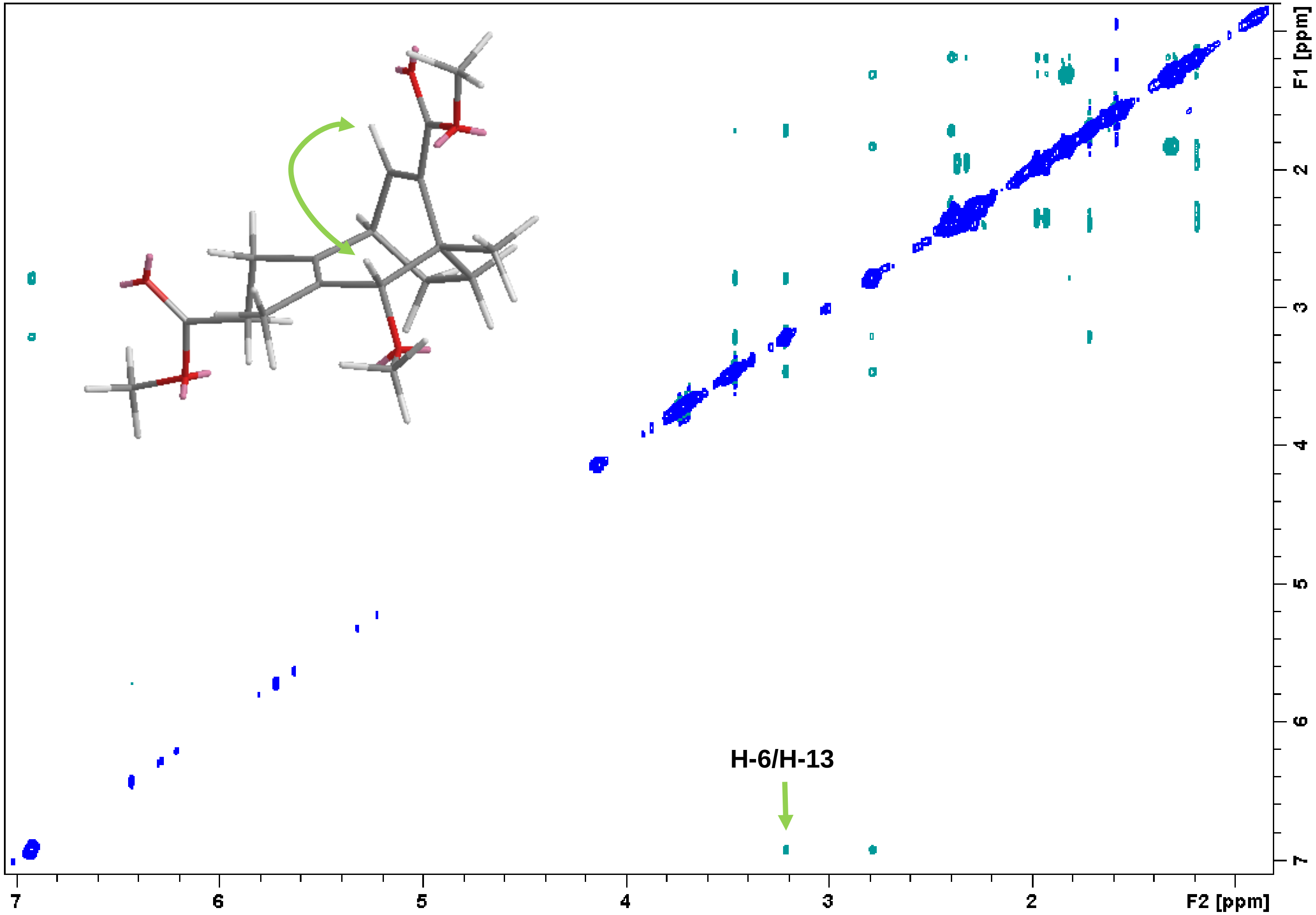

Figure S11-6. HRMS spectrum of compound **9.**

Meas. m/z	#	Ion Formula	Score	m/z	err [mDa]	err [ppm]	mSigma	rdb	e¯ Conf	N-Rule	Adduct
345.163042	1	C18H26NaO5	100.00	345.167245	4.2	12.2	17.3	5.5	even	ok	M+Na

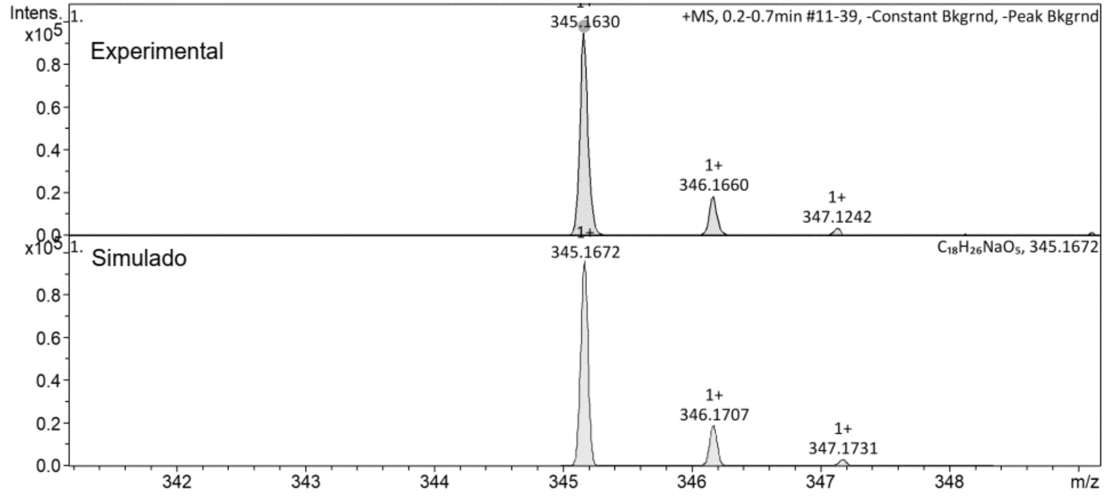

Figure S11-7. IR spectrum of compound **9.**

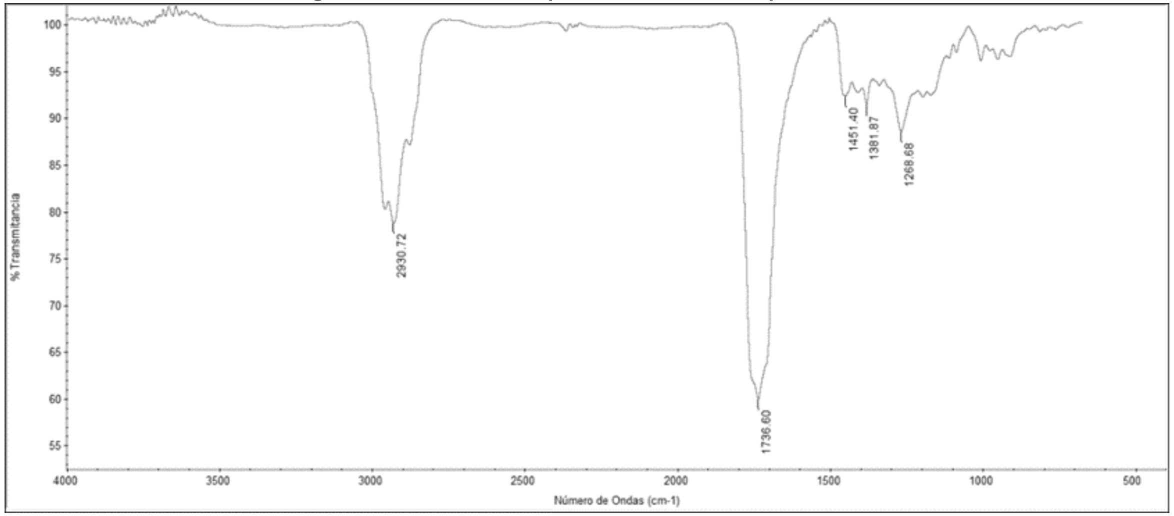

Figure S12-1. ^{1}H NMR spectrum of compound **10** ($CDCl_3$, 400.13 MHz).

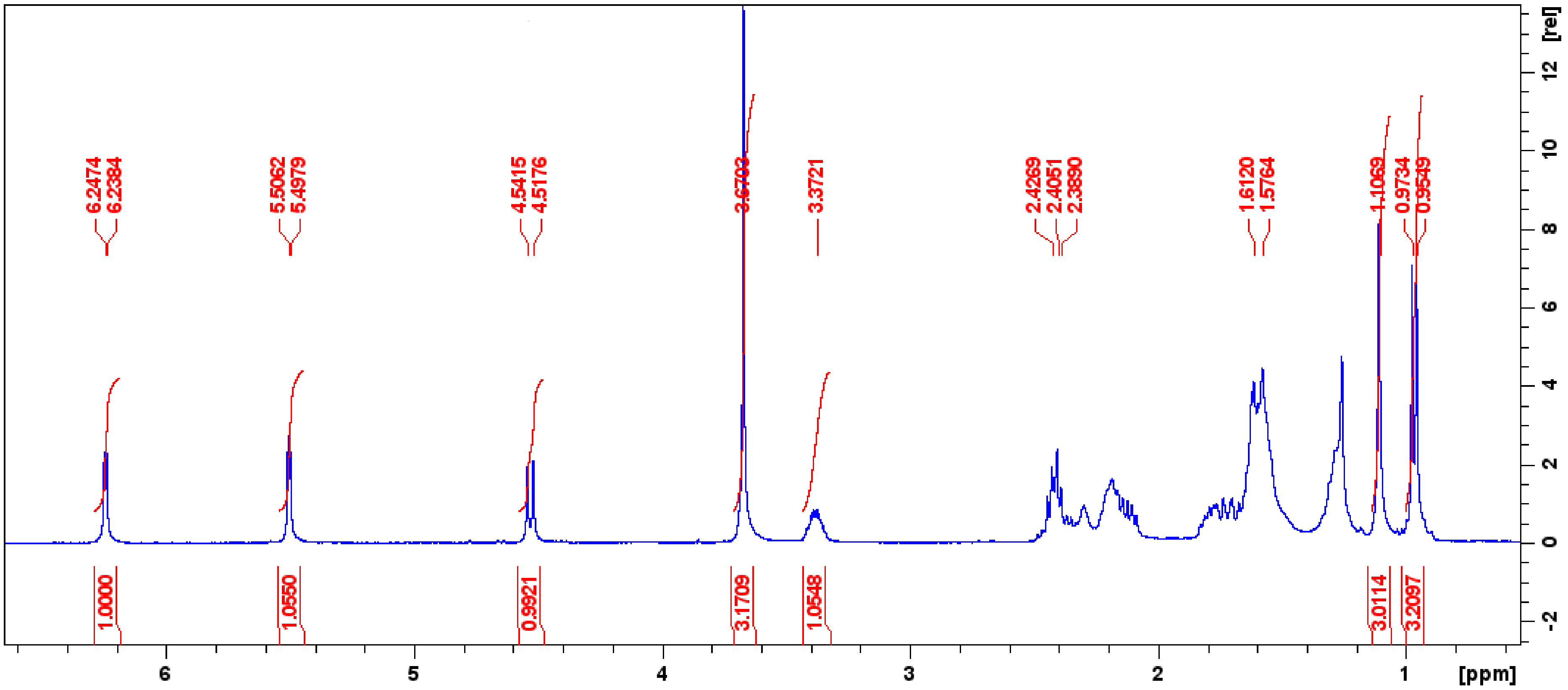

Figure S13-1. ^{1}H NMR spectrum of compound **11** ($CDCl_3$, 400.13 MHz).

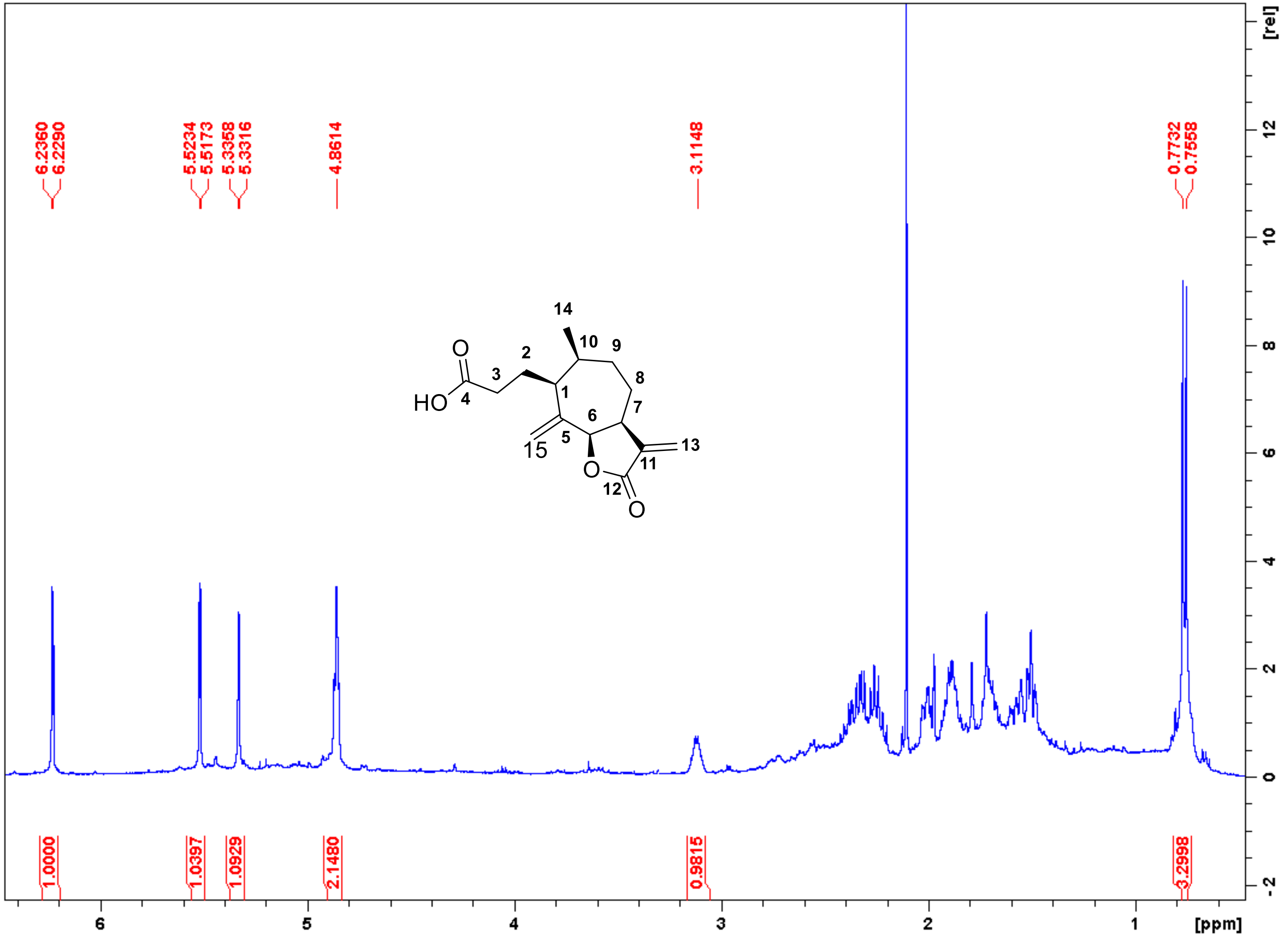

Figure S13-2. ^{13}C NMR spectrum of compound **11** ($CDCl_3$, 100.03 MHz).

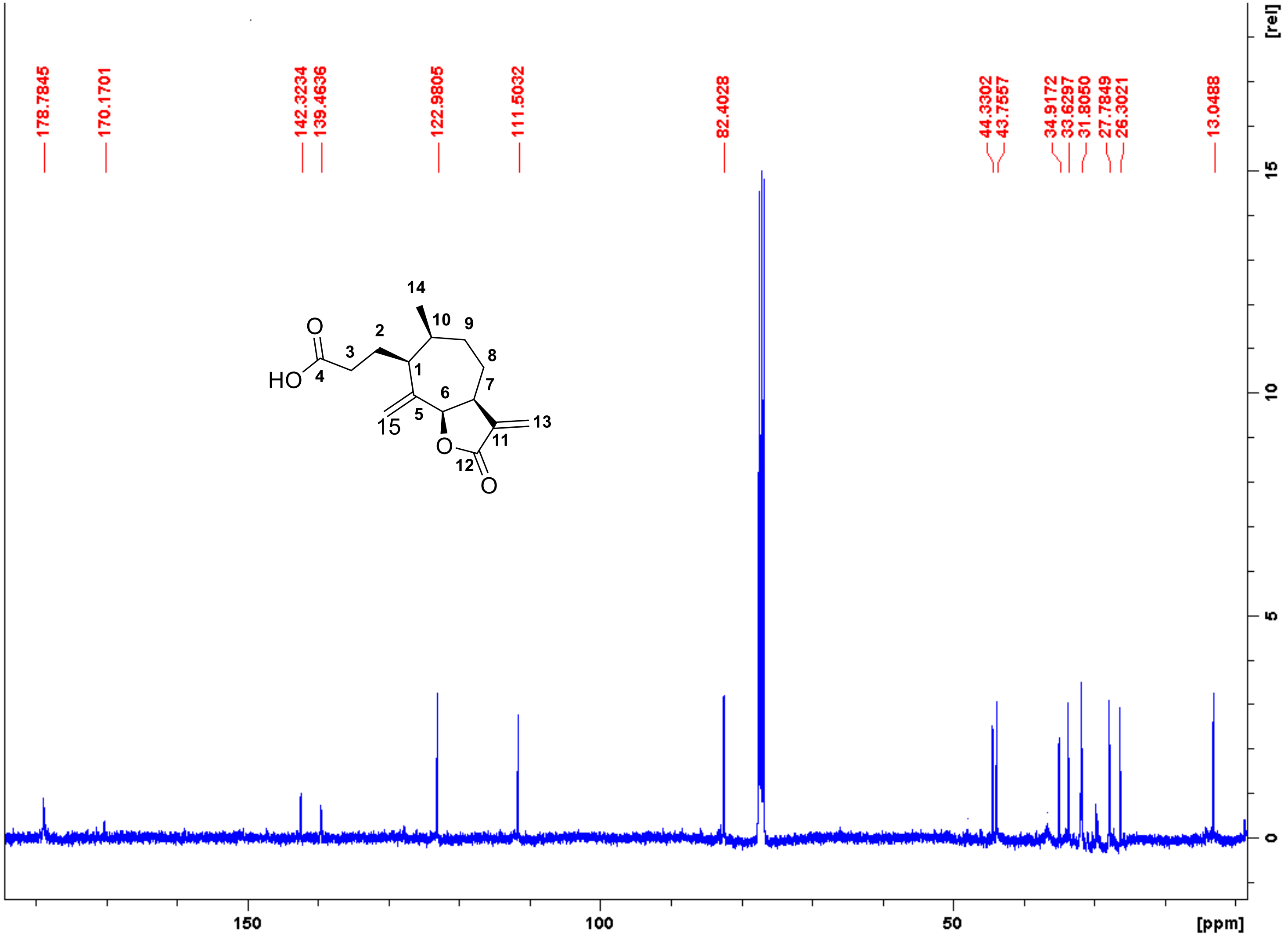

Figure S13-3. COSY spectrum of compound **11** ($CDCl_3$, 400.13 MHz).

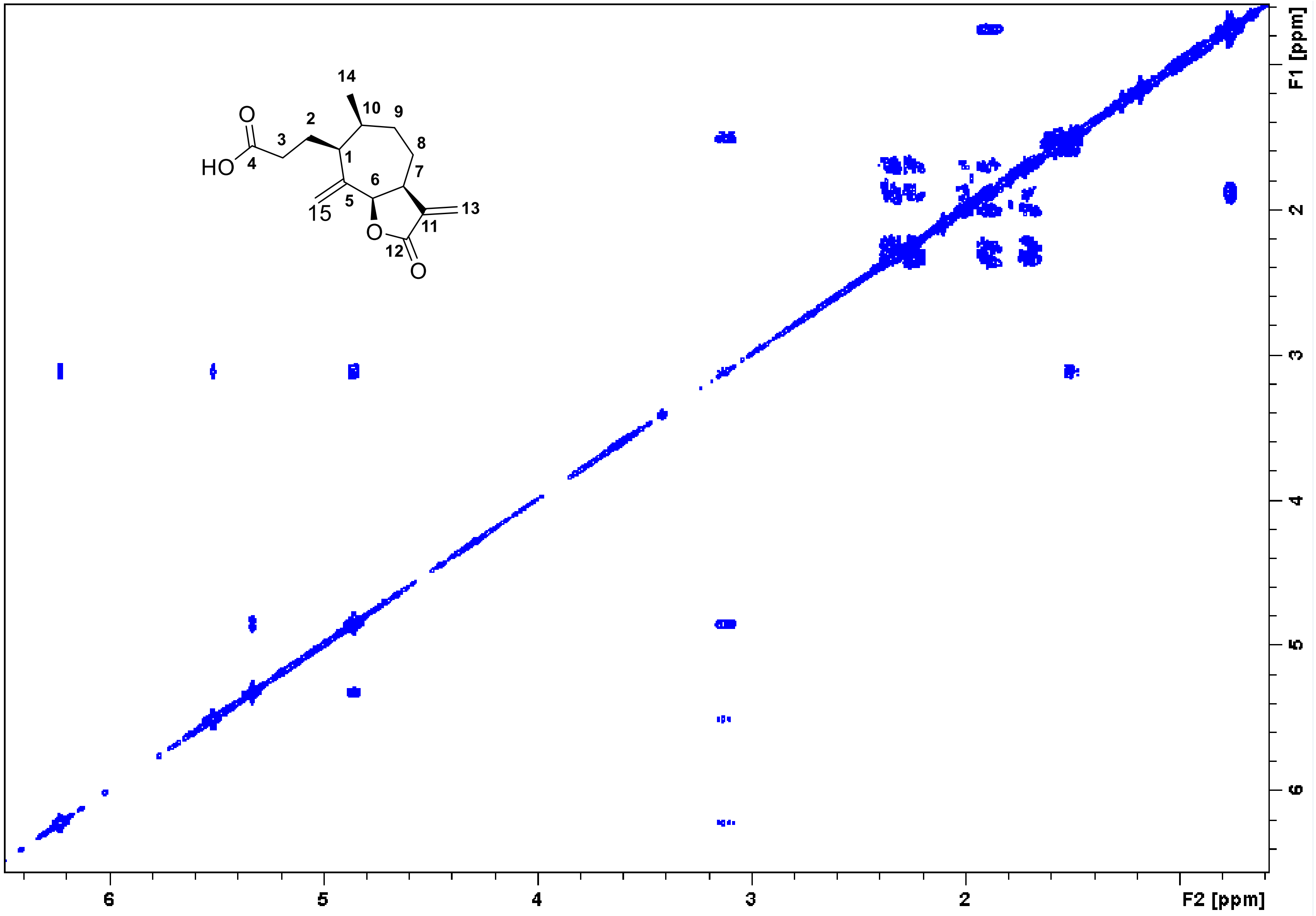

Figure S13-4. HSQC spectrum of compound **11** ($CDCl_3$, 400.13 MHz).

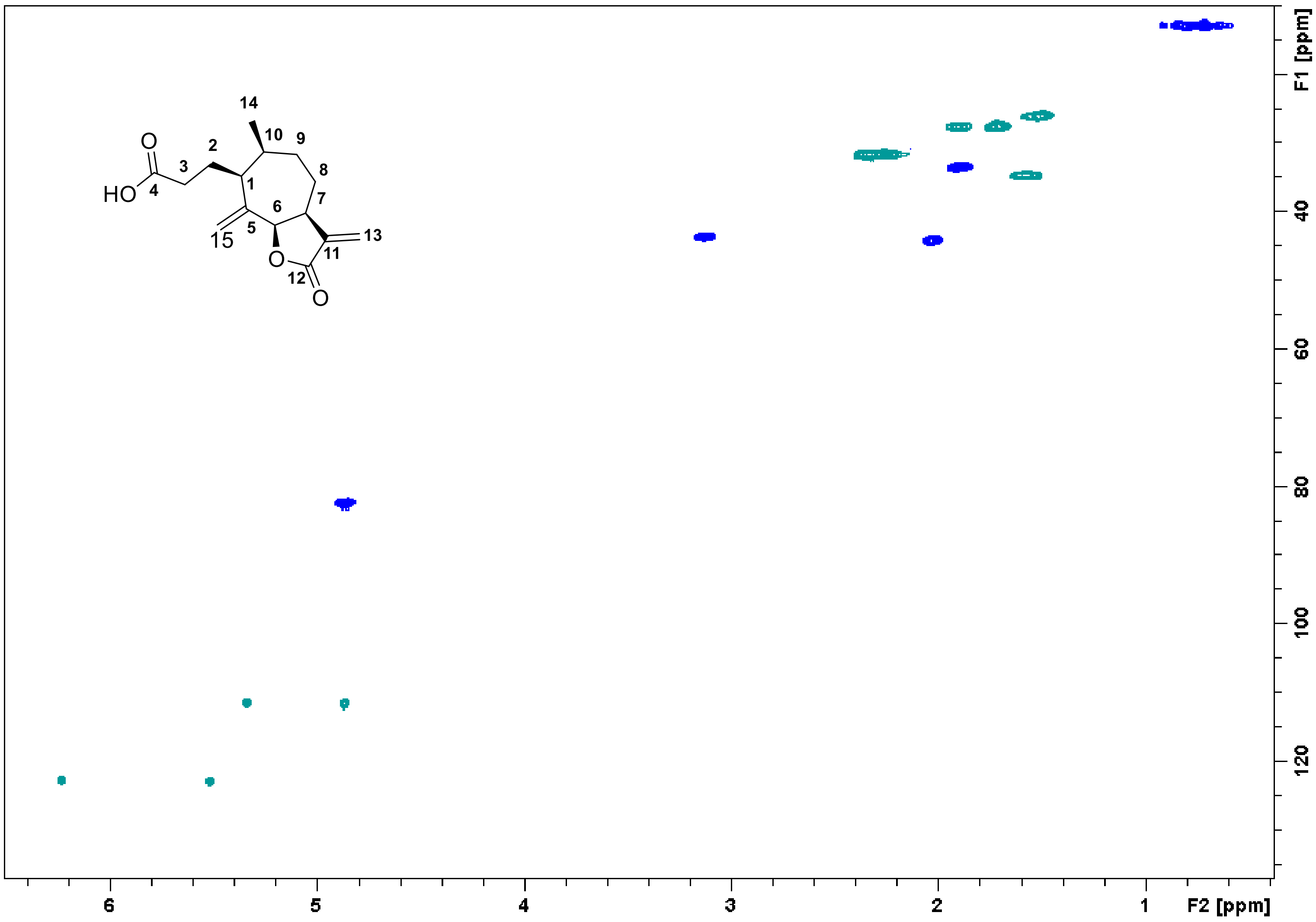

Figure S13-5. HMBC spectrum of compound **11** ($CDCl_3$, 400.13 MHz).

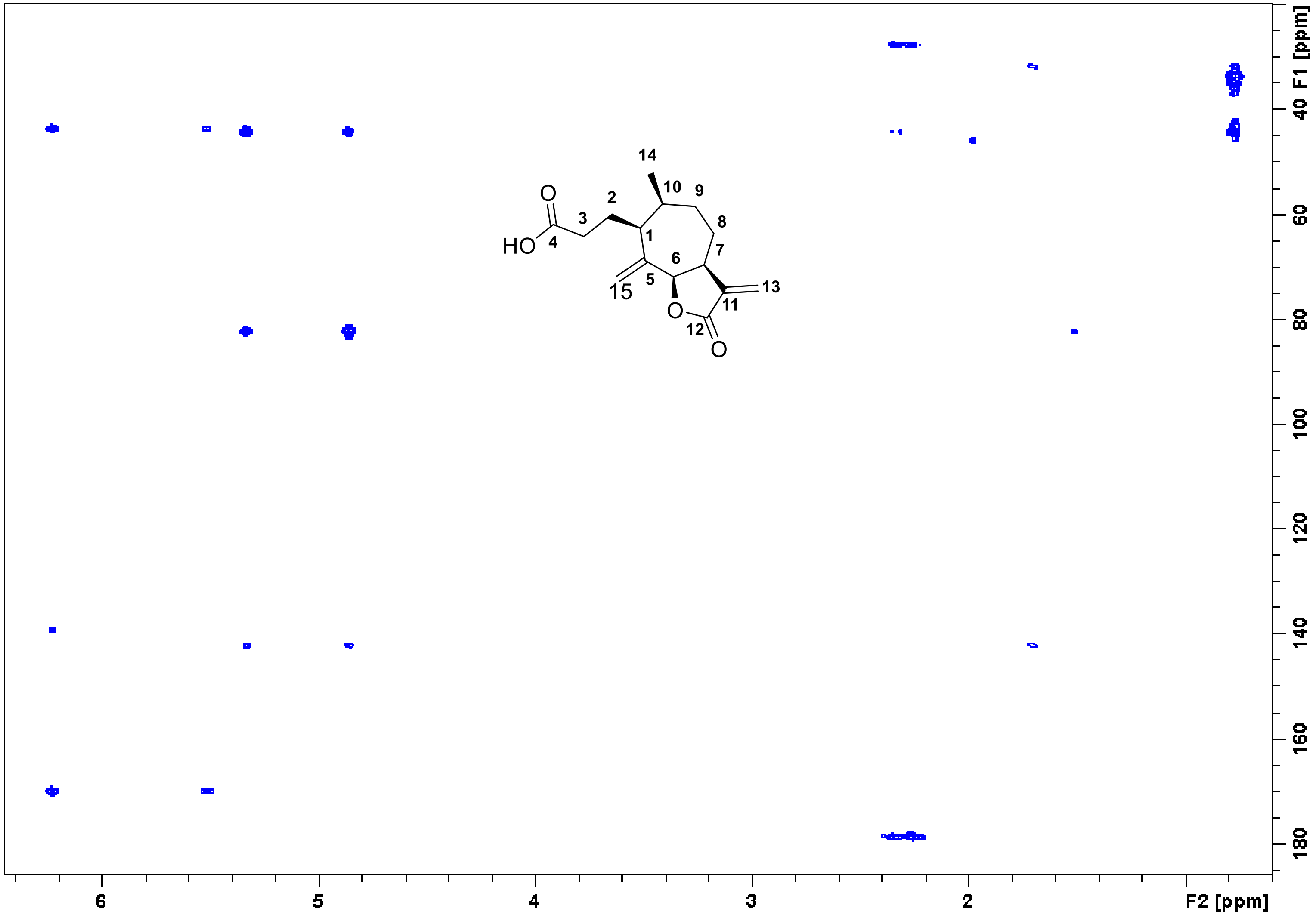

Figure S13-6. HRMS spectrum of compound **11.**

Meas. m/z	#	Ion Formula	Score	m/z	err [mDa]	err [ppm]	mSigma	rdb	e¯ Conf	N-Rule	Adduct
287.125002	1	C15H20NaO4	100.00	287.125380	-0.4	-1.3	230.2	5.5	even	ok	M+Na

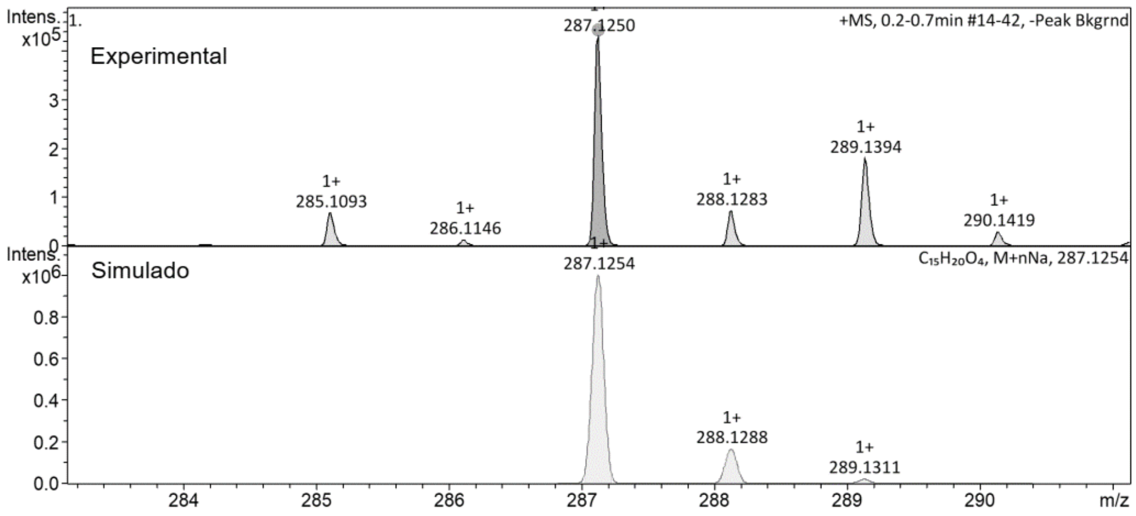

Figure S13-7. IR spectrum of compound **11.**

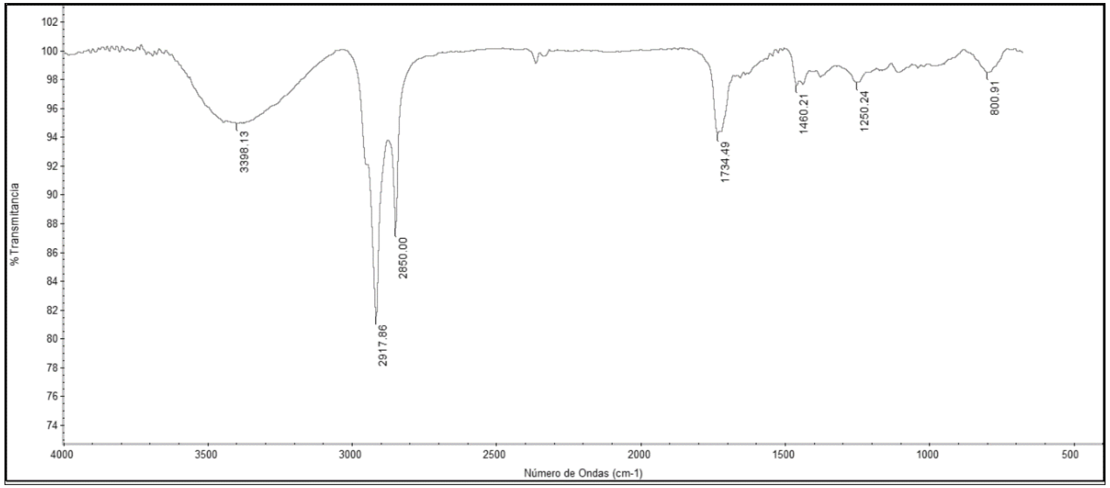

Table S1. Druglikeness parameters. The colored zone is the suitable physicochemical space for oral bioavalability (LIPO Lipophility): -0.7 < XLOGP3 < +5.0; SIZE: 150 g/mol < MV < 500 g/mol; POLAR (Polarity): 20Å < TPSA < 130 Å; INSOLU Insolubility): -6 < Log S (ESOL) < 0; INSATU (Insaturation): 0.25 < Fraction Csp3 < 1; FLEX (Flexibility): 0 < Num. rotatable bonds < 9).

PsiA

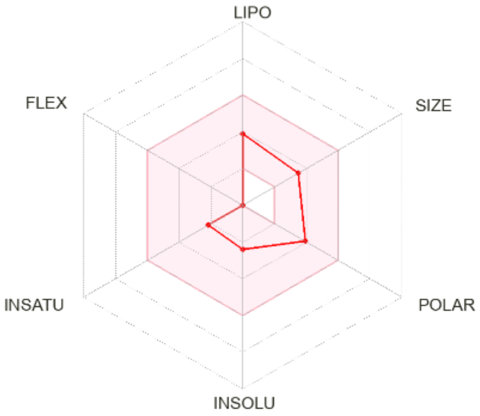

PsiB

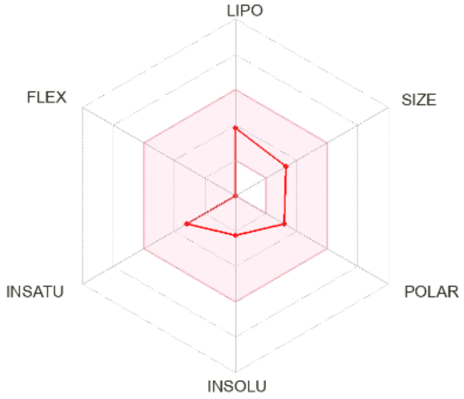

PsiC

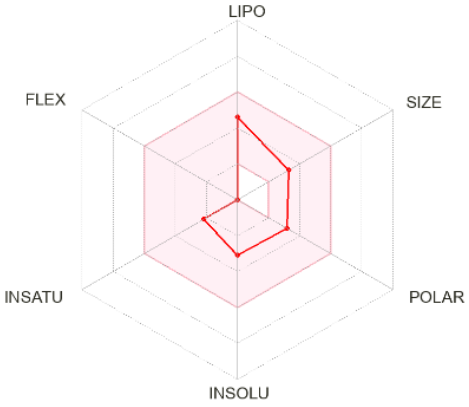

1

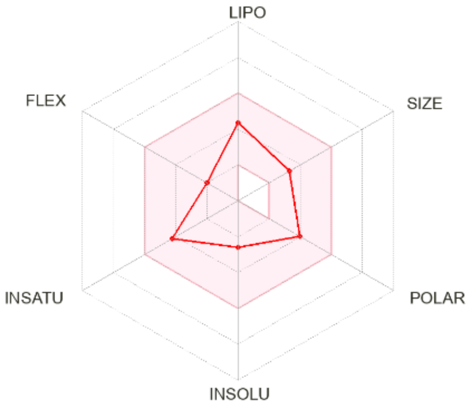

2

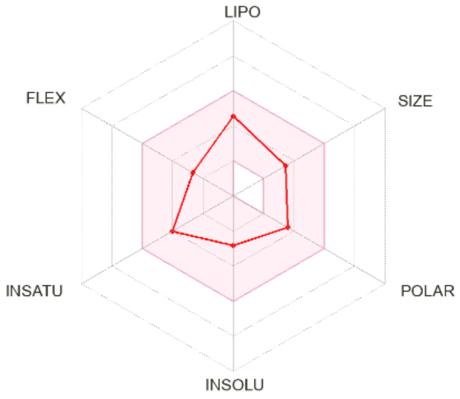

3

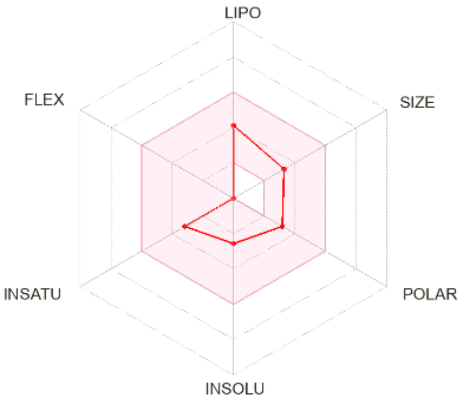

4

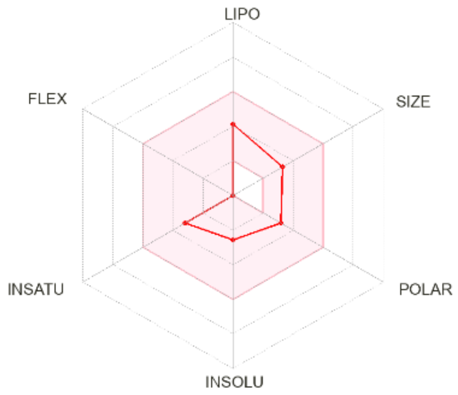
LIPO
SIZE
POLAR
INSOLU
INSATU
FLEX

5

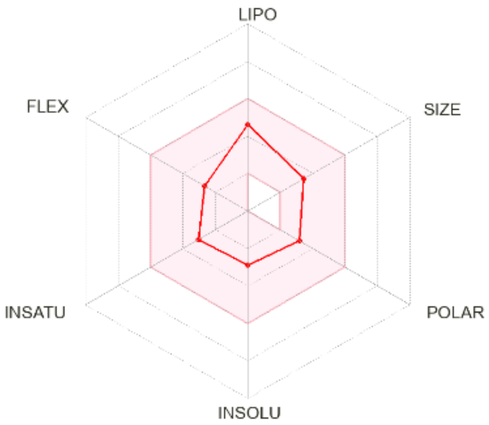
LIPO
SIZE
POLAR
INSOLU
INSATU
FLEX

6

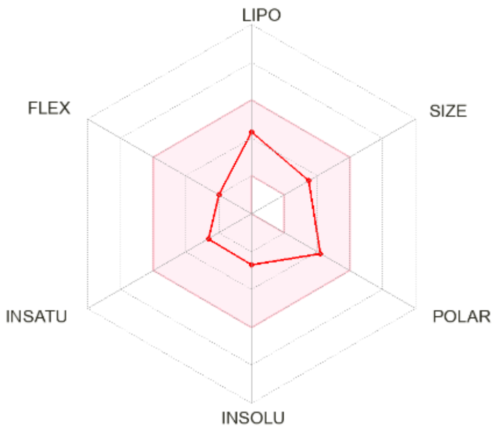
LIPO
SIZE
POLAR
INSOLU
INSATU
FLEX

7

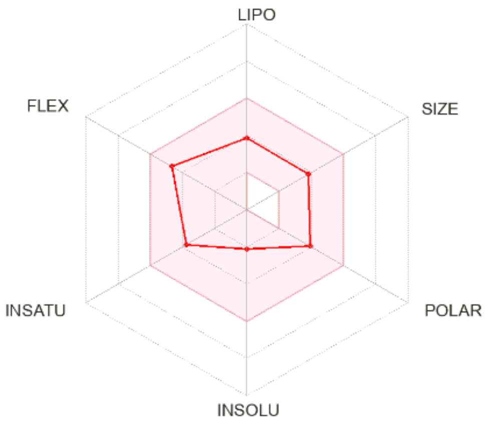
LIPO
SIZE
POLAR
INSOLU
INSATU
FLEX

8

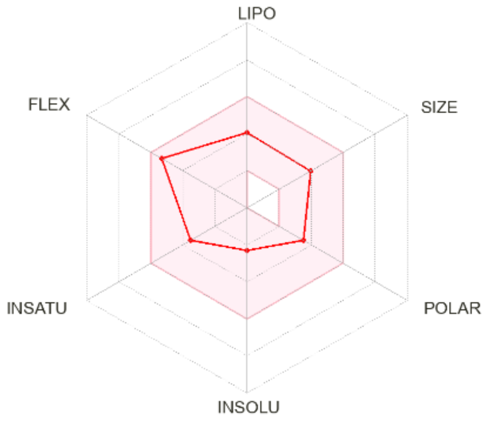
LIPO
SIZE
POLAR
INSOLU
INSATU
FLEX

9

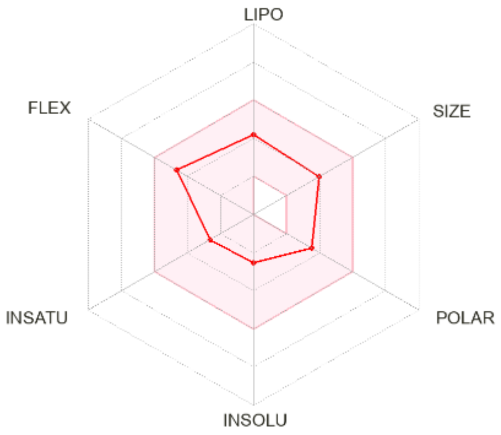
LIPO
SIZE
POLAR
INSOLU
INSATU
FLEX

10

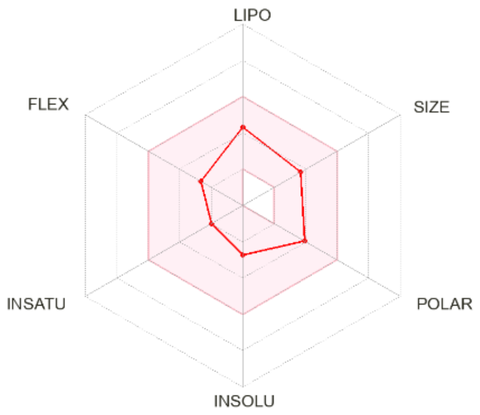
LIPO
SIZE
POLAR
INSOLU
INSATU
FLEX

11

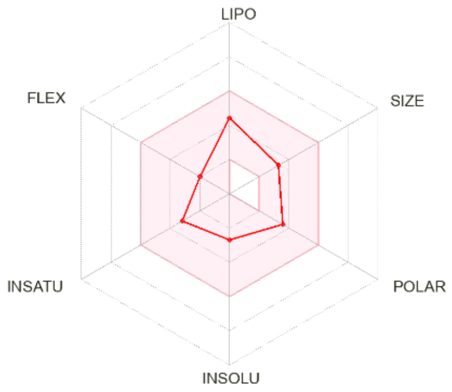
LIPO
SIZE
POLAR
INSOLU
INSATU
FLEX

Table S2. Physicochemical properties of the set of compounds obtained.

	PsiA	PsiB	PsiC	1	2	3	4	5	6	7	8	9	10	11
**Physicochemical Properties**														
Formula	$C_{15}H_{20}O_5$	$C_{15}H_{18}O_4$	$C_{15}H_{20}O_4$	$C_{15}H_{18}O_4$	$C_{16}H_{20}O_4$	$C_{15}H_{18}O_4$	$C_{15}H_{18}O_4$	$C_{16}H_{22}O_4$	$C_{15}H_{20}O_5$	$C_{17}H_{24}O_5$	$C_{18}H_{26}O_5$	$C_{18}H_{26}O_5$	$C_{16}H_{24}O_5$	$C_{15}H_{20}O_4$
Molecular weight	280.32 g/mol	262.30 g/mol	264.32 g/mol	262.30 g/mol	276.33 g/mol	262.30 g/mol	262.30 g/mol	278.34 g/mol	280.32 g/mol	308.37 g/mol	322.40 g/mol	322.40 g/mol	296.36 g/mol	264.32 g/mol
Num. heavy atoms	20	19	19	19	20	19	19	20	20	22	23	23	21	19
Fraction Csp3	0.73	0.60	0.73	0.47	0.50	0.60	0.60	0.62	0.67	0.53	0.56	0.67	0.75	0.60
Num. rotatable bonds	0	0	0	3	4	0	0	4	3	7	8	7	4	3
Num. H-bond acceptors	5	4	4	4	4	4	4	4	5	5	5	5	5	4
Num. H-bond donors	1	0	0	2	1	0	0	0	1	1	0	0	1	1
Molar Refractivity	71.21	69.54	70.01	71.85	76.17	69.54	69.54	76.42	72.51	84.44	88.76	86.86	78.09	72.10
TPSA	72.83 Å^2	52.60 Å^2	52.60 Å^2	74.60 Å^2	63.60 Å^2	52.60 Å^2	52.60 Å^2	52.60 Å^2	80.67 Å^2	72.83 Å^2	61.83 Å^2	61.83 Å^2	72.83 Å^2	63.60 Å^2
**Lipophilicity**														
Consensus Log Po/w	1.69	2.18	2.37	2.22	2.58	2.27	2.24	2.77	1.88	2.32	2.71	2.70	2.12	2.34
**Water Solubility**														
Log S (ESOL)	-2.40	-2.23	-3.08	-2.60	-2.83	-2.56	-2.56	-2.90	-2.68	-2.10	-2.32	-2.52	-2.72	-2.68
Class	Soluble	Soluble	Soluble	Soluble	Soluble	Soluble	Soluble	Soluble	Soluble	Soluble	Soluble	Soluble	Soluble	Soluble
**Druglikeness**														
Lipinski	Yes; 0 violation	Yes; 0 violation	Yes; 0 violation	Yes; 0 violation	Yes; 0 violation	Yes; 0 violation	Yes; 0 violation	Yes; 0 violation	Yes; 0 violation	Yes; 0 violation	Yes; 0 violation	Yes; 0 violation	Yes; 0 violation	Yes; 0 violation
**Medicinal Chemistry**														
PAINS	0 alert	0 alert	0 alert	0 alert	0 alert	0 alert	0 alert	0 alert	0 alert	0 alert	0 alert	0 alert	0 alert	0 alert
Leadlikeness	Yes	Yes	Yes	Yes	Yes	Yes	Yes	Yes	Yes	Yes	No; 1 viol Rotors>7	Yes	Yes	Yes
Synthetic accessibility	4.64	4.30	4.15	5.08	5.19	4.34	4.34	4.02	3.92	4.69	4.80	5.50	4.29	3.91

Figure S14. Boiled-Egg representation of calculated BBB permeability of the set of compounds obtained. In the yellow area, the compounds that would be able to cross the BBB are grouped, while in the white area, those that would not cross it.

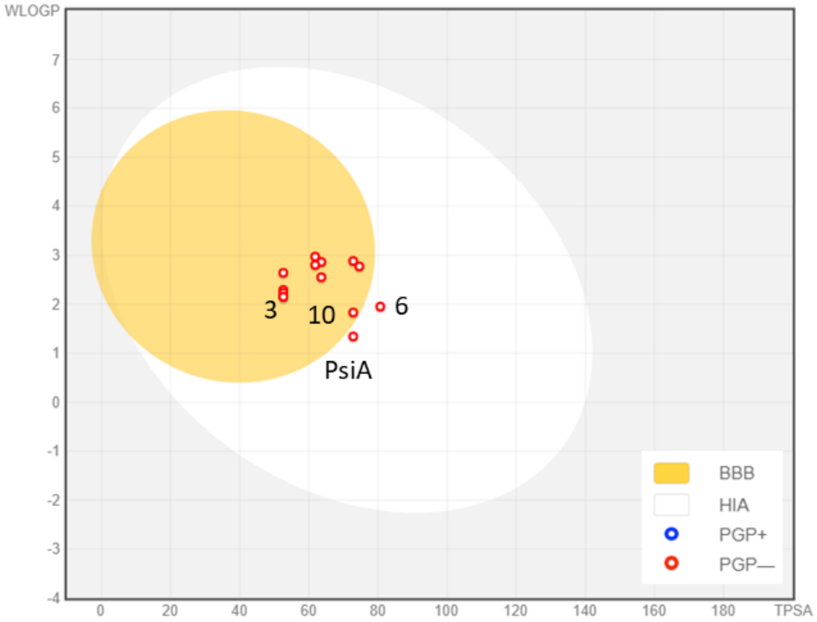

Table S3. Parameters obtained by the Quasar-contingency tool in the MOE program for the 83 selected molecular descriptors, with cutoff values displayed in the first row below the parameter names.

**Contingency Coefficient** (above 0.6 is useful)	**Cramer's V** (above 0.2 is useful)	**Entropic Uncertainty** (above 0.2 is useful)	**Linear Correlation R**2** (above 0.2 is useful)	**Molecular Descriptor from the MOE program**
0.86855	0.50592	0.51223	0.29177	apol
0.84627	0.45856	0.50299	0.25109	ASA_H
0.7564	0.33381	0.31771	0.24521	ast_violation
0.83659	0.44083	0.39241	0.29735	ast_violation_ext
0.82256	0.41755	0.47202	0.28884	a_count
0.93222	0.27306	0.66397	0.2891	a_heavy
0.9288	0.35805	0.65291	0.36745	a_hyd
0.78409	0.36469	0.38396	0.21118	a_IC
0.924	0.32883	0.62234	0.37342	a_nC
0.92473	0.26668	0.6241	0.27228	a_nH
0.86949	0.36705	0.50777	0.24605	a_nO
0.82125	0.4155	0.35938	0.23096	balabanJ
0.83279	0.43427	0.43676	0.24018	BCUT_PEOE_0
0.83656	0.44077	0.44023	0.36553	BCUT_PEOE_3
0.8255	0.42221	0.41665	0.40789	BCUT_SLOGP_3
0.82366	0.41928	0.40033	0.24296	BCUT_SMR_0
0.80551	0.3924	0.39714	0.37842	BCUT_SMR_3
0.82991	0.42942	0.48213	0.26543	bpol
0.86799	0.50458	0.51323	0.30671	b_count
0.93163	0.26302	0.6677	0.32046	b_heavy
0.82343	0.41891	0.40985	0.30174	b_single
0.81525	0.40639	0.4338	0.2731	chi0
0.8203	0.41404	0.47105	0.27534	chi0v
0.88474	0.54798	0.53902	0.36735	chi0v_C
0.84491	0.45598	0.49077	0.36793	chi0_C
0.85036	0.46651	0.49099	0.29165	chi1
0.82711	0.42481	0.45986	0.26392	chi1v
0.83529	0.43856	0.41628	0.42305	chi1v_C
0.84847	0.46279	0.45266	0.42161	chi1_C
0.83688	0.33362	0.44149	0.29033	chiral
0.85934	0.48507	0.42285	0.5092	E
0.7945	0.37768	0.3244	0.29419	E_ang
0.86793	0.50444	0.47379	0.42128	E_nb
0.84032	0.4475	0.34762	0.3537	E_oop
0.80679	0.39419	0.3201	0.2157	E_stb
0.848	0.46187	0.372	0.4837	E_str
0.86554	0.49887	0.47659	0.44553	E_vdw
0.85567	0.47729	0.45742	0.53608	GCUT_PEOE_3
0.87696	0.52678	0.50203	0.51877	GCUT_SLOGP_3
0.85811	0.48243	0.46109	0.52762	GCUT_SMR_3
0.82231	0.41717	0.39876	0.24377	h_emd_C
0.83915	0.44537	0.47316	0.26715	h_mr
0.81225	0.40197	0.39653	0.2243	Kier1
0.81574	0.40711	0.38219	0.21707	KierA1
0.69956	0.28261	0.32668	0.2566	lip_violation
0.85048	0.46675	0.46169	0.26906	mr
0.92545	0.36413	0.62616	0.45723	opr_brigid

0.86858	0.50598	0.46918	0.39003	opr_nring
0.74216	0.31966	0.35657	0.2346	opr_violation
0.81372	0.40413	0.40117	0.21718	PEOE_RPC+
0.87683	0.52645	0.50133	0.43628	PEOE_RPC-
0.79053	0.37263	0.34369	0.29902	PEOE_VSA+0
0.79889	0.38342	0.38962	0.38168	PEOE_VSA+5
0.85254	0.47086	0.47575	0.2548	PEOE_VSA_HYD
0.82152	0.41593	0.44362	0.26522	PEOE_VSA_NEG
0.84464	0.45546	0.41183	0.32374	pmiZ
0.8225	0.41745	0.45774	0.22508	Q_RPC+
0.83712	0.44176	0.44213	0.37387	Q_RPC-
0.83352	0.43552	0.4509	0.30057	Q_VSA_HYD
0.83426	0.43678	0.45066	0.219	Q_VSA_POS
0.86377	0.49486	0.46483	0.42195	rings
0.8225	0.41745	0.45774	0.22508	RPC+
0.83712	0.44176	0.44213	0.37387	RPC-
0.82775	0.42586	0.41538	0.23556	SlogP_VSA2
0.83344	0.43537	0.36259	0.20088	SlogP_VSA9
0.83826	0.44379	0.4471	0.26952	SMR
0.85862	0.48352	0.49323	0.2329	SMR_VSA5
0.81297	0.40303	0.38844	0.28271	std_dim3
0.8671	0.50249	0.48156	0.37122	VAdjEq
0.87325	0.51733	0.53294	0.3979	VAdjMa
0.88554	0.55027	0.5257	0.3895	VDistMa
0.80415	0.39053	0.45602	0.24006	vdw_area
0.8387	0.44458	0.46683	0.28563	vdw_vol
0.82604	0.42309	0.46198	0.27655	vol
0.83303	0.43468	0.5117	0.23677	VSA
0.81954	0.41287	0.40019	0.31003	vsa_hyd
0.8694	0.50793	0.54159	0.24795	vsurf_CW1
0.85694	0.47995	0.49068	0.46449	vsurf_R
0.82006	0.41367	0.4521	0.20101	vsurf_S
0.81141	0.40075	0.44241	0.24625	vsurf_V
0.81657	0.40836	0.42836	0.25195	Weight
0.88351	0.54449	0.53008	0.42058	weinerPol
0.85569	0.47733	0.49106	0.35399	zagreb

Table S4. Parameters extracted from principal component analysis (PCA) of the 83 selected molecular descriptors.

Results of PCA	***PC1***	***PC2***
Proportion of variance	58.02	6.287
Cumulative proportion of variance	69.91%	7.57%
Eigenvalue	69.91%	77.48%
**Loadings of each molecular descriptor to the PCs.**		
apol	0.9921	-0.0166
ASA_H	0.8787	-0.1130
ast_violation	0.6118	-0.4207
ast_violation_ext	0.6630	-0.4243
a_count	0.9853	0.0391
a_heavy	0.9788	-0.1432
a_hyd	0.9573	-0.0405
a_IC	0.9549	-0.1318
a_nC	0.9853	-0.0270
a_nH	0.9394	0.2012
a_nO	0.7200	0.0414
balabanJ	-0.5658	0.2273
BCUT_PEOE_0	-0.5701	-0.6564
BCUT_PEOE_3	0.5008	0.6759
BCUT_SLOGP_3	0.4543	0.7730
BCUT_SMR_0	-0.5562	-0.6355
BCUT_SMR_3	0.5010	0.7233
bpol	0.9638	0.0856
b_count	0.9893	0.0382
b_heavy	0.9806	-0.1273
b_single	0.9357	0.2228
chi0	0.9755	-0.1332
chi0v	0.9873	-0.0671
chi0v_C	0.9804	0.0616
chi0_C	0.9767	0.0006
chi1	0.9775	-0.1574
chi1v	0.9845	-0.0323
chi1v_C	0.9294	0.2129
chi1_C	0.9241	0.0793
chiral	0.7357	0.3646
E	0.8058	0.1573
E_ang	0.6658	0.1427
E_nb	0.6944	0.1489
E_oop	0.5701	0.0757
E_stb	0.6041	0.0770
E_str	0.7909	0.1837
E_vdw	0.9199	-0.1138
GCUT_PEOE_3	0.7887	0.5485
GCUT_SLOGP_3	0.7819	0.5259
GCUT_SMR_3	0.8013	0.5217
h_emd_C	0.8324	-0.0997
h_mr	0.9823	-0.1342
Kier1	0.9564	-0.1713
KierA1	0.9510	-0.0463
lip_violation	0.7943	-0.1350
mr	0.9819	-0.1313
opr_brigid	0.8678	-0.0366
opr_nring	0.7640	0.0083

opr_violation	0.8427	-0.1990
PEOE_RPC+	-0.6682	0.3204
PEOE_RPC-	-0.7141	0.2947
PEOE_VSA+0	0.7798	0.2822
PEOE_VSA+5	0.4550	0.2287
PEOE_VSA_HYD	0.9416	-0.1067
PEOE_VSA_NEG	0.8334	-0.0588
pmiZ	0.7494	0.0052
Q_RPC+	-0.5349	0.4761
Q_RPC-	-0.4560	0.5487
Q_VSA_HYD	0.8982	-0.0275
Q_VSA_POS	0.9467	0.0858
rings	0.7855	0.0146
RPC+	-0.5349	0.4761
RPC-	-0.4560	0.5487
SlogP_VSA2	0.7216	0.1489
SlogP_VSA9	0.5553	0.2602
SMR	0.9799	-0.1395
SMR_VSA5	0.6656	-0.1758
std_dim3	0.6605	0.2322
VAdjEq	-0.9005	0.2314
VAdjMa	0.9358	-0.1701
VDistMa	0.9499	-0.1771
vdw_area	0.9712	-0.1162
vdw_vol	0.9903	-0.0728
vol	0.9881	-0.0747
VSA	0.9758	-0.1137
vsa_hyd	0.9164	-0.0640
vsurf_CW1	-0.7455	-0.0556
vsurf_R	0.8643	0.3120
vsurf_S	0.9477	-0.2209
vsurf_V	0.9758	-0.1338
Weight	0.9658	-0.1517
weinerPol	0.9701	0.0398
zagreb	0.9815	-0.0747

Figure S15. Chemical structures of the compounds used in the anti-T98G library.

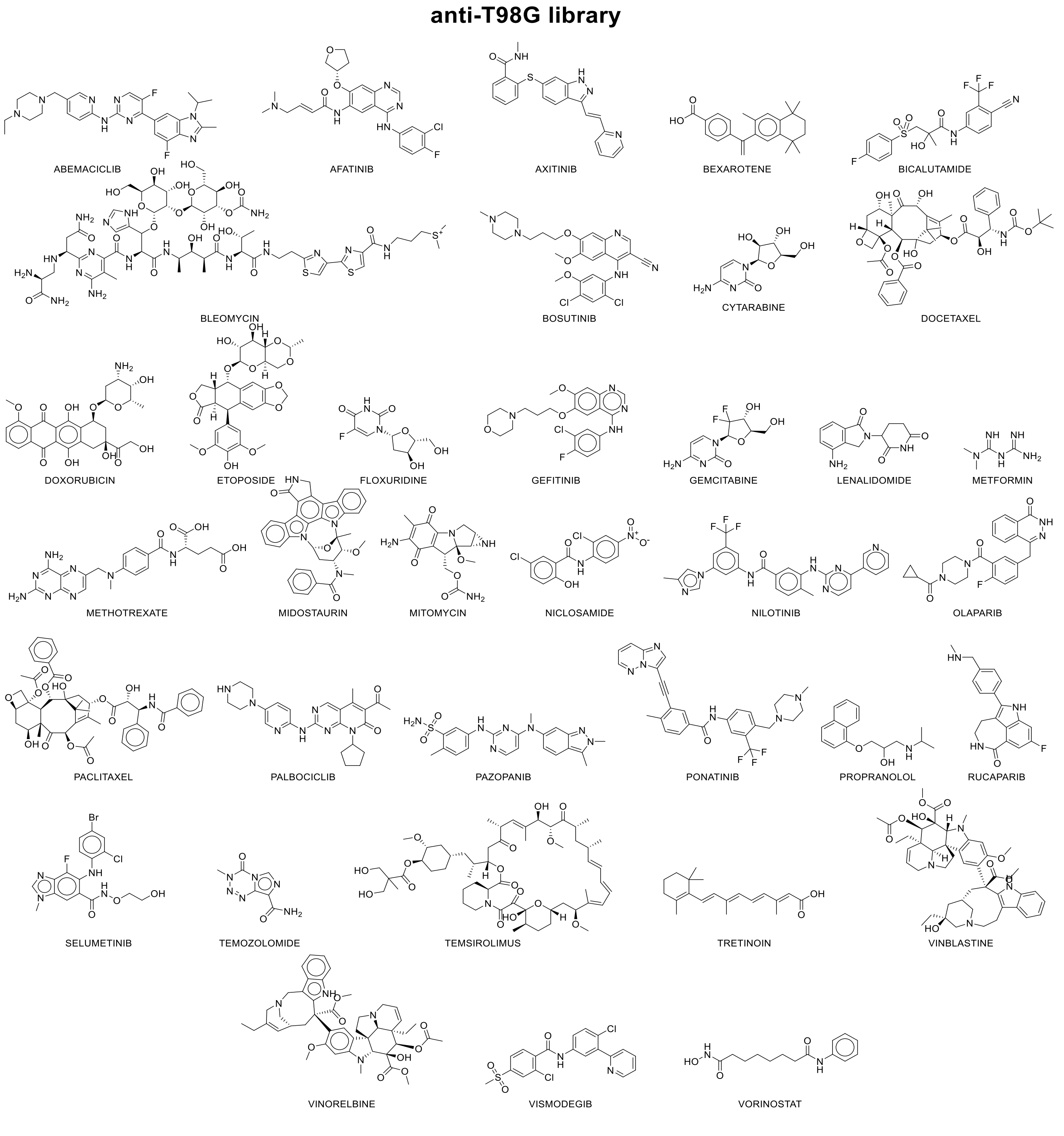

References:

Gaulton, A., et.al. The ChEMBL database in 2017. *Nucleic acids research*, 45(D1), D945-D954 (2017).

Figure S16. Chemical structures of the compounds used in the Cli. Ph. Library.

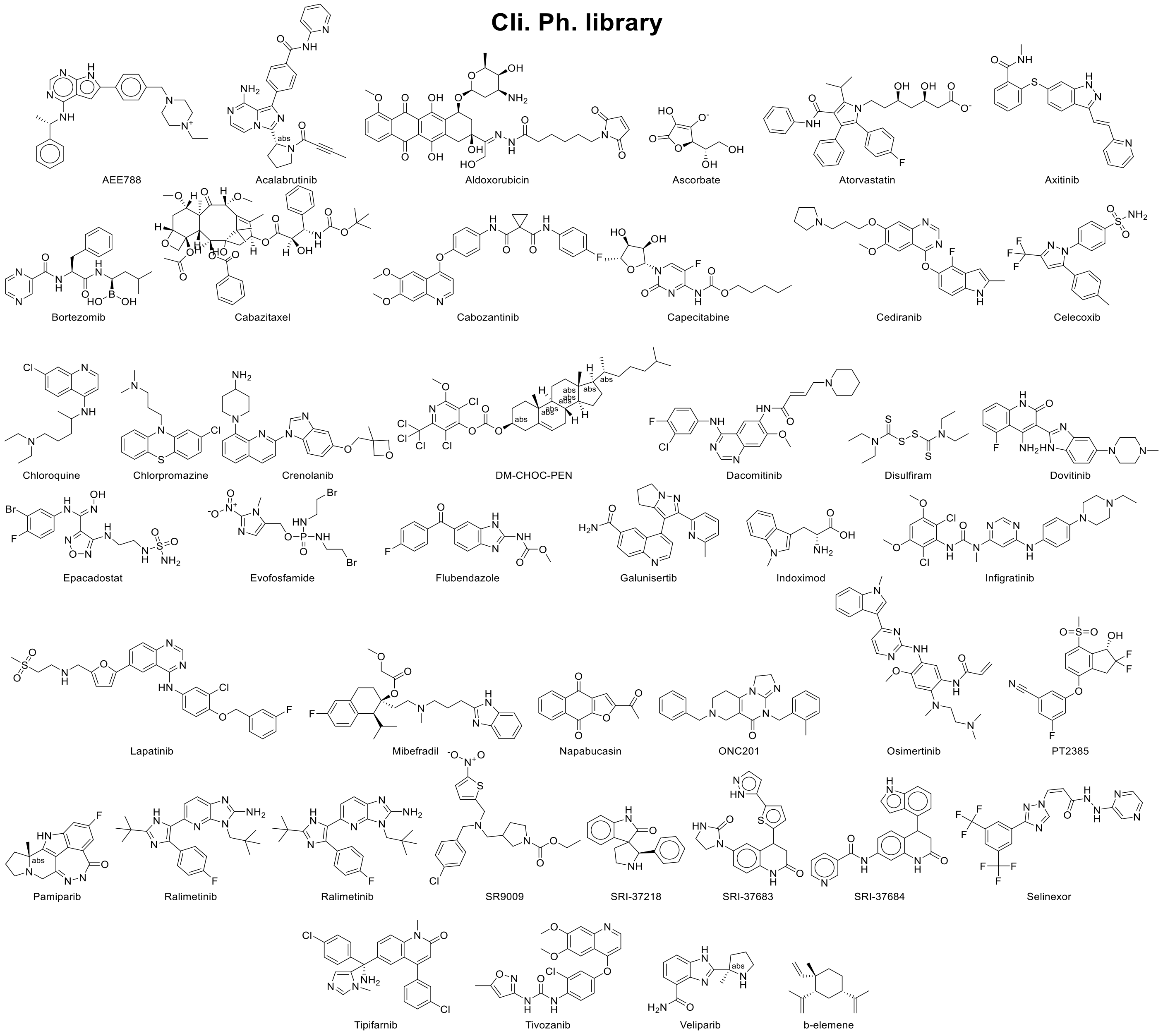

References:

- Vítovcová, B., Skarková, V., Havelek, R., Soukup, J., Pande, A., Caltová, K. & Rudolf, E. Flubendazole exhibits anti-glioblastoma effect by inhibiting STAT3 and promoting cell cycle arrest. *Sci. Rep.* 13:5993 (2023).
- Liu, X., et.al. Valtrate, an iridoid compound in Valeriana, elicits anti-glioblastoma activity through inhibition of the PDGFRA/MEK/ERK signaling pathway. J. Transl. Med. 21:147 (2023).
- Kim, H. & Kim, D. Present and Future of Anti-Glioblastoma Therapies: A Deep Look into Molecular Dependencies/Features. Molecules. 25, 4641 (2020).
- Styczynski, J., Olszewska-Slonina, D., Kolodziej, B., Napieraj, M., Wysocki, M. Activity of Bortezomib in Glioblastoma. *Anticancer Res.* 26: 4499-4504 (2006).
- Landis, C. Identification of Compounds That Decrease Glioblastoma Growth and Glucose Uptake in Vitro. *ACS Chem. Biol.* 13, 2048-2057 (2018).
- Thakur, A. et. al. Glioblastoma: Current Status, Emerging Targets, and Recent Advances. *J. Med. Chem*., 65, 8596-8685 (2022).

Figure S17. Chemical structures of the compounds used in the NP library.

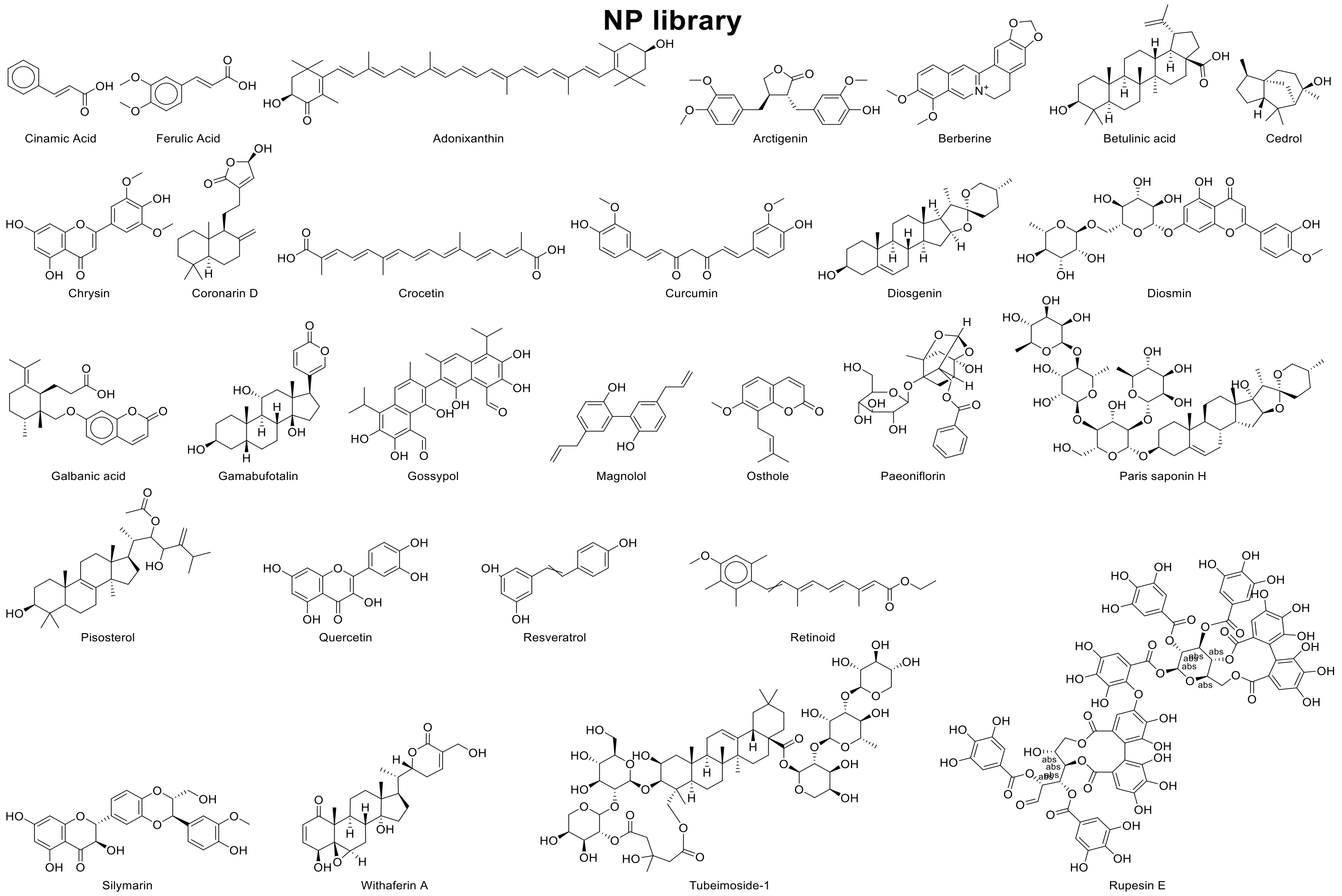

References:

- Tapan Behl, et.al. Current Perspective on the Natural Compounds and Drug Delivery Techniques in Glioblastoma Multiforme. *Cancers*. 13, 2765 (2021).
- Persano, F., Gigli, G., & Leporatti, S. Natural Compounds as Promising Adjuvant Agents in The Treatment of Gliomas. *Int. J. Mol. Sci.* 23, 3360 (2022).
- Zhai, K., Siddiqui, M., Abdellatif, B.,Liskova, A., Kubatka, P., & Büsselberg, D. Natural Compounds in Glioblastoma Therapy: Preclinical Insights, Mechanistic Pathways, and Outlook. *Cancers*, *13*(10), 2317. (2021).

Table S5. Libraries of compounds with anti-glioblastoma properties: psilostachyins and their derivatives obtained in acid medium (Psi. D.), Approved drugs with IC_{50} reported against T98G line (anti-T98G); Compounds with anti-glioblastoma studies in clinical phase (Cli. Ph.) and Natural products with such bioactivities (NP). Principal components values (PC) and their cluster classification are also detailed for each compound.

Compounds:	Library	PC 1	PC 2	Cluster
PsiA	Psi. D.	-0.560	1.553	A
PsiB	Psi. D.	-0.649	1.208	A
PsiC	Psi. D.	-0.599	1.778	A
1	Psi. D.	-0.715	0.987	A
2	Psi. D.	-0.654	1.318	A
3	Psi. D.	-0.657	1.292	A
4	Psi. D.	-0.659	1.299	A
5	Psi. D.	-0.554	1.189	A
6	Psi. D.	-0.645	1.546	A
7	Psi. D.	-0.504	0.708	A
8	Psi. D.	-0.363	0.493	A
9	Psi. D.	-0.290	0.969	A
10	Psi. D.	-0.456	1.467	A
11	Psi. D.	-0.723	1.455	A
Abemaciclib	anti-T98G	0.502	-0.456	B
Afatinib	anti-T98G	0.144	-0.818	C
Axitinib	anti-T98G	-0.232	-1.497	C
Bexarotene	anti-T98G	-0.080	0.999	A
Bicalutamide	anti-T98G	-0.283	-0.570	C
Bleomycin	anti-T98G	3.427	-1.559	D
Bosutinib	anti-T98G	0.469	-0.522	B
Cytarabine	anti-T98G	-1.028	-0.139	C
Docetaxel	anti-T98G	2.097	0.488	B
Doxorubicin	anti-T98G	0.543	-0.232	B
Etoposide	anti-T98G	0.764	-0.087	B
Floxuridine	anti-T98G	-1.079	0.090	C
Gefitinib	anti-T98G	0.032	-0.464	C
Gemcitabine	anti-T98G	-1.033	-0.094	C
Lenalidomide	anti-T98G	-0.857	0.138	C
Metformin	anti-T98G	-1.707	-0.617	C
Methotrexate	anti-T98G	-0.059	-1.158	C
Midostaurin	anti-T98G	1.096	0.147	B
Mitomycin	anti-T98G	-0.435	0.427	A
Niclosamide	anti-T98G	-0.843	-1.169	C
Nilotinib	anti-T98G	0.354	-1.714	C
Olaparib	anti-T98G	0.048	-0.122	C
Paclitaxel	anti-T98G	2.264	0.288	B

Palbociclib	anti-T98G	0.228	-0.168	C
Pazopanib	anti-T98G	-0.103	-1.251	C
Ponatinib	anti-T98G	0.529	-0.543	B
Propranolol	anti-T98G	-0.678	0.256	C
Rucaparib	anti-T98G	-0.490	-0.301	C
Selumetinib	anti-T98G	-0.324	-1.184	C
Temozolomide	anti-T98G	-1.456	-0.680	C
Temsirolimus	anti-T98G	3.073	-0.254	D
Tretinoin	anti-T98G	-0.403	0.960	A
Vinblastine	anti-T98G	2.239	0.631	E
Vinorelbine	anti-T98G	2.104	0.583	E
Vismodegib	anti-T98G	-0.296	-1.088	C
Vorinostat	anti-T98G	-0.773	-0.033	C
Aee788	Cli. Ph.	0.331	-0.412	C
Acalabrutinib	Cli. Ph.	0.235	-0.852	C
Aldoxorubicin	Cli. Ph.	1.394	-0.731	B
Ascorbate	Cli. Ph.	-1.526	0.095	C
Atorvastatin	Cli. Ph.	0.712	-0.565	B
Axitinib	Cli. Ph.	-0.234	-1.500	C
Bortezomib	Cli. Ph.	-0.141	-0.370	A
Cabazitaxel	Cli. Ph.	2.113	0.488	C
Cabozantinib	Cli. Ph.	0.397	-1.119	B
Capecitabine	Cli. Ph.	-0.448	-0.324	C
Cediranib	Cli. Ph.	0.201	-0.480	C
Celecoxib	Cli. Ph.	-0.501	-0.812	C
Chloroquine	Cli. Ph.	-0.358	0.583	C
Chlorpromazine	Cli. Ph.	-0.478	0.151	A
Crenolanib	Cli. Ph.	0.262	-0.351	C
Dm-Choc-Pen	Cli. Ph.	1.339	1.141	C
Dacomitinib	Cli. Ph.	0.167	-0.541	C
Disulfiram	Cli. Ph.	-0.844	0.436	A
Dovitinib	Cli. Ph.	-0.064	-0.253	E
Epacadostat	Cli. Ph.	-0.550	-1.008	C
Evofosfamide	Cli. Ph.	-0.754	-0.079	C
Flubendazole	Cli. Ph.	0.076	0.279	C
Galunisertib	Cli. Ph.	-0.157	-0.555	C
Indoximod	Cli. Ph.	-1.065	0.187	C
Infigratinib	Cli. Ph.	0.540	-0.788	C
Lapatinib	Cli. Ph.	0.537	-1.456	B
Mibefradil	Cli. Ph.	0.607	0.220	B
Napabucasin	Cli. Ph.	-1.073	-0.636	B
Onc201	Cli. Ph.	0.025	-0.199	C
Osimertinib	Cli. Ph.	0.518	-0.880	C
Pt2385	Cli. Ph.	-0.435	-0.359	B
Pamiparib	Cli. Ph.	-0.565	0.880	A

Ralimetinib	Cli. Ph.	0.176	-0.178	C
Regorafenib	Cli. Ph.	-0.150	-1.525	C
Sr9009	Cli. Ph.	-0.033	0.046	C
Sri-37218	Cli. Ph.	-0.632	0.576	C
Sri-37683	Cli. Ph.	-0.301	-0.707	C
Sri-37684	Cli. Ph.	-0.141	-0.614	A
Selinexor	Cli. Ph.	-0.365	-1.509	C
Tipifarnib	Cli. Ph.	0.337	-0.589	C
Tivozanib	Cli. Ph.	-0.039	-1.671	C
Veliparib	Cli. Ph.	-0.841	0.620	C
B-Elemene	Cli. Ph.	-0.629	0.905	A
Cinamic Acid	NP	-1.609	-0.425	C
Ferulic Acid	NP	-1.251	-0.350	C
Adonixanthin	NP	1.184	-0.468	B
Arctigenin	NP	-0.172	-0.055	C
Berberine	NP	-0.315	-0.656	C
Betulinic Acid	NP	0.461	2.464	E
Cedrol	NP	-0.818	4.232	f
Chrysin	NP	-0.976	-0.632	C
Coronarin D	NP	-0.190	1.735	A
Crocetin	NP	-0.554	-1.122	C
Curcumin	NP	-0.373	-1.392	C
Diosgenin	NP	0.436	1.909	E
Diosmin	NP	0.678	-0.872	B
Galbanic Acid	NP	0.061	1.116	A
Gamabufotalin	NP	0.250	1.657	A
Gossypol	NP	0.506	-0.703	B
Magnolol	NP	-0.679	-0.741	C
Osthole	NP	-0.878	0.072	C
Paeoniflorin	NP	0.361	0.550	A
Paris Saponin H	NP	2.907	0.400	D
Pisosterol	NP	1.012	1.669	E
Quercetin	NP	-0.778	-0.756	C
Resveratrol	NP	-1.081	-1.098	C
Retinoid	NP	-0.155	-0.500	C
Rupesin E	NP	-0.552	1.185	A
Silymarin	NP	0.202	-0.884	C
Tubeimoside-1	NP	4.361	1.176	G
Withaferin A	NP	0.591	1.397	E

Figure S18. Clustering of library compounds in a two-dimensional chemical space using the first two principal components.

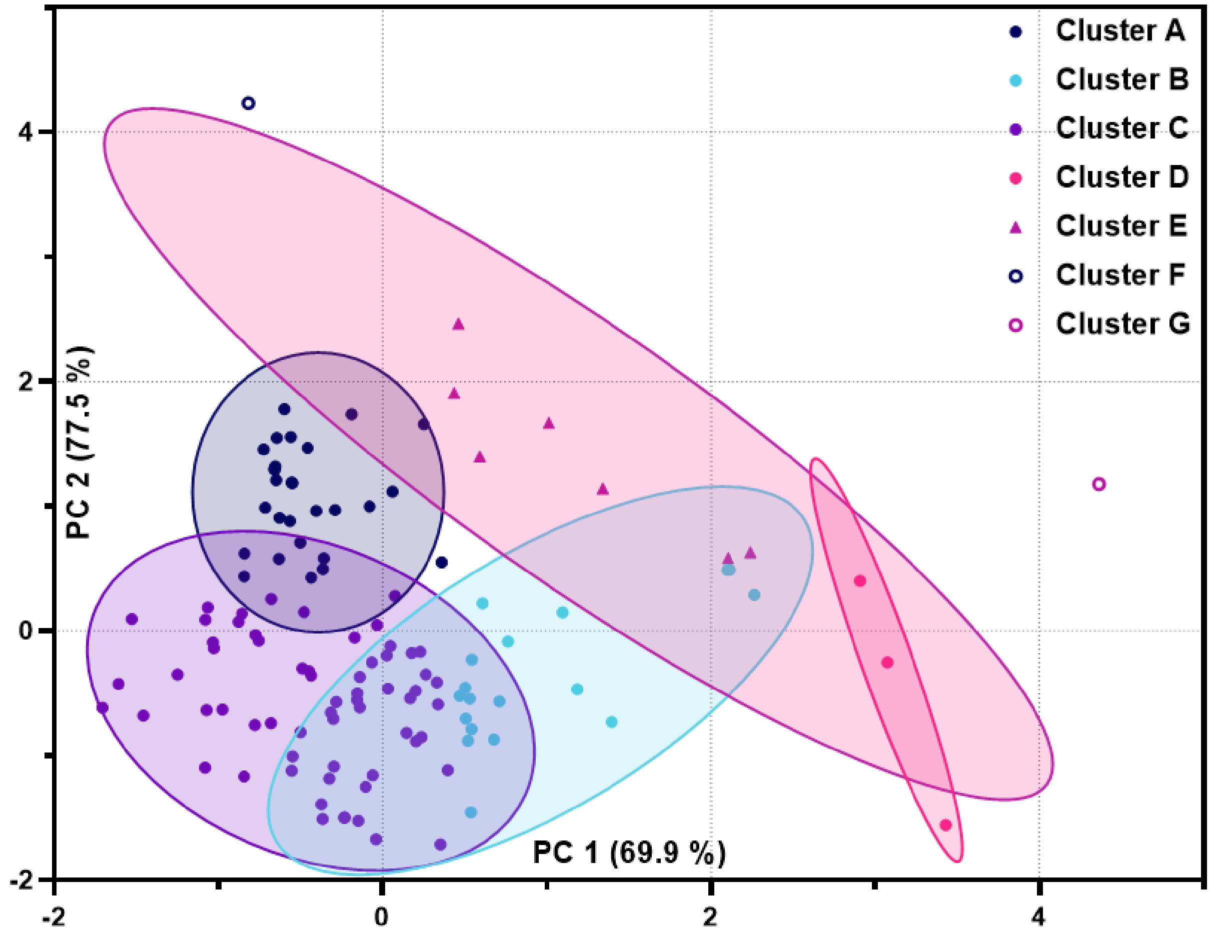

Supplement: Supplementary file 1 — Supplementary Information. [file 41598_2024_63639_MOESM1_ESM.pdf]
